# Revision of the World species of the genus *Chromoteleia* Ashmead (Hymenoptera, Platygastridae, Scelioninae)

**DOI:** 10.3897/zookeys.778.25775

**Published:** 2018-08-01

**Authors:** Hua-yan Chen, Elijah J. Talamas, Alejandro A. Valerio, Lubomír Masner, Norman F. Johnson

**Affiliations:** 1 Department of Entomology, The Ohio State University, 1315 Kinnear Road, Columbus, Ohio 43212, USA The Ohio State UniversityColumbusUnited States of America; 2 Florida Department of Agriculture and Consumer Services, The Doyle Conner Building, 1911 SW 34th St, Gainesville, Florida 32608, USA Florida Department of Agriculture and Consumer ServicesGainesvilleUnited States of America; 3 Central American Institute for Biological Research and Conservation (CIBRC), P.O. Box 2398-2050 San Pedro de Montes de Oca, San Jose, Costa Rica Central American Institute for Biological Research and ConservationSan JoseCosta Rica; 4 Agriculture and Agri-Food Canada, K.W. Neatby Building, Ottawa, Ontario K1A 0C6, Canada Agriculture and Agri-Food CanadaOttawaCanada; 5 Department of Evolution, Ecology and Organismal Biology, The Ohio State University, 1315 Kinnear Road, Columbus, Ohio 43212, USA

**Keywords:** identification key, Platygastroidea, species descriptions

## Abstract

The genus *Chromoteleia* Ashmead is revised. Twenty-seven species are recognized, of which six species are redescribed: *C.congoana* (Risbec), *C.connectens* Kieffer, *C.fuscicornis* Kieffer, *C.longitarsis* Kieffer, *C.semicyanea* Ashmead, *C.tricarinata* Kieffer; and twenty-one species are described as new: *C.aequalis* Chen & Johnson, **sp. n.**, *C.alternata* Chen & Johnson, **sp. n.**, *C.bidens* Chen & Masner, **sp. n.**, *C.copiosa* Chen & Johnson, **sp. n.**, *C.cuneus* Chen & Johnson, **sp. n.**, *C.curta* Chen & Johnson, **sp. n.**, *C.depilis* Chen & Johnson, **sp. n.**, *C.dispar* Chen & Masner, **sp. n.**, *C.feng* Chen & Johnson, **sp. n.**, *C.fossa* Chen & Johnson, **sp. n.**, *C.ingens* Chen & Masner, **sp. n.**, *C.levitas* Chen & Johnson, **sp. n.**, *C.longa* Chen & Johnson, **sp. n.**, *C.maura* Chen & Masner, **sp. n.**, *C.parvitas* Chen & Johnson, **sp. n.**, *C.pilus* Chen & Johnson, **sp. n.**, *C.plana* Chen & Johnson, **sp. n.**, *C.rara* Chen & Johnson, **sp. n.**, *C.robusta* Chen & Johnson, **sp. n.**, *C.semilutea* Chen & Johnson, **sp. n.**, *C.sparsa* Chen & Johnson, **sp. n.***Chromoteleiarufithorax* Kieffer remains a valid species, but its identity and status are unclear. All species are known only from the Neotropical region except for *Chromoteleiacongoana* (Resbec) which only occurs in Africa.

## Introduction

The genus *Chromoteleia* was originally described by Ashmead (1893) based on the colorful type species *Chromoteleiasemicyanea* Ashmead collected in Saint Vincent. Later, Kieffer (1926) proposed *Petalosema* for species with expanded metanotum (metascutellum). Masner (1976) synonymized *Petalosema* with *Chromoteleia* by pointing out that Kieffer (1926) was misled by Ashmead’s (1893) inaccurate illustration of *C.semicyanea* showing a narrow and unexpanded metanotum. Six species were recorded from the New World: *C.connectens* Kieffer, *C.fuscicornis* Kieffer, *C.longitarsis* Kieffer, *C.rufithorax* Kieffer, *C.semicyanea* Ashmead, and *C.tricarinata* Kieffer. One species also was described from the Afrotropical region, *C.congoana* (Risbec), from what is currently Gabon. One fossil species, *C.theobaldi* Maneval, is reported from Baltic amber (from 40~60 mya) (Maneval 1938), but the status of this species is not clear (Talamas and Buffington 2015) and requires direct examination.

Since its original description in 1893, *Chromoteleia* has never been comprehensively reviewed. Although there are no host records for *Chromoteleia*, we suspect that it parasitizes the eggs of Orthoptera based on large size and elongate shape of the parasitoids. A number of scelionine genera of similar habitus, and presumed close relation, are also known to be parasitoids of orthopteran eggs, e.g., *Macroteleia* Kieffer (Muesebeck, 1977), *Triteleia* Kieffer (Popovici et al. 2011). The goal of this work is to produce a systematic revision of the world species of *Chromoteleia*, expand the biogeographic data associated with these species, and to present a clarified generic concept. The contributions of the authors are as follows. H.-y. Chen, E. J. Talamas, A. A. Valerio and N.F. Johnson: character definition, generic concept development, species concept development, imaging, key development, manuscript preparation; L. Masner: character definition, generic concept development, species concept development.

## Materials and methods

This work is based upon specimens in the following collections, with abbreviations used in the text:

**AEIC**American Entomological Institute, Gainesville, FL;

**AMNH**American Museum of Natural History, New York, NY;

**BPBM** Bernice P. Bishop Museum, Honolulu, HI;

**CAS**California Academy of Sciences, San Francisco, CA;

**CNCI**Canadian National Collection of Insects, Ottawa, Canada;

**CSCA**California State Collection of Arthropods, Sacramento, CA;

**FSCA**Florida State Collection of Arthropods, Gainesville, FL;

**IAVH**Instituto Alexander von Humboldt, Colección de Artrópodos, Villa de Leyva, Colombia;

**INHS**Illinois Natural History Survey, Urbana, IL;

**KUNH** Kansas University Natural History Museum, Lawrence, KS;

**MIZA**Museo del Instituto de Zoología Agrícola, Maracay, Venezuela;

**MNHN**Muséum National d’Histoire Naturelle, Paris, France;

**MPEG**Museu Paraense Emílio Goeldi, Belém, PA, Brazil;

**MZLU** Lund Museum of Zoology, Lund University, Lund, Sweden;

**OSUC** C.A. Triplehorn Insect Collection, Ohio State University, Columbus, OH;

**SAMC** South African Museum, Iziko Museums of Cape Town, South Africa;

**TAMU**Texas A&M University Insect Collection, College Station, TX;

**UCDC**R.M. Bohart Museum of Entomology, University of California, Davis, CA;

**UCMC**University of Colorado, Boulder, CO;

**USNM**National Museum of Natural History, Washington, DC.

Abbreviations and morphological terms used in text:

**A1, A2, ... A12** antennomere 1, 2, … 12;

**claval formula** distribution of the large, multiporous basiconic sensilla on the underside of apical antennomeres of the female, with the segment interval specified followed by the number of sensilla per segment (Bin 1981);

**EH** eye height, length of compound eye measured parallel to dorsoventral midline of head;

**IOS** interocular space, minimal distance on frons between compound eyes;

**OD** ocellar diameter, greatest width of ocellus;

**OOL** ocular ocellar line, shortest distance from inner orbit and outer margin of posterior ocellus (Masner 1980);

**T1, T2, ... T7** metasomal tergite 1, 2, ... 7;

**S1, S2, … S7** metasomal sternite 1, 2, … 7.

Morphological terminology otherwise generally follows Masner (1980) and Mikó et al. (2007). Morphological terms used in this work were as in the Hymenoptera Anatomy Ontology (Yoder et al. 2010) (Appendix 1). Identifiers (URIs) in the format HAO_XXXXXXX represent concepts in the HAO and are provided to enable readers to confirm their understanding of the concepts being referenced. To find out more about a given concept, including additional images, notes, references and other metadata, use the identifier as a search term at http://glossary.hymao.org or use the identifier as a web-link.

In the Material Examined section the specimens studied are recorded in an abbreviated format, using unique identifiers (numbers prefixed with “OSUC”, “CASENT”, “FBA”, “MNHN_EY”) for the individual specimens. The label data for all specimens have been georeferenced and recorded in the Hymenoptera Online database, and details on the data associated with these specimens can be accessed at the following link, hol.osu.edu, and entering the identifier in the form (note the space between the acronym and the number). The electronic version of the paper contains hyperlinks to external resources. Insofar as possible, the external information conforms to standards developed and maintained through the organization Biodiversity Information Standards (Taxonomic Database Working Group). All new species have been prospectively registered with ZooBank (Polaszek et al. 2005, www.zoobank.org), and other taxonomic names, where appropriate, have been retrospectively registered. The external hyperlinks are cited explicitly in the endnotes so that users of the printed version of this article have access to the same resources.

Data associated with the genus *Chromoteleia* can be accessed at http://hol.osu.edu/index.html?id=464. The generic and species descriptions were generated by a database application, vSysLab (vsyslab.osu.edu), designed to facilitate the production of a taxon by character data matrices, and to integrate those data with the existing taxonomic and specimen-level database. Data may be exported in both text format and as input files for other applications. The text output for descriptions is in the format of “Character: Character state (s)”. Polymorphic characters are indicated by semicolon-separated character states.

Images and measurements were made using Combine ZP and AutoMontage extended-focus software, using JVC KY-F75U digital camera, Leica Z16 APOA microscope, and 1X objective lens. Images were post-processed with Abobe Photoshop CS3 Extended. A standard set of images is provided for each species: dorsal habitus, lateral habitus, dorsal and lateral views of the head and mesosoma, and anterior view of head. The individual images are archived in Specimage (specimage.osu.edu), the image database at The Ohio State University.

## Taxonomy

### Chromoteleia

Taxon classificationAnimaliaHymenopteraScelionidae

Ashmead

http://zoobank.org/25D9A544-B778-4365-9542-1E942D25AAF9

http://bioguid.osu.edu/xbiod_concepts/464

Chromoteleia Ashmead, 1893: 209, 211, 219 Type: Chromoteleiasemicyanea Ashmead, by monotypy and original designation (keyed); Ashmead 1894: 216 (keyed); Dalla Torre 1898: 501 (catalog of species); Ashmead 1900: 327 (list of species of West Indies); Ashmead, 1903: 91, 93 (keyed); Kieffer 1907: 266 (key to species); Brues 1908: 26, 27, 28, 35 (diagnosis, keyed, list of species); Kieffer 1908b: 115 (keyed); Kieffer 1910a: 312 (key to species); Kieffer 1910b: 62, 68 (description, list of species, keyed); Kieffer 1913: 224 (description); Kieffer 1926: 269, 406 (description, keyed); Muesebeck and Walkley 1956: 342 (citation of type species); Masner 1976: 23, 24 (description, synonymy; key to separate Baryconus Forster, Bracalba Dodd, Chromoteleia Ashmead, Oxyscelio Kieffer); Carpenter 1992: 471 (fossil references); Johnson 1992: 363 (cataloged, catalog of world species); Loiácono and Margaría 2002: 557 (catalog of Brazilian species).
Petalosema Kieffer 1926: 267, 358. Type: Chromoteleiarufithorax Kieffer, by original description (keyed, key to species), designated by Muesebeck and Walkley 1956. Synonymized by Masner (1976); Muesebeck and Walkley 1956: 336 (citation of type species); De Santis 1980: 310: (catalog of species of Brazil).
http://zoobank.org/E5906ABF-3A4D-4005-BFEC-13B2AEBD6E81
http://bioguid.osu.edu/xbiod_concepts/8521

#### Description.

Length 3.38–9.20 mm; body elongate, robust.

*Head*. Head shape in dorsal view: transverse. Vertex: densely punctate to punctate rugose. Hyperoccipital carina: absent. Occipital carina: present, complete or broadly interrupted medially. OOL: lateral ocellus nearly contiguous with inner orbits, OOL < 0.5 OD; lateral ocellus contiguous with inner orbit. Upper frons: convex, without frontal shelf or carina, punctate rugose. Antennal scrobe: broadly convex to concave medially with distinct depression. Submedian carina: absent. Orbital carina: absent. Inner orbits: diverging ventrally. IOS/EH: IOS distinctly less than EH. Interantennal process: short, often excavate medially. Central keel: present or absent. Antennal foramen: oriented laterally on interantennal process. Facial striae: absent. Malar sulcus: present. Malar striae: absent. Setation of compound eye: absent. Gena: broad, convex, distinctly produced behind eye. Clypeus shape: narrow, slightly convex medially, lateral corners not produced. Anterior (or ventral) margin of clypeus: pointed; straight. Labrum: not visible in anterior view. Number of mandibular teeth: 3. Arrangement of mandibular teeth: transverse. Number of maxillary palpomeres: 4. Shape of maxillary palpomeres: cylindrical. Number of labial palpomeres: 2.

*Antenna*. Number of antennomeres in female: 12. Number of antennomeres in male: 12. Insertion of radicle into A1: parallel to longitudinal axis of A1. Shape of A1: more or less cylindrical, not flattened. Length of A3 of female: distinctly longer than A2. Number of antennomeres with basiconic sensilla in female: 5; 6. Number of antennomeres with basiconic sensilla in female: 5; 6. Arrangement of sensilla on female clava: in longitudinal pairs. Number of antennomeres bearing tyloids in male antenna: 1. Shape of male flagellum: filiform.

*Mesosoma*. Posterior apex of pronotum in dorsal view: straight, bifid apically to articulate with tegula. Epomial carina: present. Anterior face of pronotum: oblique, visible dorsally, short. Lateral face of pronotum: weakly concave below position of dorsal epomial carina. Netrion: present. Netrion shape: moderately wide, open ventrally. Anterior portion of mesoscutum: vertical, flexed ventrally to meet pronotum. Mesoscutum shape: pentagonal, excavate at base of wings. Skaphion: absent. *Notauli*: present, percurrent. Parapsidal lines: absent. Antero-admedian lines: absent. Transscutal articulation: well-developed, narrow. Shape of mesoscutellum: trapezoidal, without spines. Lateral mesoscutellar spine: absent. Median mesoscutellar spine: absent. Axillular spine: absent. Surface of mesoscutellum: convex throughout. Median longitudinal furrow on mesoscutellum: absent. Metascutellum: clearly differentiated. Shape of metascutellum: trapezoidal with broad posterior margin; elongate trapezoidal but with deeply incised apex, forming two spines laterally. Posterior margin of metascutellum: straight; concave; convex. Setation of metascutellum: present. *Metapostnotum*: fused to propodeum. Lateral propodeal projection: absent. Medial propodeal projection: absent. Mesopleural carina: present. Mesal course of acetabular carina: not separating fore coxae. Setation of subalar pit: present. Mesopleural pit: present. Posterodorsal corner of mesopleuron: rounded anteriorly.

*Legs*. Number of mesotibial spurs: 1. Number of metatibial spurs: 1. Dorsal surface of metacoxa: smooth; punctate. Shape of metacoxa: cylindrical, ecarinate. Trochantellus: indicated by transverse sulcus on femur.

*Wings*. Wing development of female: macropterous. Wing development of male: macropterous. Tubular veins in fore wing: present. Bulla of fore wing R: absent. Length of marginal vein of fore wing: punctiform, R terminating at costal margin. Origin of r-rs in fore wing: basal of point at which R meets costal margin. Basal vein (Rs+M) in fore wing: spectral; nebulous. Development of R vein in hind wing: complete.

*Metasoma*. Number of external metasomal tergites in female: 6. Number of external metasoma sternites in female: 6. Number of external metasomal tergites in male: 7. Number of external metasomal sternites in male: 7. Shape of metasoma: lanceolate. Laterotergites: present, narrow. Laterosternites: present. T1 of female: flat; medially convex as a small hump anteriorly. Relative size of metasomal segments: T2–T3 subequal in length, remaining terga shorter. Metasomal tergites with basal crenulae: T2. Sublateral carinae on tergites: present. Median longitudinal carina on metasomal terga: absent. Shape of female T6: flattened; laterally compressed. Anterior margin of S1: not produced anteriorly, straight. Felt fields on S2: absent. Felt fields on S3: absent. Ovipositor: *Scelio*-type (Austin and Field 1997).

#### Generic diagnosis.

The large size and distinctive characters of *Chromoteleia* (metascutellum large and setose, propodeum without projections, marginal vein of fore wing punctiform) make it a relatively easy genus to identify. The setation of the metascutellum is found in relatively few scelionine genera, typically among the more robust genera, and is a useful diagnostic character. *Chromoteleia* appears closest to *Bracalba* Dodd and *Romilius* Walker, from which it can be separated by the setation of the eyes (absent in *Chromoteleia*).

#### Comments.

The distribution of *Chromoteleia* in Africa and South America is a phenomenon of biogeographical interest. Dispersal from South America to Africa has been demonstrated in the parasitoid wasp genus *Kapala* Cameron (Eucharitidae) (Murray and Heraty 2016) and a similar event could explain the disjunct distribution of *Chromoteleia*. Alternatively, the dispersal event could have occurred in the opposite direction, followed by radiation in the New World tropics. While there is no direct evidence that the distribution of *Chromoteleia* represents relictual populations, this is likely the case with other scelionine taxa. For example, *Archaeoteleia* Masner, which today is known from New Zealand and South America, has been documented from Burmese amber (Talamas et al. 2017), suggesting that the extant fauna of this genus is the remainder of a once widespread distribution.

*Chromoteleia* is widespread in continental Mesoamerica, Central America, and South America. It is found as far north as the Mexican state of Jalisco, and in the south extends to Itapúa Department in Paraguay and Paraná in southern Brazil. It is noteworthy, though, that it is entirely absent from the Greater Antilles. In the Lesser Antilles, one species, *C.semicyanea*, apparently is endemic in St. Vincent, and a second, *C.aequalis*, is known from Dominica (as well as Guyana). This is unusual for scelionines of comparable size and presumed biology: genera such as *Scelio*, *Baryconus*, *Macroteleia*, *Triteleia* and *Opisthacantha* are common and richly represented in species throughout the Caribbean.

### Character discussion

*Basiconic sensilla on A12*

Seven of the twenty-seven species of *Chromoteleia* clearly have two basiconic sensilla on the apical antennomere (Figure 2), a state that is unknown to us from any other scelionine in which basiconic sensilla are arranged in longitudinal pairs.

**Figures 1–6.**
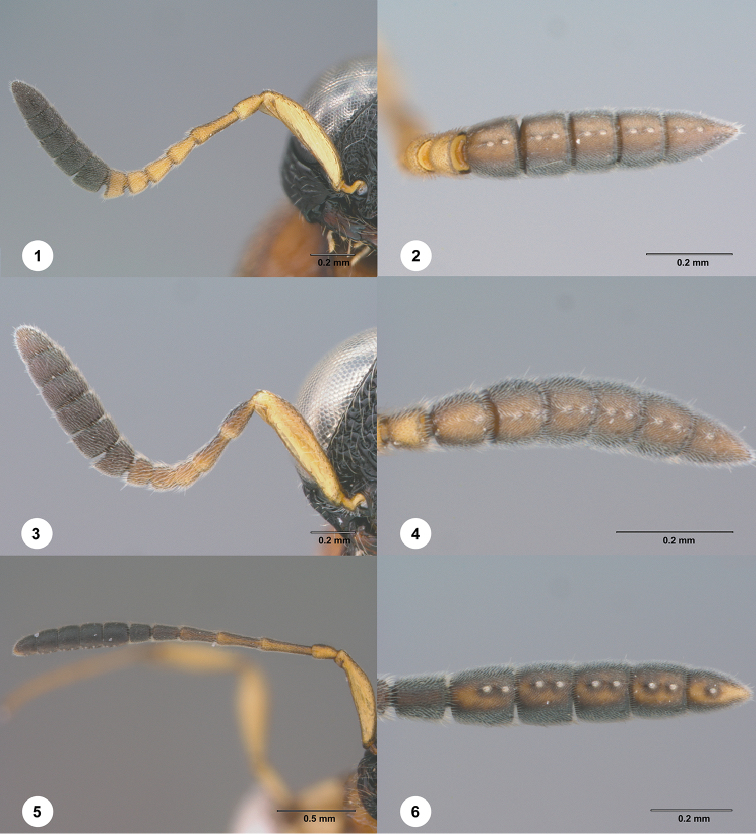
**1–2***Chromoteleiatricarinata* Kieffer, female (OSUC268814). **1** Antenna **2** Antennal clava, ventral view **3–4***Chromoteleiaparvitas* sp. n., holotype, female (OSUC276182) **3** Antenna **4** Antennal clava, ventral view **5–6***Chromoteleialonga* sp. n., holotype, female (OSUC320643) **5** Antenna **6** Antennal clava, ventral view.

### Metapleural setation

The metapleuron in *Chromoteleia* is an important source of characters. In all species there is a line of setigerous foveae along the anterior margin of the metapleuron, corresponding to the dorsal portion of the metapleural sulcus. Directly posterior to this line of setae in the dorsal portion of the sclerite (dorsal metapleural area) there may be patch of setae (Figure 15, black arrow), and its presence or absence separates numerous species.

In the ventral portion of the sclerite, there is often a line of setae (Figure 15, white arrow) directly dorsal to the metapleural triangle. These setae can be distinguished from setation of the metapleural triangle because they do not have foveate bases and are located dorsal to the metapleural epicoxal carina, when this carina is present.

*Setation of speculum and subalar pit*

The presence of setae (Figs 14, 15, blue arrow) on the dorsal speculum and surrounding the subalar pit is found in all species of *Chromoteleia* and serves as a useful character for generic diagnosis (http://specimage.osu.edu/getImageInfo.html?image_id=89044). However, because this is a newly recognized character, it has yet to be examined thoroughly throughout Scelioninae. This character is known to us from *Romilius*, *Bracalba*, and *Macroteleiapilosa* Muesebeck.

*Body color*

Ashmead presumably coined the name *Chromoteleia* in reference to the remarkable metallic blue head and mesosoma in the type species. This turns out to be unique within the genus, but most species are colorful in a different way. Only a small number have the body entirely black or dark brown as is generally typical for other scelionines. Most have more or less extensive parts of the mesosoma colored a rusty red to orange. Masner (1995) and Masner and Hanson (2006) noted that this color pattern is commonly found in a wide range of scelionine genera in species of moderate size (3–10 mm in length) found at lower altitudes (under 2000 m). They attributed the apparent convergent evolution of the color in these taxa to aposematism, a hypothesis that has not yet been critically tested.

### World species of *Chromoteleia* Ashmead

*C.aequalis* Chen & Johnson, sp. n.

*C.alternata* Chen & Johnson, sp. n.

*C.bidens* Chen & Masner, sp. n.

*C.congoana* (Risbec)

*C.connectens* Kieffer

= *C.brevitarsis* Kieffer

*C.copiosa* Chen & Johnson, sp. n.

*C.cuneus* Chen & Johnson, sp. n.

*C.curta* Chen & Johnson, sp. n.

*C.depilis* Chen & Johnson, sp. n.

*C.dispar* Chen & Masner, sp. n.

*C.feng* Chen & Johnson, sp. n.

*C.fossa* Chen & Johnson, sp. n.

*C.fuscicornis* Kieffer

*C.ingens* Chen & Masner, sp. n.

*C.levitas* Chen & Johnson, sp. n.

*C.longitarsis* Kieffer

*C.longa* Chen & Johnson, sp. n.

*C.maura* Chen & Masner, sp. n.

*C.parvitas* Chen & Johnson, sp. n.

*C.pilus* Chen & Johnson, sp. n.

*C.plana* Chen & Johnson, sp. n.

*C.rara* Chen & Johnson, sp. n.

*C.robusta* Chen & Johnson, sp. n.

*C.rufithorax* Kieffer

*C.semicyanea* Ashmead

*C.semilutea* Chen & Johnson, sp. n.

*C.sparsa* Chen & Johnson, sp. n.

*C.tricarinata* Kieffer

### Key to females

**Table:** 

1	Basiconic sensilla on A7 absent (Figs 2, 6)	**2**
–	Basiconic sensilla on A7 present (Figs 4, 8)	**14**
2	Median mesoscutal carina absent (Figs 197, 227, 233, 236)	**3**
–	Median mesoscutal carina present (Figs 33, 39, 97, 109, 131, 137, 143, 149, 155, 173, 209, 237, 238)	**4**
3	Apex of T6 acute in dorsal view (Figure 199); horn on T1 absent (Figure 199); median metascutellar carina indistinguishable from surrounding rugae	***Chromoteleiarobusta* Chen & Johnson, sp. n.**
–	Apex of T6 rounded in dorsal view (Figure 235); horn on T1 present (Figure 235); median metascutellar carina distinct	***Chromoteleiatricarinata* Kieffer**
4	A12 with 1 basiconic sensillum	**5**
–	A12 with 2 basiconic sensilla	**10**
5	Posterior notaulus foveate (Figs 61, 67, 143, 149, 155)	**6**
–	Posterior notaulus smooth (Figs 39, 109, 137)	**8**
6	Hind basitarsus as long as remaining segments combined; dorsal metapleural area without setae (Figs 14, 59, 65, 153); ventral metapleural area smooth posteriorly (Figs 14, 59, 65, 153)	**7**
–	Hind basitarsus distinctly longer than remaining segments combined (Figure 27); dorsal metapleural area setose (Figure 15); ventral metapleural area rugose posteriorly (Figure 15)	***Chromoteleialongitarsis* Kieffer**
7	T1 with horn (Figs 61, 67); occipital carina complete (Figs 61, 67); A6 as long as wide (Figure 67)	***Chromoteleiaconnectens* Kieffer**
–	T1 without horn (Figure 157); occipital carina interrupted medially (Figure 155); A6 distinctly longer than wide (Figure 5)	***Chromoteleialonga* Chen & Johnson, sp. n.**
8	Apex of T6 rounded in dorsal view (Figs 41, 139); T1 with horn (Figs 41, 139)	**9**
–	Apex of T6 acute in dorsal view (Figure 111); T1 without horn (Figure 111)	***Chromoteleiafeng* Chen & Johnson, sp. n.**
9	Metasoma variably patterned in alternating orange-yellow and dark brown (Figure 41); mesoscutellum smooth medially, densely punctate laterally (Figure 39)	***Chromoteleiaalternata* Chen & Johnson, sp. n.**
–	Metasoma entirely black (Figure 139); mesoscutellum densely punctate rugose (Figure 137)	***Chromoteleialevitas* Chen & Johnson, sp. n.**
10	Dorsal metapleural area setose (Figs 129, 171)	**11**
–	Dorsal metapleural area without setae (Figs 31, 95, 207)	**12**
11	A6 distinctly longer than wide (Figure 130); occipital carina complete (Figure 131); hind basitarsus distinctly longer than remaining segments combined	***Chromoteleiaingens* Chen & Masner, sp. n.**
–	A6 as long as wide (Figure 170); occipital carina interrupted medially (Figure 173); hind basitarsus as long as remaining segments combined (Figure 170)	***Chromoteleiapilus* Chen & Johnson, sp. n.**
12	Mesosoma black (Figure 33); netrion densely punctate (Figure 31); mesoscutal midlobe densely punctate (Figure 33)	***Chromoteleiaaequalis* Chen & Johnson, sp. n.**
–	Mesosoma orange or variably orange to black (Figs 95, 97, 207); netrion longitudinally striate (Figs 95, 207); mesoscutal midlobe densely punctate rugose (Figs 97, 209)	**13**
13	Occiput rugose (Figure 97); area directly dorsal to the metapleural triangle with setae (Figure 95); hind coxa densely punctate (Figure 95); T6 at least 1.5× longer than wide (Figure 19)	***Chromoteleiadepilis* Chen & Johnson, sp. n.**
–	Occiput smooth (Figure 209); area directly dorsal to the metapleural triangle without setae (Figure 207); hind coxa largely smooth, with sparse fine punctures (Figure 207); T6 approximately as long as wide (Figure 211)	***Chromoteleiasemilutea* Chen & Johnson, sp. n.**
14	Apex of T6 acute in dorsal view (Figs 20, 81, 163)	**15**
–	Apex of T6 rounded in dorsal view (Figs 22, 57, 75, 87, 93, 105, 117, 169, 181, 187, 205, 217, 223)	**17**
15	Mesosoma variably orange to black (Figs 43, 77); netrion longitudinally striate (Figs 43, 77); notaulus foveate (Figs 45, 79)	**16**
–	Mesosoma black (Figs 159, 161); netrion rugose (Figure 159); notaulus smooth (Figure 161)	***Chromoteleiamaura* Chen & Masner, sp. n.**
16	Metascutellum with deeply incised apex, forming two spines laterally (Figure 45); postmarginal vein approximately as long as stigma vein (Figure 47); T1 without horn (Figure 45)	***Chromoteleiabidens* Chen & Masner, sp. n.**
–	Metascutellum trapezoidal with broad apex (Figure 79); postmarginal vein distinctly shorter than stigma vein (Figure 81); T1 with horn (Figure 79)	***Chromoteleiacuneus* Chen & Johnson, sp. n.**
17	Hind basitarsus as long as remaining segments combined	**18**
–	Hind basitarsus distinctly longer than remaining segments combined	**20**
18	Metasoma orange (Figure 105); mesoscutal midlobe with two rows of foveate grooves along median mesoscutal carina anteriorly, smooth at posterior margin (Figure 103); postmarginal vein distinctly longer than stigmal vein	***Chromoteleiadispar* Chen & Masner, sp. n.**
–	Metasoma black (Figs 169, 217); mesoscutal midlobe punctate rugose anteriorly, sparsely punctate at posterior margin (Figs 167, 215); postmarginal vein approximately as long as stigmal vein (Figs 169, 217)	**19**
19	A5 as wide as long (Figure 3); T1 without horn (Figure 169); T6 approximately as long as wide (Figure 169)	***Chromoteleiaparvitas* Chen & Johnson, sp. n.**
–	A5 distinctly longer than wide (Figure 213); T1 with horn (Figure 215); T6 at least 1.5× longer than wide (Figure 217)	***Chromoteleiasparsa* Chen & Johnson, sp. n.**
20	Postmarginal vein distinctly shorter than stigmal vein (Figs 49, 120, 127, 187)	**21**
–	Postmarginal vein as long as or distinctly longer than stigmal vein (Figs 75, 87, 117, 181, 205)	**23**
21	Frons directly above interantennal process punctate rugose (Figure 56); dorsal frons with granulate microsculpture (Figure 56); netrion punctate rugose anteriorly, smooth posteriorly (Figure 53)	***Chromoteleiacongoana* (Risbec)**
–	Frons directly above interantennal process foveolate (Figs 121, 126, 186); dorsal frons without granulate microsculpture (Figs 121, 126, 186); netrion transversely striate (Figs 123, 183)	**22**
22	Dorsal metapleural area setose (Figure 123); mesoscutal midlobe sparsely punctate anteriorly (Figure 125); T6 sinuate in lateral view	***Chromoteleiafuscicornis* Kieffer**
–	Dorsal metapleural area without setae (Figure 183); mesoscutal midlobe punctate rugose anteriorly (Figure 185); T6 flat in lateral view	***Chromoteleiarara* Chen & Johnson, sp. n.**
23	Head metallic blue (Figs 203, 204); postmarginal vein distinctly longer than stigma vein (Figure 205); frons without central keel (Figure 204)	***Chromoteleiasemicyanea* Ashmead**
–	Head black (Figs 74, 116, 180); postmarginal vein approximately as long as stigma vein (Figs 75, 87, 117); frons with central keel (Figs 74, 86, 116, 180)	**24**
24	Mesoscutal midlobe smooth posteriorly (Figure 179); T2–T3 with a narrow smooth strip medially (Figure 181)	***Chromoteleiaplana* Chen & Johnson, sp. n.**
–	Mesoscutal midlobe sparsely punctate posteriorly (Figs 73, 85, 115); T2–T3 without a narrow smooth strip medially (Figs 75, 87, 117)	**25**
25	Notaulus foveate (Figs 73, 85); mesepisternum below femoral depression without striae (Figs 71, 83)	**26**
–	Notaulus smooth (Figure 115); mesepisternum below femoral depression with striae (Figure 113)	***Chromoteleiafossa* Chen & Johnson, sp. n.**
26	Netrion transversely striae (Figure 71); T6 at least 1.5× longer than wide (Figure 75)	***Chromoteleiacopiosa* Chen & Johnson, sp. n.**
–	Netrion rugose (Figure 83); T6 approximately as long as wide (Figure 87)	***Chromoteleiacurta* Chen & Johnson, sp. n.**

### Key to males (unknown for *C.maura, C.plana, C.fossa, C.parvitas*)

**Table:** 

1	Posterior margin of T7 without lateral spines or rounded projections (Figure 24)	**2**
–	Posterior margin of T7 with lateral spines (Figure 26) or rounded projections (Figure 25)	**14**
2	Hind basitarsus as long as remaining segments combined (Figure 28)	**3**
–	Hind basitarsus distinctly longer than remaining segments combined (Figure 27)	**9**
3	Dorsal metapleural area setose (Figs 171, 231)	**4**
–	Dorsal metapleural area without setae (Figs 37, 95, 107, 195, 207)	**5**
4	Median mesoscutal carina present anteriorly (Figure 173); ventral metapleural area rugose (Figure 171)	***Chromoteleiapilus* Chen & Johnson, sp. n.**
–	Median mesoscutal carina absent (Figs 227, 233); ventral metapleural area obliquely striate posteriorly (Figure 231)	***Chromoteleiatricarinata* Kieffer**
5	Area directly dorsal to the metapleural triangle with setae (Figure 95)	***Chromoteleiadepilis* Chen & Johnson, sp. n.**
–	Area directly dorsal to the metapleural triangle without setae (Figure 37, 107, 195, 207)	**6**
6	Frons with central keel developed only in ventral portion of frons (Figs 40, 110)	**7**
–	Frons with central keel complete, extending dorsally to median ocellus (Figs 198, 210)	**8**
7	Ventral metapleural area obliquely striate posteriorly (Figure 37); metasoma dark brown to black (Figure 41)	***Chromoteleiaalternata* Chen & Johnson, sp. n.**
–	Ventral metapleural area smooth posteriorly (Figure 107); metasoma entirely black (Figure 111)	***Chromoteleiafeng* Chen & Johnson, sp. n.**
8	Metasoma entirely black (Figure 199); median mesoscutal carina absent (Figure 197)	***Chromoteleiarobusta* Chen & Johnson, sp. n.**
–	Metasoma with T1–T3 orange to dark brown (Figure 211); median mesoscutal carina present (Figure 209)	***Chromoteleiasemilutea* Chen & Johnson, sp. n.**
9	Dorsal A1 striate	**10**
–	Dorsal A1 smooth or punctate	**11**
10	Mesosoma entirely black (Figure 33); netrion densely punctate (Figure 31); dorsal metapleural area without setae (Figure 31)	***Chromoteleiaaequalis* Chen & Johnson, sp. n.**
–	Mesosoma variably orange and black (Figure 147); netrion rugose (Figure 147); dorsal metapleural area setose (Figure 15)	***Chromoteleialongitarsis* Kieffer**
11	Head black (Figs 74, 86, 186); frons with central keel (Figs 74, 86, 186); mesopleural carina present (Figs 71, 83, 183)	**12**
–	Head metallic blue (Figure 204); frons without central keel (Figure 204); mesopleural carina absent	***Chromoteleiasemicyanea* Ashmead**
12	Occipital carina interrupted medially (Figs 73, 85); postmarginal vein approximately as long as stigma vein (Figs 75, 87)	**13**
–	Occipital carina complete (Figure 185); postmarginal vein distinctly shorter than stigma vein (Figure 187)	***Chromoteleiarara* Chen & Johnson, sp. n.**
13	Netrion transversely striate (Figure 71); epicoxal lobe posterior of propleural epicoxal sulcus smooth	***Chromoteleiacopiosa* Chen & Johnson, sp. n.**
–	Netrion rugose (Figure 83); epicoxal lobe posterior of propleural epicoxal sulcus densely punctate	***Chromoteleiacurta* Chen & Johnson, sp. n.**
14	Dorsal metapleural area setose (Figs 43, 123, 129)	**15**
–	Dorsal metapleural area without setae (Figs 14, 53, 77, 101, 135, 153, 213, 219)	**17**
15	Metascutellum with deeply incised apex, forming two spines laterally (Figure 45); apex of T7 bispinose (Figure 26)	***Chromoteleiabidens* Chen & Masner, sp. n.**
–	Metascutellum trapezoidal with broad posterior margin (Figs 119, 125, 131); apex of T7 emarginate between rounded projections (Figure 25)	**16**
16	Postmarginal vein distinctly shorter than stigma vein (Figure 127); A6 as long as wide	***Chromoteleiafuscicornis* Kieffer**
–	Postmarginal vein distinctly longer than stigma vein (Figure 133); A6 approximately 2.0× longer than wide (Figure 9)	***Chromoteleiaingens* Chen & Masner, sp. n.**
17	Dorsal A1 striate	**18**
–	Dorsal A1 smooth or punctate	**20**
18	Notaulus foveate (Figs 61, 67, 155); postmarginal vein distinctly longer than stigmal vein (Figs 63, 157)	**19**
–	Notaulus mostly smooth (Figure 137); postmarginal vein approximately as long as stigmal vein (Figure 139)	***Chromoteleialevitas* Chen & Johnson, sp. n.**
19	Occipital carina complete (Figure 61); lateral occiput rugose (Figure 61); gena punctate rugose ventrally (Figure 59)	***Chromoteleiaconnectens* Kieffer**
–	Occipital carina interrupted medially (Figure 155); occiput smooth (Figure 155); gena narrowly smooth ventrally (Figure 153)	***Chromoteleialonga* Chen & Johnson, sp. n.**
20	Frons without central keel (Figure 104); mesosoma black (Figure 103); postmarginal vein distinctly longer than stigmal vein (Figure 105)	***Chromoteleiadispar* Chen & Masner, sp. n.**
–	Frons with central keel (Figs 56, 80, 86); mesosoma variably orange to black (Figs 55, 77, 83); postmarginal vein distinctly shorter than stigmal vein (Figs 49, 81, 87)	**21**
21	Mesoscutellum sparsely punctate (Figure 55); T2 without transverse sulcus (Figure 57); hind basitarsus distinctly longer than remaining segments combined	***Chromoteleiacongoana* (Risbec)**
–	Mesoscutellum smooth medially, densely punctate laterally (Figs 79, 85); T2 with transverse sulcus (Figs 81, 87); hind basitarsus as long as remaining segments combined	**22**
22	Lateral lobe of mesoscutum punctate rugose (Figure 79); netrion transversely striate (Figure 77); hind coxa densely punctate (Figure 77)	***Chromoteleiacuneus* Chen & Johnson, sp. n.**
–	Lateral lobe of mesoscutum sparsely punctate (Figure 85); netrion rugose (Figure 83); hind coxa largely smooth, with sparse fine punctures	***Chromoteleiasparsa* Chen & Johnson, sp. n.**

**Figures 7–10.**
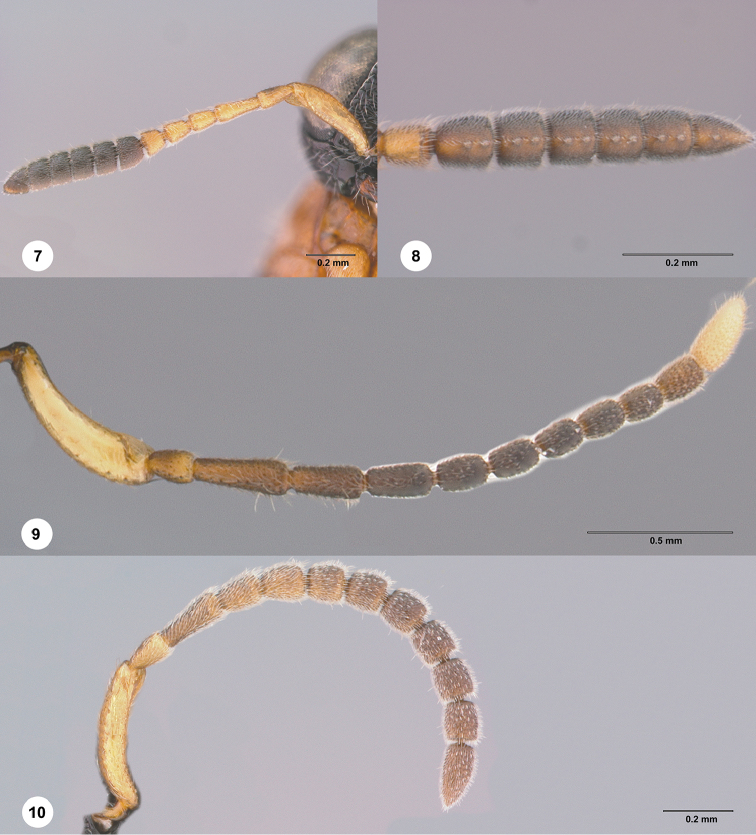
**7–8***Chromoteleiarara* sp. n., female, holotype (OSUC577495). **7** Antenna **8** Antennal clava, ventral view **9***Chromoteleiaingens* sp. n., paratype, male (OSUC583458) Antenna **10***Chromoteleiabidens* sp. n., paratype, male (OSUC185675) Antenna.

**Figures 11–13.**
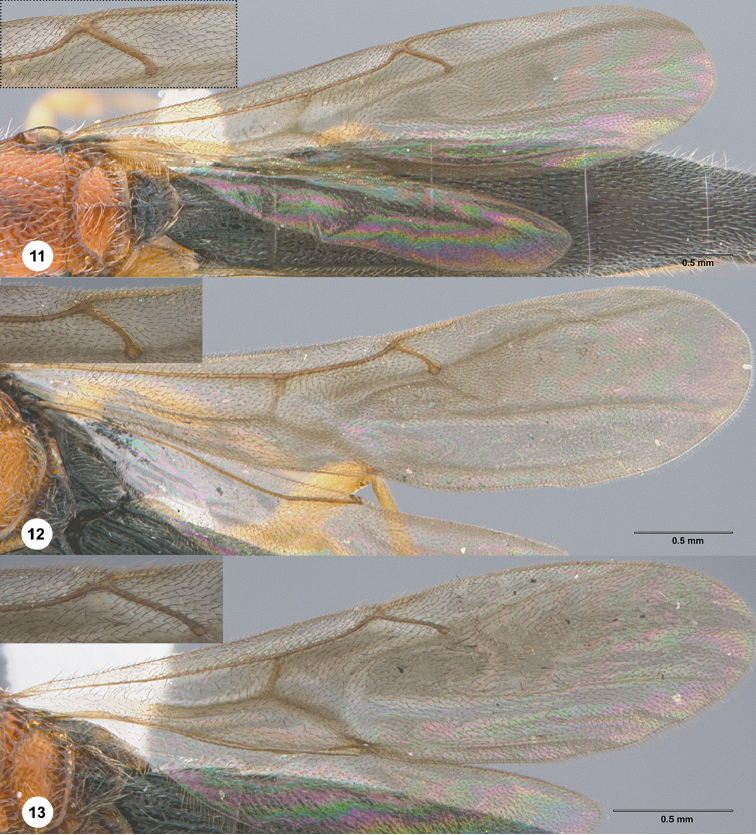
Wings **11***Chromoteleialongitarsis* Kieffer, female (OSUC584896). **12***Chromoteleiarobusta* sp. n., female, paratype (OSUC577466) **13***Chromoteleiacuneus* sp. n., female, holotype (OSUC585001).

**Figures 14–15.**
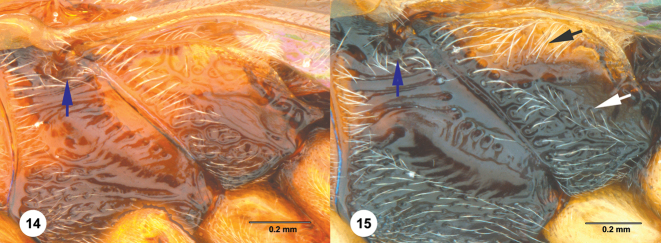
Mesopleuron and metapleuron **14***Chromoteleiaconnectens* Kieffer, female, holotype (CAS TYPE9618) **15***Chromoteleialongitarsis* Kieffer, female (OSUC584896).

**Figures 16–21.**
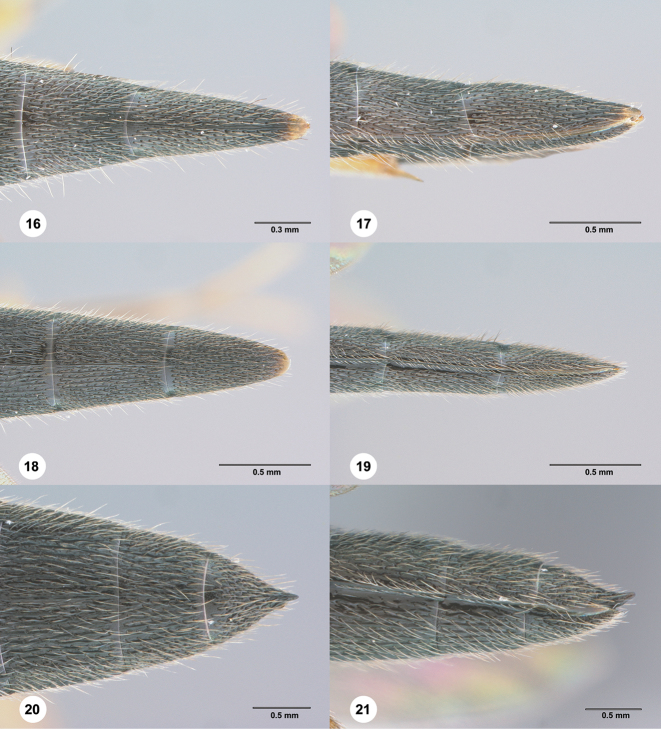
**16–17***Chromoteleialongitarsis* Kieffer, female (OSUC584896). **16** Apical metasoma, dorsal view **17** Apical metasoma, lateral view **18–19***Chromoteleiadepilis* sp. n., female, holotype (OSUC577436)) **18** Apical metasoma, dorsal view **19** Apical metasoma, lateral view **20–21***Chromoteleiabidens* sp. n., female, holotype (OSUC577455) **20** Apical metasoma, dorsal view **21** Apical metasoma, lateral view.

**Figures 22–29.**
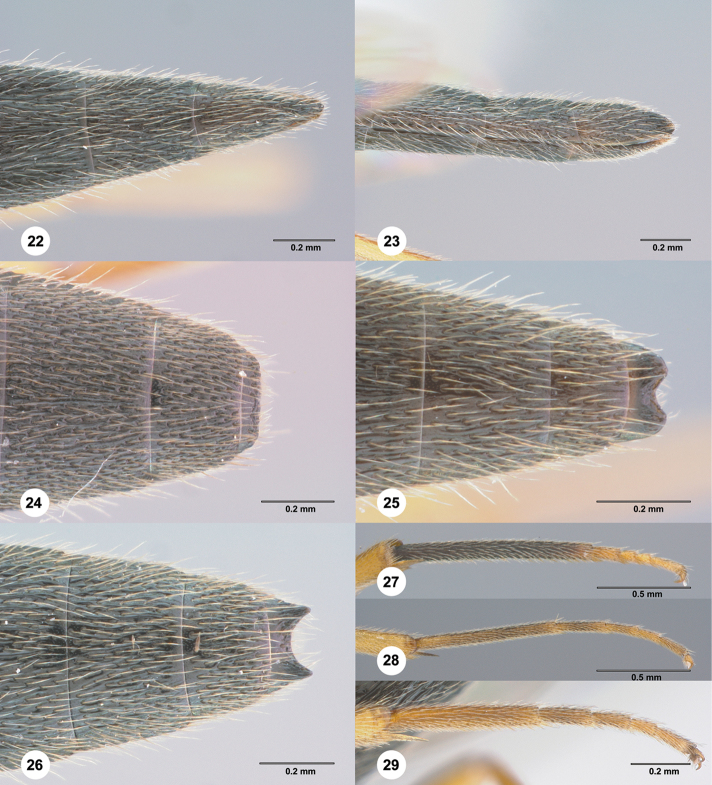
**22–23***Chromoteleiafuscicornis* Kieffer, female (OSUC584322). **22** Apical metasoma, dorsal view **23** Apical metasoma, lateral view **24***Chromoteleialongitarsis* Kieffer, male, holotype (CAS TYPE9682), apical metasoma, dorsal view **25***Chromoteleiafuscicornis* Kieffer, male (OSUC586417), apical metasoma, dorsal view **26***Chromoteleiabidens* sp. n., male, paratype (OSUC577455), apical metasoma, dorsal view Apical **27***Chromoteleialongitarsis* Kieffer, female (OSUC584896), hind tarsus **28***Chromoteleiadepilis* sp. n., female, paratype (OSUC577436), hind tarsus **29***Chromoteleiabidens* sp. n., female, paratype (OSUC577455), hind tarsus.

### Chromoteleia aequalis

Taxon classificationAnimaliaHymenopteraScelionidae

Chen & Johnson sp. n.

http://zoobank.org/A91D1FE4-DEF0-4D3F-BC48-3F29A67FC42A

http://bioguid.osu.edu/xbiod_concepts/452211

Figures 30–35

#### Description.

Body length of female: 6.88–7.27 mm (n = 3). Body length of male: 5.90–6.20 mm (n = 3). Color of A1: yellow to orange. A6 in female: distinctly longer than wide. A5 in female: distinctly longer than wide. A6 in male: approximately 2.0× longer than wide. Number of basiconic sensilla on A7: 0. Number of basiconic sensilla on A12: 2. Sculpture of dorsal A1: striate. Color of head: black. Sculpture of frons directly above interantennal process: transversely striate to rugose. Central keel: present, interrupted medially. Ventral margin of clypeus: pointed. Granulate microsculpture of dorsal frons: absent. Occipital carina: interrupted medially. Granulate microsculpture of vertex: absent. Sculpture of occiput: smooth. Sculpture of gena: dorsoventrally strigose.

Color of mesosoma: black. Sculpture of epicoxal lobe posterior of propleural epicoxal sulcus: densely punctate. Sculpture of lateral pronotal area above pronotal cervical sulcus: smooth dorsally, rugose ventrally. Sculpture of netrion: densely punctate. Microsculpture of mesoscutum: granulate. Macrosculpture of mesoscutal midlobe: densely punctate. Macrosculpture of lateral lobe of mesoscutum: densely punctate. Sculpture of notaulus: foveate. Notaular foveae: discrete. Median mesoscutal carina: present anteriorly, not extending to posterior margin of mesoscutum. Mesoscutellum in lateral view: flat. Sculpture of mesoscutellum: densely punctate rugose. Shape of metascutellum: trapezoidal with broad posterior margin. Median metascutellar carina: absent or indistinguishable from sculpture. Sculpture of metascutellum: areolate. Sculpture of lateral propodeal area: rugose. Mesopleural carina: absent. Sculpture of mesepisternum below femoral depression: smooth directly below femoral depression, otherwise densely punctate. Sculpture of dorsal metapleural area: rugose. Setation of dorsal metapleural area: absent. Setation of area directly dorsal to the metapleural triangle: present. Sculpture of ventral metapleural area: rugose anteriorly, smooth posteriorly. Color of legs: pale yellow with tarsi dark brown to black. Length of hind basitarsus: distinctly longer than remaining segments combined. Sculpture of hind coxa: densely punctate.

Length of postmarginal vein: distinctly longer than stigmal vein.

Color of metasoma in female: black. Color of metasoma in male: black. Horn on T1 in female: present. Striae of posterior margin of T1 in female: dense. Striae of T1 in male: dense. Transverse sulcus on T2: present. Sculpture of T2: longitudinally punctate rugose. Sculpture of T6 in female: densely longitudinally striate, with fine punctures in interstices. Length of T6 in female: at least 1.5× longer than wide. Shape of T6 in female in lateral view: flat. Apical spine on female T6: absent. Sculpture of T6 in male: densely longitudinally striate with fine punctures in interstices. Sculpture of T7 in male: smooth to coriaceous. Posterior margin of T7 in male: straight. Sculpture of medial S2: densely punctate to punctate rugose.

**Figures 30–35.**
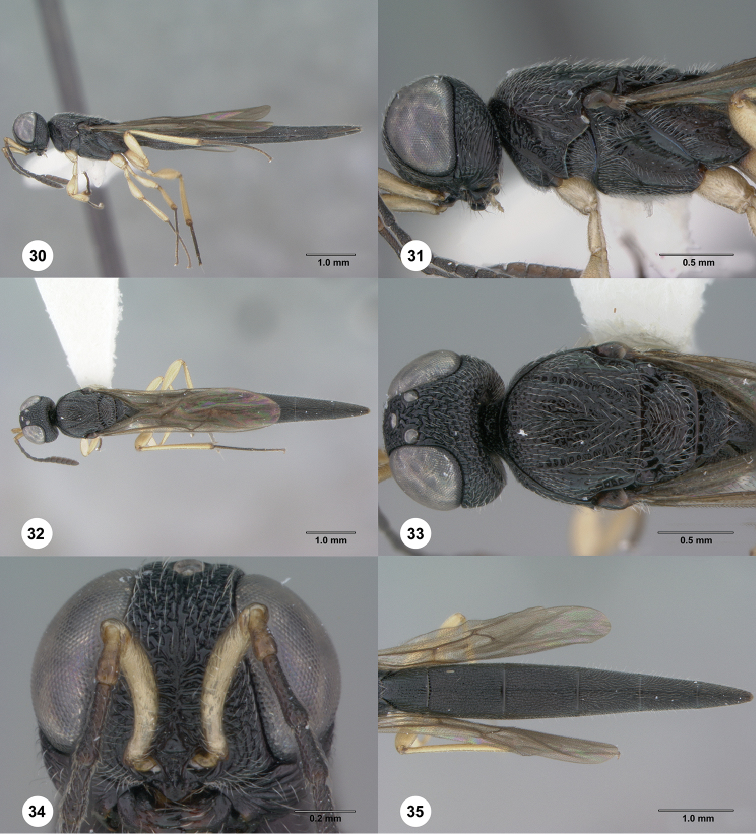
*Chromoteleiaaequalis* sp. n., female, holotype (OSUC221942). **30** Lateral habitus **31** Head and mesosoma, lateral view **32** Dorsal habitus **33** Head and mesosoma, dorsal view **34** Head, anterior view **35** Metasoma, dorsal view.

#### Diagnosis.

This species is easily recognized by its entirely black body both in female and male, densely punctate mesoscutum, and abbreviated median mesoscutal carina.

#### Etymology.

The epithet is inspired by the Latin word for equal, in reference to the black body color shared by males and females, and is intended to be treated as an adjective.

#### Link to distribution map.

[http://hol.osu.edu/map-large.html?id=452211]

#### Material examined.

Holotype, female: **DOMINICA**: Saint Paul Parish, Springfield, 94/017, Archbold Tropical Research Center (ATRC), 27.V.1994, J. B. Woolley, OSUC221942 (deposited in TAMU). *Paratypes*: (2 females, 3 males) **DOMINICA**: 1 female, 3 males, OSUC584722–584725 (TAMU). **GUYANA**: 1 female, OSUC215798 (BPBM).

### Chromoteleia alternata

Taxon classificationAnimaliaHymenopteraScelionidae

Chen & Johnson sp. n.

http://zoobank.org/5BA6E119-AF80-47BA-9BF6-A28039A09E23

http://bioguid.osu.edu/xbiod_concepts/318205

Figures 36–41

#### Description.

Body length of female: 5.38–5.90 mm (n = 10). Body length of male: 4.40–4.93 mm (n = 20). Color of A1: yellow to orange. A6 in female: as wide as long. A5 in female: distinctly longer than wide. A6 in male: approximately 2.0× longer than wide. Number of basiconic sensilla on A7: 0. Number of basiconic sensilla on A12: 1. Sculpture of dorsal A1: striate. Color of head: black. Sculpture of frons directly above interantennal process: transversely striate to rugose. Central keel: present only in ventral portion of frons. Ventral margin of clypeus: pointed. Granulate microsculpture of dorsal frons: present. Occipital carina: complete. Granulate microsculpture of vertex: absent. Sculpture of occiput: smooth. Sculpture of gena: dorsoventrally strigose.

Color of mesosoma: orange. Sculpture of epicoxal lobe posterior of propleural epicoxal sulcus: sparsely punctate. Sculpture of lateral pronotal area above pronotal cervical sulcus: rugulose. Sculpture of netrion: punctate rugose anteriorly, smooth posteriorly. Microsculpture of mesoscutum: granulate. Macrosculpture of mesoscutal midlobe: punctate rugose anteriorly, sparsely punctate posteriorly. Macrosculpture of lateral lobe of mesoscutum: sparsely punctate. Sculpture of notaulus: smooth. Median mesoscutal carina: present anteriorly, not extending to posterior margin of mesoscutum. Mesoscutellum in lateral view: convex. Sculpture of mesoscutellum: smooth medially, densely punctate laterally. Shape of metascutellum: trapezoidal with broad posterior margin. Median metascutellar carina: absent or indistinguishable from sculpture. Sculpture of metascutellum: rugose. Mesopleural carina: present. Sculpture of mesepisternum below femoral depression: rugose anteriorly, sparsely punctate posteriorly. Sculpture of dorsal metapleural area: rugose; smooth. Setation of dorsal metapleural area: absent. Setation of area directly dorsal to the metapleural triangle: absent. Sculpture of ventral metapleural area: rugose anteriorly, obliquely striate posteriorly. Color of legs: orange yellow throughout. Length of hind basitarsus: distinctly longer than remaining segments combined. Sculpture of hind coxa: largely smooth, with sparse fine punctures.

Length of postmarginal vein: approximately as long as stigmal vein.

Color of metasoma in female: variably patterned in alternating orange yellow and dark brown. Color of metasoma in male: dark brown to black. Horn on T1 in female: present. Striae of posterior margin of T1 in female: dense. Striae of T1 in male: dense. Transverse sulcus on T2: present. Sculpture of T2: densely longitudinally striate, punctate rugulose in interstices. Sculpture of T6 in female: longitudinally punctate rugose. Length of T6 in female: at least 1.5× longer than wide. Shape of T6 in female in lateral view: flat. Apical spine on female T6: absent. Sculpture of T6 in male: densely punctate. Sculpture of T7 in male: densely punctate. Posterior margin of T7 in male: straight. Sculpture of medial S2: densely longitudinally striate with fine punctures in interstices.

**Figures 36–41.**
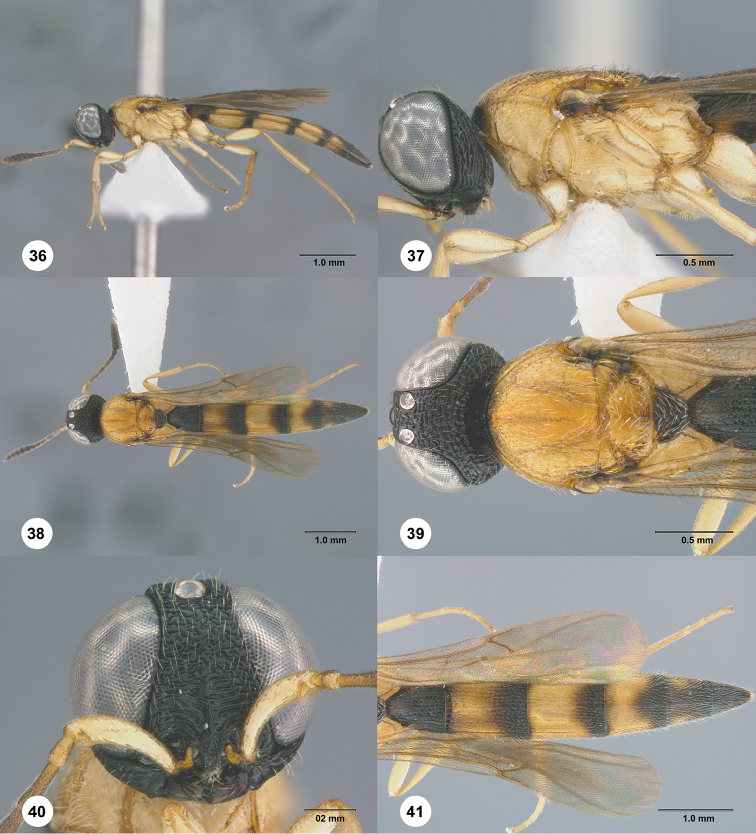
*Chromoteleiaalternata* sp. n., female, holotype (OSUC136573). **36** Lateral habitus **37** Head and mesosoma, lateral view **38** Dorsal habitus **39** Head and mesosoma, dorsal view **40** Head, anterior view **41** Metasoma, dorsal view.

#### Diagnosis.

This species can be easily distinguished from other *Chromoteleia* species by the following combination of characters: female metasoma variably patterned in alternating orange yellow and dark brown, central keel present only in ventral portion of frons, and notaulus smooth.

#### Etymology.

The epithet is inspired by the Latin word for alternate, in reference to the variably patterned in alternating orange yellow and yellow on metasoma, and is intended to be treated as a noun.

#### Link to distribution map.

[http://hol.osu.edu/map-large.html?id=452211]

#### Material examined.

Holotype, female: **BRAZIL**: ES, Santa Teresa, 660 m, 19°58'19"S, 40°32'00"W, Santa Lucia Biological Station, 30.V–2.VI.1997, yellow pan trap, W. A. Hoffmann & R. Ribeiro, OSUC136573 (deposited in OSUC). *Paratypes*: **BRAZIL**: 9 females, 56 males, OSUC149637, 149645, 202506–202508 (AEIC); OSUC586304–586305, 586307, 586309, 586320–586321, 586323–586326, 586575–586576, 586582–586584, 586588–586589, 586591–586595, 586597, 586599, 586601–586602 (CNCI); OSUC199607 (FSCA); OSUC127206, 128954, 136099, 137616, 148096, 149994, 233157, 233159–233160, 233198, 233332, 235222, 254872–254874, 79997 (MZSP); OSUC122256, 126972, 138446, 147578, 148058, 148097, 149921, 149995, 150331, 233158, 233161, 233197, 233199, 233331, 246487, 254871, 322558 (OSUC).

### Chromoteleia bidens

Taxon classificationAnimaliaHymenopteraScelionidae

Chen & Masner sp. n.

http://zoobank.org/9FEF89BB-7DA6-461B-BA7B-3B97EE128F9B

http://bioguid.osu.edu/xbiod_concepts/318198

Figures 10 , 20–21 , 26 , 29 , 42–47

#### Description.

Body length of female: 4.20–4.80 mm (n = 16). Body length of male: 4.30–4.67 mm (n = 17). Color of A1: yellow to orange. A6 in female: distinctly wider than long. A5 in female: distinctly longer than wide. A6 in male: as long as wide. Number of basiconic sensilla on A7: 1. Number of basiconic sensilla on A12: 1. Sculpture of dorsal A1: punctate. Color of head: black. Sculpture of frons directly above interantennal process: transversely striate to rugose. Central keel: present, interrupted medially. Ventral margin of clypeus: straight. Granulate microsculpture of dorsal frons: present. Occipital carina: complete. Granulate microsculpture of vertex: present. Sculpture of occiput: smooth. Sculpture of gena: coarsely punctate rugose.

Color of mesosoma: variably orange to black. Sculpture of epicoxal lobe posterior of propleural epicoxal sulcus: sparsely punctate. Sculpture of lateral pronotal area above pronotal cervical sulcus: smooth throughout. Sculpture of netrion: transversely striate. Microsculpture of mesoscutum: granulate. Macrosculpture of mesoscutal midlobe: sparsely punctate. Macrosculpture of lateral lobe of mesoscutum: sparsely punctate. Sculpture of notaulus: foveate. Notaular foveae: interconnected. Median mesoscutal carina: present anteriorly, not extending to posterior margin of mesoscutum. Mesoscutellum in lateral view: convex. Sculpture of mesoscutellum: granulate. Shape of metascutellum: elongate trapezoidal but with deeply incised apex, forming two spines laterally. Median metascutellar carina: absent or indistinguishable from sculpture. Sculpture of metascutellum: rugose. Sculpture of lateral propodeal area: rugose. Mesopleural carina: absent. Sculpture of mesepisternum below femoral depression: punctate rugose. Sculpture of dorsal metapleural area: rugose; obliquely striate. Setation of dorsal metapleural area: present. Setation of area directly dorsal to the metapleural triangle: present. Sculpture of ventral metapleural area: rugose throughout. Color of legs: orange yellow to brown, with tarsi darker. Length of hind basitarsus: about as long as remaining segments combined. Sculpture of hind coxa: densely punctate.

Length of postmarginal vein: approximately as long as stigmal vein.

Color of metasoma in female: black. Color of metasoma in male: black. Horn on T1 in female: absent. Striae of posterior margin of T1 in female: sparse. Striae of T1 in male: sparse. Transverse sulcus on T2: present. Sculpture of T2: densely longitudinally striate, punctate rugulose in interstices. Sculpture of T6 in female: longitudinally punctate rugose. Length of T6 in female: approximately as long as wide. Shape of T6 in female in lateral view: sinuate. Apical spine on female T6: present. Sculpture of T6 in male: densely punctate. Sculpture of T7 in male: smooth anteriorly, rugulose posteriorly. Posterior margin of T7 in male: bispinose. Sculpture of medial S2: densely longitudinally striate with fine punctures in interstices.

**Figures 42–47.**
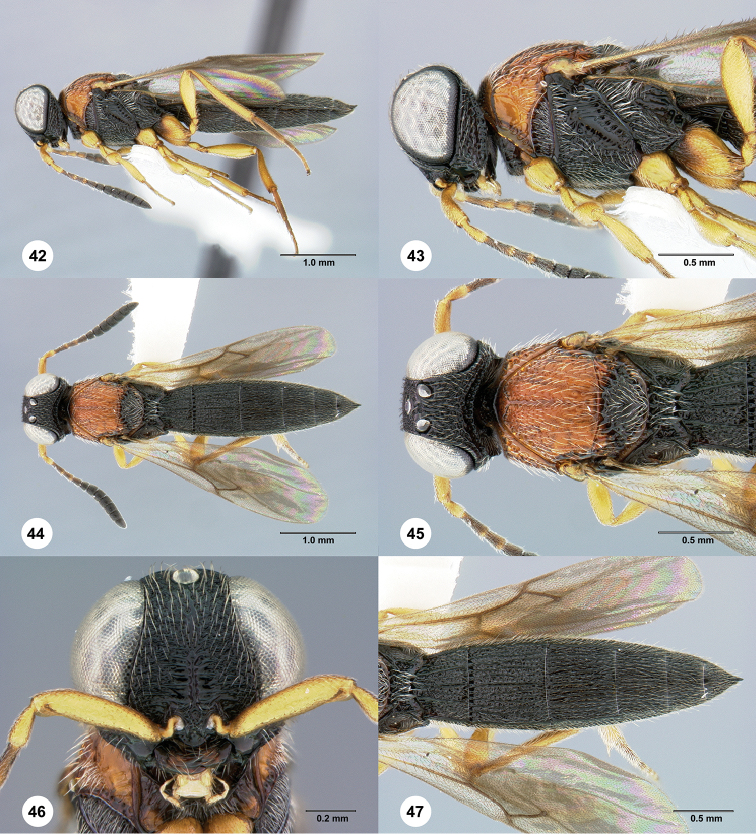
*Chromoteleiabidens* sp. n., female, holotype (OSUC577455). **42** Lateral habitus **43** Head and mesosoma, lateral view **44** Dorsal habitus **45** Head and mesosoma, dorsal view **46** Head, anterior view **47** Metasoma, dorsal view.

#### Diagnosis.

This species is easily recognized by its bispinose metascutellum, a character not found in any other species of *Chromoteleia*.

#### Etymology.

The epithet is inspired by the Latin word for two-toothed, in reference to the bispinose metascutellum, and is intended to be treated as a noun.

#### Link to distribution map.

[http://hol.osu.edu/map-large.html?id=318198]

#### Material examined.

Holotype, female: **BRAZIL**: RO, 62 km S Ariquemes, Rancho Grande Farm, 12.XI–22.XI.1991, E. M. Fisher, OSUC577455 (deposited in CNCI). *Paratypes*: (15 females, 15 males) **BOLIVIA**: 1 female, OSUC577440 (CNCI). **BRAZIL**: 8 females, 12 males, OSUC577452–577453, 577456–577458, 584325 (CNCI); OSUC204986, 204991 (UCDC); OSUC185665–185676 (USNM). **ECUADOR**: 1 female, OSUC577454 (CNCI). **FRENCH GUIANA**: 2 females, OSUC586452, 586827 (CNCI). **GUYANA**: 2 females, OSUC232995 (BPBM); OSUC577451 (CNCI). **PERU**: 1 female, 3 males, OSUC577459, 584310, 586806 (CNCI); OSUC323993 (OSUC).

### Chromoteleia congoana

Taxon classificationAnimaliaHymenopteraScelionidae

(Risbec)

http://zoobank.org/381B96B2-0567-4A2C-B2C2-F8E69D0E32A0

http://bioguid.osu.edu/xbiod_concepts/4211

Figures 48–51 , 52–57

Oxyscelio congoana Risbec, 1950: 613 (original description).
Chromoteleia congoana (Risbec): Masner, 1976: 25 (description, generic transfer, type information); Johnson, 1992: 364 (cataloged, type information).

#### Description.

Body length of female: 4.38–5.66 mm (n = 20). Body length of male: 4.10–4.78 mm (n = 4). Color of A1: yellow to orange. A6 in female: as wide as long. A5 in female: distinctly longer than wide. A6 in male: approximately 2.0× longer than wide. Number of basiconic sensilla on A7: 1. Number of basiconic sensilla on A12: 1. Sculpture of dorsal A1: punctate; smooth. Color of head: black. Sculpture of frons directly above interantennal process: punctate rugose. Central keel: complete, extending from interantennal process to median ocellus. Ventral margin of clypeus: pointed. Granulate microsculpture of dorsal frons: present. Occipital carina: complete. Granulate microsculpture of vertex: present. Sculpture of occiput: rugose. Sculpture of gena: punctate rugose dorsally and ventrally, strigose medially.

Color of mesosoma: variably orange to black. Sculpture of epicoxal lobe posterior of propleural epicoxal sulcus: sparsely punctate. Sculpture of lateral pronotal area above pronotal cervical sulcus: smooth dorsally, rugose ventrally. Sculpture of netrion: punctate rugose anteriorly, smooth posteriorly. Microsculpture of mesoscutum: coriaceous. Macrosculpture of mesoscutal midlobe: punctate rugose anteriorly, sparsely punctate posteriorly. Macrosculpture of lateral lobe of mesoscutum: sparsely punctate. Sculpture of notaulus: foveate. Notaular foveae: interconnected. Median mesoscutal carina: present anteriorly, not extending to posterior margin of mesoscutum. Mesoscutellum in lateral view: convex. Sculpture of mesoscutellum: sparsely punctate. Shape of metascutellum: trapezoidal with broad posterior margin. Median metascutellar carina: absent or indistinguishable from sculpture. Sculpture of metascutellum: rugose. Mesopleural carina: present. Sculpture of mesepisternum below femoral depression: punctate throughout. Sculpture of dorsal metapleural area: rugose. Setation of dorsal metapleural area: absent. Setation of area directly dorsal to the metapleural triangle: absent. Sculpture of ventral metapleural area: rugose throughout. Color of legs: orange yellow throughout. Length of hind basitarsus: distinctly longer than remaining segments combined. Sculpture of hind coxa: densely punctate.

Length of postmarginal vein: distinctly shorter than stigmal vein.

Color of metasoma in female: dark brown. Color of metasoma in male: dark brown to black. Horn on T1 in female: present. Striae of posterior margin of T1 in female: dense. Striae of T1 in male: sparse. Transverse sulcus on T2: absent. Sculpture of T2: densely longitudinally striate, punctate rugulose in interstices. Sculpture of T6 in female: densely longitudinally striate, with fine punctures in interstices. Length of T6 in female: at least 1.5× longer than wide. Shape of T6 in female in lateral view: flat. Apical spine on female T6: absent. Sculpture of T6 in male: densely longitudinally striate with fine punctures in interstices. Sculpture of T7 in male: smooth anteriorly, rugulose posteriorly. Posterior margin of T7 in male: emarginate medially between rounded projections. Sculpture of medial S2: densely longitudinally striate with fine punctures in interstices.

**Figures 48–51.**
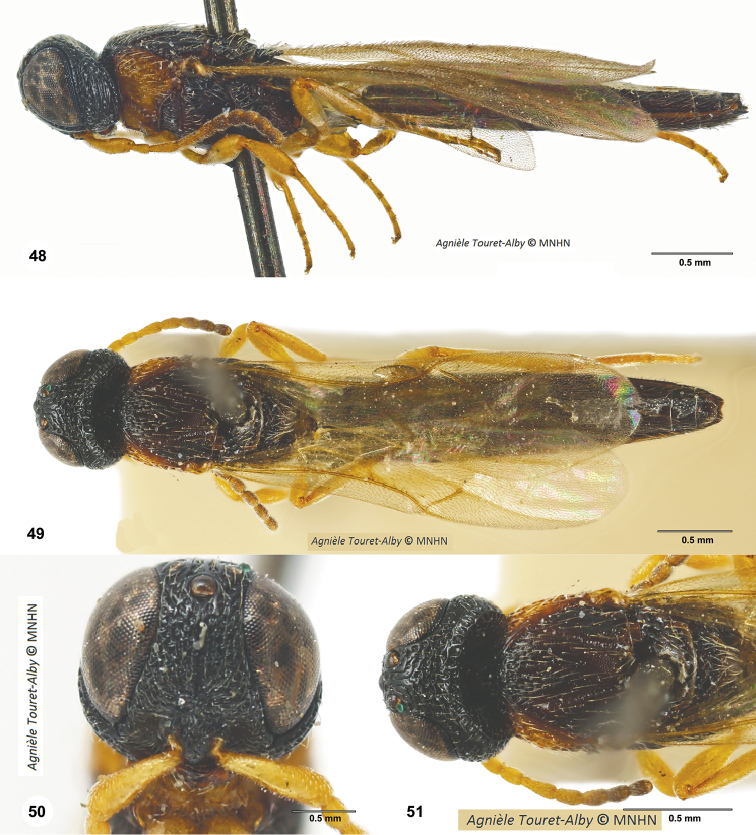
*Oxysceliocongoana* Risbec, male, holotype (EY11376). **48** Lateral habitus **49** Dorsal habitus **50** Head, anterior view **51** Head and mesosoma, dorsal view.

**Figures 52–57.**
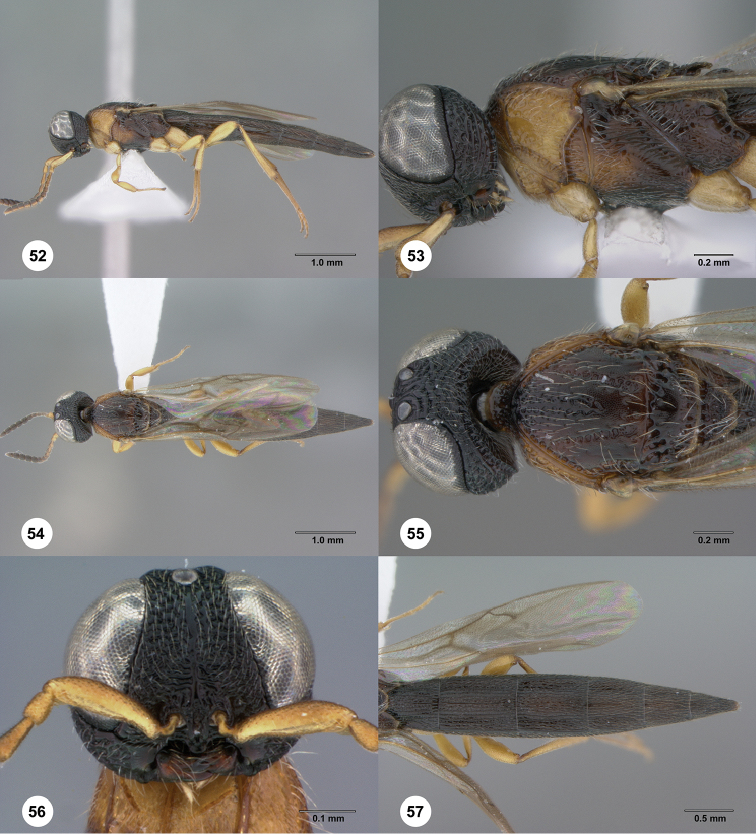
*Chromoteleiacongoana* (Risbec), female (OSUC207829). **52** Lateral habitus **53** Head and mesosoma, lateral view **54** Dorsal habitus **55** Head and mesosoma, dorsal view **56** Head, anterior view **57** Metasoma, dorsal view.

#### Diagnosis.

This species can be distinguished from other *Chromoteleia* species by the following combination of characters: median mesoscutal carina only present anteriorly, mesosoma variably orange to black, postmarginal vein distinctly shorter than stigma vein, T2 without transverse sulcus, male T7 with posterior margin deeply emarginated between rounded projections.

#### Link to distribution map.

[http://hol.osu.edu/map-large.html?id=4211]

#### Material examined.

Holotype, male, *O.congoana*: **GABON**: Estuaire Prov., Libreville, 1901, C. Chalot, EY11376 (deposited in MNHN). Other material: (35 females, 4 males) **CAMEROON**: 3 females, 1 male, OSUC584461–584464 (CNCI). **CENTRAL AFRICAN REPUBLIC**: 9 females, 1 male, OSUC226055–226056, 254563, 320836–320837 (OSUC); OSUC176093, 180933, 207829, 223628, 267420 (SAMC). **DEMOCRATIC REPUBLIC OF THE CONGO**: 1 male, OSUC584460 (CNCI). **GABON**: 1 female, 1 male, OSUC584457–584458 (CNCI). **GHANA**: 1 female, OSUC412136 (OSUC). **GUINEA**: 1 female, OSUC584459 (CNCI). **NIGERIA**: 20 females, OSUC584452–584456, 584465–584479 (CNCI).

#### Comments.

This species is only found in Afrotropical region.

### Chromoteleia connectens

Taxon classificationAnimaliaHymenopteraScelionidae

Kieffer

http://zoobank.org/7409F8CA-0594-4232-8481-FCC20CDC290E

http://bioguid.osu.edu/xbiod_concepts/4212

Figures 14 , 58–63 , 64–69 , 237

Chromoteleia (Oxyscelio) connectens Kieffer, 1910a: 313 (original description, keyed).
Oxyscelio (Oxyscelio) connectens (Kieffer): Kieffer, 1910b: 69 (generic transfer).
Oxyscelio connectens (Kieffer): Kieffer, 1926: 361, 362 (description, keyed); Dodd, 1931: 77 (excluded from Oxyscelio, position uncertain).
Chromoteleia connectens Kieffer: Masner, 1976: 25 (type information); Hoebeke, 1980: 26 (type information); Johnson, 1992: 364 (cataloged, type information).
Chromoteleia brevitarsis Kieffer, 1910a: 313, 315 (original description, keyed); Masner, 1976: 25 (type information, junior synonym of Chromoteleiaconnectens Kieffer); Johnson, 1992: 364 (type information).
Petalosema brevitarsis (Kieffer): Kieffer, 1926: 358, 360 (generic transfer, description, keyed).
http://zoobank.org/C7398CF1-267D-4D5D-B89C-55697354254C
http://bioguid.osu.edu/xbiod_concepts/8526

#### Description.

Body length of female: 5.38–5.95 mm (n = 20). Body length of male: 5.10–5.86 mm (n = 20). Color of A1: yellow to orange. A6 in female: as wide as long. A5 in female: distinctly longer than wide. A6 in male: approximately 2.0× longer than wide. Number of basiconic sensilla on A7: 0. Number of basiconic sensilla on A12: 1. Sculpture of dorsal A1: striate. Color of head: black. Sculpture of frons directly above interantennal process: transversely striate to rugose. Central keel: complete, extending from interantennal process to median ocellus. Ventral margin of clypeus: straight. Granulate microsculpture of dorsal frons: present. Occipital carina: complete. Granulate microsculpture of vertex: absent; present. Sculpture of occiput: rugose. Sculpture of gena: dorsoventrally strigose.Color of mesosoma: variably orange to black. Sculpture of epicoxal lobe posterior of propleural epicoxal sulcus: smooth. Sculpture of lateral pronotal area above pronotal cervical sulcus: smooth throughout. Sculpture of netrion: rugose. Microsculpture of mesoscutum: granulate. Macrosculpture of mesoscutal midlobe: punctate rugose throughout. Macrosculpture of lateral lobe of mesoscutum: punctate rugose. Sculpture of notaulus: foveate. Notaular foveae: interconnected. Median mesoscutal carina: present along full length of mesoscutum. Mesoscutellum in lateral view: convex. Sculpture of mesoscutellum: smooth medially, densely punctate laterally; longitudinally carinate medially, densely punctate laterally. Shape of metascutellum: trapezoidal with broad posterior margin. Median metascutellar carina: present. Sculpture of metascutellum: rugose. Sculpture of lateral propodeal area: rugose; smooth. Mesopleural carina: present. Sculpture of mesepisternum below femoral depression: rugose anteriorly, sparsely punctate posteriorly. Sculpture of dorsal metapleural area: rugose. Setation of dorsal metapleural area: absent. Setation of area directly dorsal to the metapleural triangle: absent. Sculpture of ventral metapleural area: rugose anteriorly, smooth posteriorly. Color of legs: orange yellow throughout; orange to pale brown. Length of hind basitarsus: about as long as remaining segments combined. Sculpture of hind coxa: densely punctate.

Length of postmarginal vein: distinctly longer than stigmal vein.

Color of metasoma in female: black; mostly black with T1–T3 orange to dark brown. Color of metasoma in male: black. Horn on T1 in female: present. Striae of posterior margin of T1 in female: sparse. Striae of T1 in male: sparse. Transverse sulcus on T2: present. Sculpture of T2: densely longitudinally striate, punctate rugulose in interstices. Sculpture of T6 in female: densely punctate and granulate. Length of T6 in female: approximately as long as wide. Shape of T6 in female in lateral view: flat. Apical spine on female T6: absent. Sculpture of T6 in male: densely punctate. Sculpture of T7 in male: granulate. Posterior margin of T7 in male: emarginate medially between rounded projections. Sculpture of medial S2: densely longitudinally striate with fine punctures in interstices.

**Figures 58–63.**
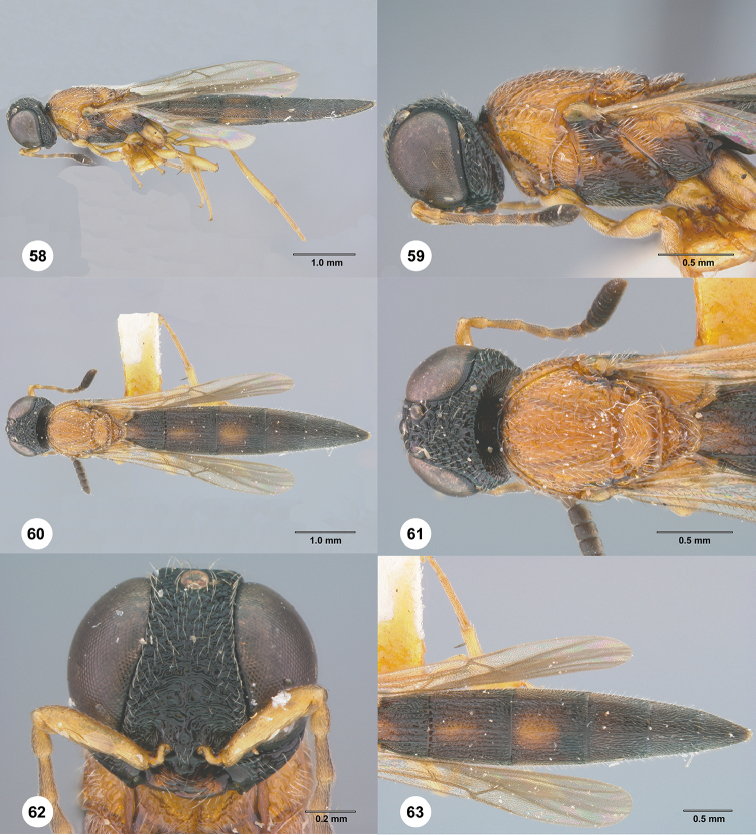
*Chromoteleiaconnectens* Kieffer, female, holotype (CAS TYPE9618). **58** Lateral habitus **59** Head and mesosoma, lateral view **60** Dorsal habitus **61** Head and mesosoma, dorsal view **62** Head, anterior view **63** Metasoma, dorsal view.

**Figures 64–69.**
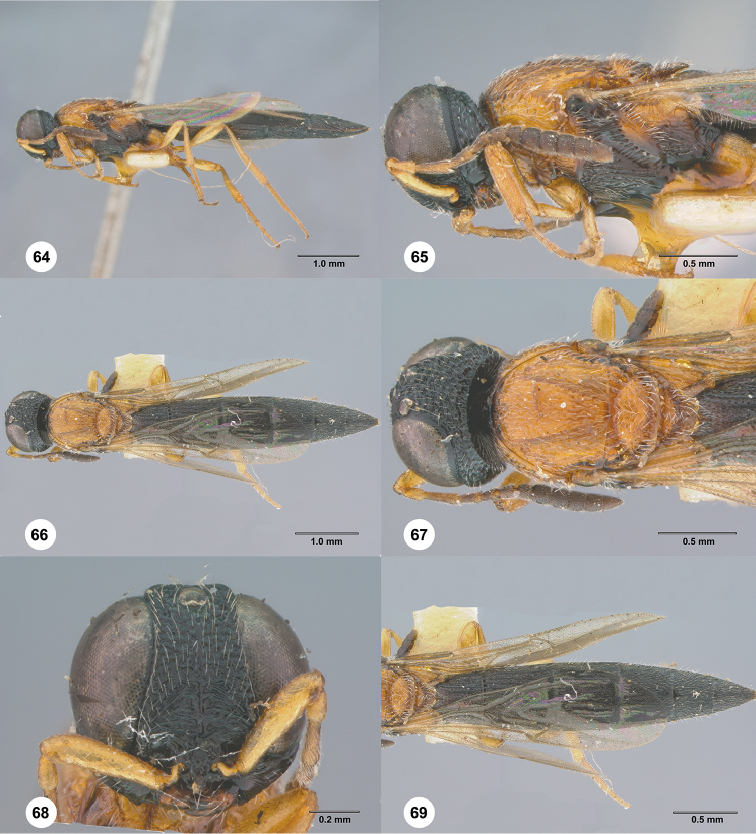
*Chromoteleiabrevitarsis* Kieffer, female, holotype (CAS TYPE9597). **64** Lateral habitus **65** Head and mesosoma, lateral view **66** Dorsal habitus **67** Head and mesosoma, dorsal view **68** Head, anterior view **69** Metasoma, dorsal view.

#### Diagnosis.

This species can be distinguished from other *Chromoteleia* by the following combination of characters: female A7 without basiconic sensillum, occipital carina complete, median mesoscutal carina present along full length of mesoscutum, ventral metapleural area smooth posteriorly, postmarginal vein distinctly longer than stigma vein, male T7 with posterior margin deeply emarginated between rounded projections.

#### Link to distribution map.

[http://hol.osu.edu/map-large.html?id=4212]

#### Material examined.

Holotype, female, *C.brevitarsis*: **BRAZIL**: PA, no date, Baker, CAS TYPE9597 (deposited in CAS). Holotype, female, *C.connectens*: **BRAZIL**: PA, no date, Baker, CAS TYPE9618 (deposited in CAS).

**Other material**: (524 females, 594 males) **BELIZE**: 1 female, OSUC586444 (CNCI). **BOLIVIA**: 5 females, 7 males, OSUC584139–584140, 584298–584300 (CNCI); DPI_FSCA 00010202–00010208 (CSCA). **BRAZIL**: 77 females, 73 males, OSUC202485, 202488, 202490, 202493, 202495–202497, 202499–202501, 202515–202517, 202519, 202523, 202532, 202535 (AEIC); OSUC232991 (BPBM); OSUC583467–583468, 583471, 583473–583475, 583484–583485, 583492, 583495–583496, 583498, 583510, 583516, 584146, 584152, 584161, 584164–584165, 584171–584175, 584177, 584330, 584334, 584336, 586124–586126, 586131–586132, 586134, 586352, 586356, 586358, 586365, 586368, 586394–586395, 586397–586398, 586404, 586406, 586418–586419, 586567–586568, 586796 (CNCI); OSUC199611, 199634 (FSCA); OSUC181648, 225221, 225223, 225240, 231875, 233121, 233201, 233295, 233345, 235221, 267216, 322173, 322599, 326391, 326465, 344484, 354669, 359051, 359057, 359059, 359065, 359067, 359076, 370978, 379216, 380242, 449172, 449186–449187 (MPEG); OSUC112474, 112594, 112933, 132283, 132337–132338 (MZSP); OSUC132223, 132546, 132576, 132614, 225220, 225239, 233200, 233320, 255000, 322166, 322175, 322598, 322639, 326569, 345343, 359056, 359063–359064, 359068, 370976–370977, 379214, 381994, 449168, 449178, 48530, 48535, 56248, 56250, 56257–56258, 56260, 56262 (OSUC); OSUC204987, 204989 (UCDC); OSUC225298–225300, 225302–225304, 225309, 225312, 225314–225315 (USNM). **COLOMBIA**: 49 females, 55 males, OSUC584137, 584301 (CNCI); OSUC143884, 143894–143895, 143897, 143899, 143901, 144022, 152042–152043, 152069, 152071, 162521, 162680–162681, 166453, 166456–166457, 166521, 166555, 170459, 176951, 182582–182583, 182713, 188629–188630, 188632–188633, 188691–188692, 188783, 189043–189044, 189046, 190347–190348, 190350, 190901, 191362, 193136, 193185, 193429, 193456–193458, 193460, 193661, 193664, 193680, 193819, 193960–193962, 228571, 228601, 228603–228605, 228607, 230379, 230434, 230436, 230439, 232724, 232726, 249925–249926, 253478, 269375, 270003, 270005–270007, 321676 (IAVH); OSUC143896, 143898, 152070, 152072, 162520, 162679, 166450, 166458, 166520, 166565, 166588, 170460, 176950, 182581, 189045, 190349, 191043, 191173, 193168, 193184, 193793, 228602, 228606, 232723, 249928, 269377, 270004, 273444 (OSUC). **COSTA RICA**: 145 females, 195 males, 1 unknown, OSUC202537, 202539–202540, 202549, 205189 (AEIC); OSUC232993 (BPBM); OSUC232189, 253965, 253967–253968, 253970–253971, 556950, 556971, 556973–556977, 556980, 556982–556985, 556993, 556995–556996, 556999, 557001, 557004, 557010, 557014, 557018, 557023, 557048, 557050–557051, 557053, 557058, 557084, 557087, 577888–577893, 577895–577896, 577899, 577908, 577927, 577929, 577938, 577940, 577943–577944, 577947, 577949–577951, 577954–577955, 577962, 577964, 577971, 577974–577978, 577980–577984, 577986–577987, 577989–577990, 577992–577999, 578001, 578007, 578009, 578012–578013, 578018, 578020–578022, 578025–578043, 578045, 578047–578052, 578066, 578073, 578076, 578080, 578085, 578092, 578097, 578099, 578104, 583413, 583415–583416, 583422–583423, 583461, 583479, 583482, 583508, 583511–583513, 583731, 583739, 583756, 583760, 583763–583764, 583768–583771, 583773, 583787, 583794, 583796, 583834, 583850, 583878, 583881, 583890–583892, 583896–583897, 583901, 583910, 583916, 583926–583928, 583931–583938, 583947–583948, 583978, 583980, 583982, 583988–583990, 583992, 583994, 583997–583998, 584003–584006, 584008–584009, 584012, 584014, 584016–584017, 584019, 584023, 584026–584028, 584031, 584226, 584228, 584231–584232, 584234, 584237, 584239, 584241, 584245–584246, 584248–584249, 584251, 584253–584259, 584271–584273, 584277–584280, 584284–584286, 584288–584293, 586121, 586123, 586130, 586150–586151, 586161, 586163–586164, 586166–586168, 586171, 586179–586181, 586183, 586194, 586196, 586198, 586200–586201, 586203, 586205–586206, 586209, 586212–586214, 586216, 586260–586261, 586268–586269, 586271–586274, 586293–586295, 586302, 586357, 586390, 586399, 586415, 586449, 586451, 586471, 586474–586476, 586485, 586493, 586499, 586501, 586510, 586513, 586516–586520, 586541–586543, 586559, 586619–586620, 586638–586640, 586645, 586648–586649, 586656, 586658–586659, 586679, 586681, 586764, 586778, 586781, 586783, 586785–586787, 586831, 586833, 586837, 586839, 586841 (CNCI); DPI_FSCA 00010229–00010230, SM0810076, SM0810559 (KUNH); OSUC205812 (UCDC). **ECUADOR**: 122 females, 166 males, OSUC149644, 202502 (AEIC); OSUC181428, 557030, 557034, 557037, 557042–557045, 557092–557094, 557096, 557098, 583476–583477, 583751, 584047–584059, 584061–584073, 584075–584082, 584084–584096, 584099–584101, 584105, 584110–584111, 584120–584121, 584123–584130, 584132, 584134–584136, 584399, 584401, 584403, 584405–584406, 584408, 584411–584413, 584415, 584427, 584430, 584432–584451, 584807–584816, 584818–584837, 584839–584856, 585089–585090, 585097, 586142–586144, 586178, 586311–586313, 586317–586319, 586335, 586359, 586413, 586546–586553, 586555–586556, 586558, 586625, 586633, 586668–586670, 586672–586676, 586693, 586696–586707, 586709–586713, 586715–586720, 586723, 586725–586726, 586728–586730, 586732–586746, 586873 (CNCI); DPI_FSCA 00010184–00010201, 00010209 (CSCA); OSUC199615–199629 (FSCA); OSUC221931, 221939 (TAMU); OSUC204993, 204995–204996, 204998 (UCDC). **FRENCH GUIANA**: 36 females, 28 males, OSUC555804, 555814, 555819, 586222, 586225, 586228–586230, 586232, 586234–586238, 586240–586242, 586244–586245, 586247–586255, 586257, 586423, 586427, 586434, 586436, 586439, 586443, 586446, 586453–586458, 586461–586464, 586609–586612, 586830, 586844–586845, 586850–586851, 586854–586855, 586859 (CNCI); SM0096678 (KUNH); OSUC267215, 47019–47021, 47023 (OSUC). **GUATEMALA**: 2 females, 1 male, OSUC203118 (AEIC); OSUC584925 (CNCI); OSUC204701 (UCDC). HONDURAS: 1 male, OSUC413764 (MZLU). **PANAMA**: 8 females, 8 males, OSUC556951, 584182, 584379, 586623v586624, 586626, 586629, 586824 (CNCI); DPI_FSCA 00010227 (KUNH); OSUC221921, 233027, 271017–271020, 320637 (TAMU). **PERU**: 24 females, 17 males, OSUC149639 (AEIC); OSUC578057, 578060, 578062, 584305, 584307–584308, 584314–584316, 586135–586137, 586185–586188, 586190–586192, 586421–586422, 586441–586442, 586805, 586808–586814, 586846, 586865 (CNCI); OSUC218803–218805 (INHS); DPI_FSCA 00010228 (KUNH); OSUC223890, 323994 (OSUC); OSUC225399 (USNM). **VENEZUELA**: 55 females, 43 males, OSUC149646 (AEIC); OSUC578015, 583517, 586145 (CNCI); OSUC230357–230359, 230361, 232281, 232827–232830, 251645–251647, 251649, 251651–251652, 251655, 251657–251661, 251663–251666, 251668–251670, 251672–251675, 251677–251681, 251683–251684, 251686–251688, 251690, 320743–320745, 320747–320753, 321367, 321369–321371, 321373–321374, 321376–321379, 321382, 323405, 323407–323412, 323414, 381986–381987, 381989, 381991–381992 (MIZA); OSUC230360, 232282, 251643, 251648, 251650, 251653, 251656, 251671, 251682, 251685, 251692, 321368, 321372, 321375, 321380–321381, 323406 (OSUC).

#### Comments.

This species exhibits variation in color and microsculpture. The development of microsculpture on vertex could be absent or present; mesoscutum smooth or carinate medially; mesoscutellum, mesopleuron, and metapleuron ranging from orange to dark brown or black.

### Chromoteleia copiosa

Taxon classificationAnimaliaHymenopteraScelionidae

Chen & Johnson sp. n.

http://zoobank.org/E61DC4D8-138F-4525-8A8A-79476002AAA8

http://bioguid.osu.edu/xbiod_concepts/452220

Figures 70–75

#### Description.

Body length of female: 4.78–5.76 mm (n = 20). Body length of male: 4.30–5.33 mm (n = 20). Color of A1: yellow to orange. A6 in female: as wide as long. A5 in female: distinctly longer than wide. A6 in male: as long as wide. Number of basiconic sensilla on A7: 1. Number of basiconic sensilla on A12: 1. Sculpture of dorsal A1: punctate. Color of head: black. Sculpture of frons directly above interantennal process: areolate. Central keel: complete, extending from interantennal process to median ocellus. Ventral margin of clypeus: pointed. Granulate microsculpture of dorsal frons: absent. Occipital carina: interrupted medially. Granulate microsculpture of vertex: absent. Sculpture of occiput: smooth. Sculpture of gena: punctate rugose dorsally and ventrally, strigose medially.

Color of mesosoma: variably orange to black. Sculpture of epicoxal lobe posterior of propleural epicoxal sulcus: smooth. Sculpture of lateral pronotal area above pronotal cervical sulcus: smooth dorsally, rugose ventrally. Sculpture of netrion: transversely striate. Microsculpture of mesoscutum: granulate. Macrosculpture of mesoscutal midlobe: punctate rugose anteriorly, sparsely punctate posteriorly. Macrosculpture of lateral lobe of mesoscutum: densely punctate. Sculpture of notaulus: foveate. Notaular foveae: interconnected. Median mesoscutal carina: present anteriorly, not extending to posterior margin of mesoscutum. Mesoscutellum in lateral view: convex. Sculpture of mesoscutellum: smooth medially, densely punctate laterally. Shape of metascutellum: trapezoidal with broad posterior margin. Median metascutellar carina: present. Sculpture of metascutellum: rugose. Sculpture of lateral propodeal area: rugose. Mesopleural carina: present. Sculpture of mesepisternum below femoral depression: punctate rugose. Sculpture of dorsal metapleural area: rugose. Setation of dorsal metapleural area: absent. Setation of area directly dorsal to the metapleural triangle: present. Sculpture of ventral metapleural area: rugose throughout. Color of legs: orange yellow throughout. Length of hind basitarsus: distinctly longer than remaining segments combined. Sculpture of hind coxa: densely punctate.

Length of postmarginal vein: approximately as long as stigmal vein.

Color of metasoma in female: black. Color of metasoma in male: black. Horn on T1 in female: present. Striae of posterior margin of T1 in female: dense. Striae of T1 in male: sparse; dense. Transverse sulcus on T2: present. Sculpture of T2: densely longitudinally striate, punctate rugulose in interstices. Sculpture of T6 in female: densely longitudinally striate, with fine punctures in interstices. Length of T6 in female: at least 1.5× longer than wide. Shape of T6 in female in lateral view: flat. Apical spine on female T6: absent. Sculpture of T6 in male: densely longitudinally striate with fine punctures in interstices. Sculpture of T7 in male: coriaceous anteriorly, densely punctate posteriorly. Posterior margin of T7 in male: straight. Sculpture of medial S2: densely longitudinally striate with fine punctures in interstices.

**Figures 70–75.**
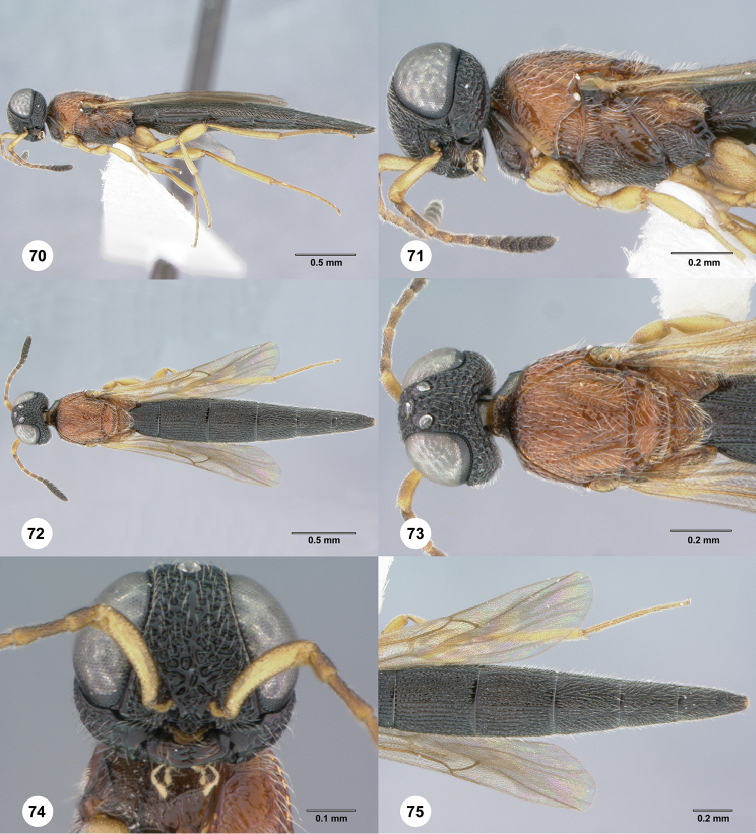
*Chromoteleiacopiosa* sp. n., female, holotype (OSUC577518). **70** Lateral habitus **71** Head and mesosoma, lateral view **72** Dorsal habitus **73** Head and mesosoma, dorsal view **74** Head, anterior view **75** Metasoma, dorsal view.

#### Diagnosis.

This species is similar to *C.sparsa*, but it can be distinguished by its medially interrupted occipital carina, transversely striate netrion, and the hind basitarsus that is distinctly longer than the remaining segments combined.

#### Etymology.

The epithet is inspired by the Latin word for abundant, in reference to the densely punctate rugose ventral mesepisternum, and is intended to be treated as an adjective.

#### Link to distribution map.

[http://hol.osu.edu/map-large.html?id=452220]

#### Material examined.

Holotype, female: **BELIZE**: Orange Walk Dist., Rio Bravo Conservation and Management Area, 15.VII–21.VII.1996, Malaise trap, P. Kovarik, OSUC577518 (deposited in CNCI). *Paratypes*: (54 females, 10 males) **BELIZE**: 11 females, OSUC556953–556954, 556966, 577508, 577511–577512, 577516–577517, 586761 (CNCI); OSUC254866–254867 (OSUC). **BRAZIL**: 1 male, OSUC584162 (CNCI). COLOMBIA: 1 female, OSUC584304 (CNCI). **COSTA RICA**: 33 females, 6 males, OSUC577902, 577910–577911, 577914–577916, 577918–577922, 583913, 583954, 583956, 583958–583959, 583962, 583964–583965, 583967–583970, 583972–583975, 584033 (CNCI); OSUC577909, 577913, 577917, 583955, 583957, 583960–583961, 583963, 583966, 583971, 586162 (OSUC). **GUATEMALA**: 2 females, OSUC577513, 577520 (CNCI). **HONDURAS**: 1 male, OSUC577515 (CNCI). **MEXICO**: 4 females, 2 males, OSUC577507, 577514, 577519, 584721 (CNCI); OSUC271012 (OSUC); OSUC271015 (UNAM). **PANAMA**: 1 female, OSUC584382 (CNCI). **TRINIDAD AND TOBAGO**: 1 female, OSUC583478 (CNCI). **VENEZUELA**: 1 female, OSUC586289 (CNCI).

### Chromoteleia cuneus

Taxon classificationAnimaliaHymenopteraScelionidae

Chen & Johnson sp. n.

http://zoobank.org/BD48157A-CEA8-45EA-BCC2-11E2807CA094

http://bioguid.osu.edu/xbiod_concepts/452221

Figures 13 , 76–81

#### Description.

Body length of female: 4.88–5.10 mm (n = 20). Body length of male: 4.30–4.89 mm (n = 20). Color of A1: yellow to orange. A6 in female: as wide as long. A5 in female: distinctly longer than wide. A6 in male: approximately 2.0× longer than wide. Number of basiconic sensilla on A7: 1. Number of basiconic sensilla on A12: 1. Sculpture of dorsal A1: punctate; smooth. Color of head: black. Sculpture of frons directly above interantennal process: areolate. Central keel: present only in ventral portion of frons. Ventral margin of clypeus: pointed. Granulate microsculpture of dorsal frons: absent. Occipital carina: complete. Granulate microsculpture of vertex: absent. Sculpture of occiput: smooth. Sculpture of gena: coarsely punctate rugose.

Color of mesosoma: variably orange to black. Sculpture of epicoxal lobe posterior of propleural epicoxal sulcus: smooth. Sculpture of lateral pronotal area above pronotal cervical sulcus: smooth dorsally, rugose ventrally. Sculpture of netrion: transversely striate. Microsculpture of mesoscutum: granulate. Macrosculpture of mesoscutal midlobe: punctate rugose anteriorly, sparsely punctate posteriorly. Macrosculpture of lateral lobe of mesoscutum: punctate rugose. Sculpture of notaulus: foveate. Notaular foveae: interconnected. Median mesoscutal carina: present anteriorly, not extending to posterior margin of mesoscutum. Mesoscutellum in lateral view: convex. Sculpture of mesoscutellum: smooth medially, densely punctate laterally. Shape of metascutellum: trapezoidal with broad posterior margin. Median metascutellar carina: present. Sculpture of metascutellum: rugose. Sculpture of lateral propodeal area: rugose. Mesopleural carina: present. Sculpture of mesepisternum below femoral depression: largely smooth, punctate rugose anteriorly and directly below femoral depression. Sculpture of dorsal metapleural area: rugose. Setation of dorsal metapleural area: absent. Setation of area directly dorsal to the metapleural triangle: present. Sculpture of ventral metapleural area: rugose throughout. Color of legs: orange yellow to brown, with tarsi darker. Length of hind basitarsus: about as long as remaining segments combined. Sculpture of hind coxa: densely punctate.

Length of postmarginal vein: distinctly shorter than stigmal vein.

Color of metasoma in female: black. Color of metasoma in male: black. Horn on T1 in female: present. Striae of posterior margin of T1 in female: dense. Striae of T1 in male: dense. Transverse sulcus on T2: present. Sculpture of T2: densely longitudinally striate, punctate rugulose in interstices. Sculpture of T6 in female: longitudinally punctate rugose. Length of T6 in female: at least 1.5× longer than wide. Shape of T6 in female in lateral view: sinuate. Apical spine on female T6: present. Sculpture of T6 in male: densely punctate. Sculpture of T7 in male: coriaceous anteriorly, densely punctate posteriorly. Posterior margin of T7 in male: emarginate medially between rounded projections. Sculpture of medial S2: densely longitudinally striate with fine punctures in interstices.

**Figures 76–81.**
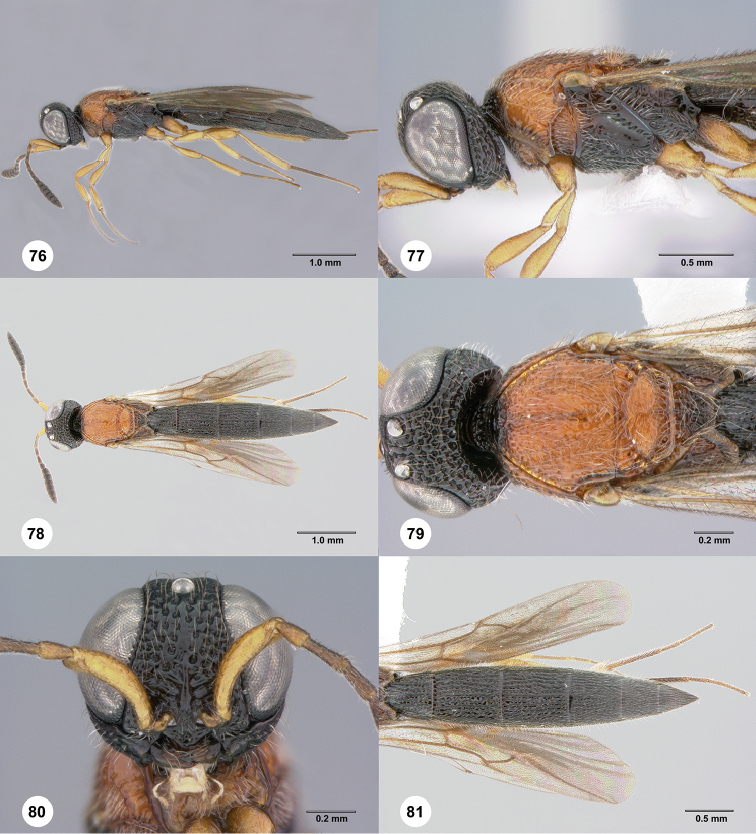
*Chromoteleiacuneus* sp. n., female, holotype (OSUC585001). **76** Lateral habitus **77** Head and mesosoma, lateral view **78** Dorsal habitus **79** Head and mesosoma, dorsal view **80** Head, anterior view **81** Metasoma, dorsal view.

#### Diagnosis.

This species can be distinguished from other *Chromoteleia* by the following combination of characters: frons with central keel developed only in ventral portion of frons, dorsal metapleural area without setae, postmarginal vein distinctly shorter than stigmal vein, apex of T6 acute in dorsal view, male T7 with posterior margin deeply emarginate medially between rounded projections.

#### Etymology.

The epithet is inspired by the Latin word for wedge, in reference to the shape of T6 in female, and is intended to be treated as a noun.

#### Link to distribution map.

[http://hol.osu.edu/map-large.html?id=452221]

#### Material examined.

Holotype, female: **ECUADOR**: Santo Domingo de los Tsáchilas Prov., 16 km SE Santo Domingo de los Colorados, Tinalandia, 500 m, 4.VI–14.VI.1976, S. Peck & J. Peck, OSUC585001 (deposited in CNCI). *Paratypes*: (61 females, 52 males) **COSTA RICA**: 2 females, OSUC577898, 586775 (CNCI). **ECUADOR**: 58 females, 52 males, OSUC202533 (AEIC); OSUC583757, 584074, 584107–584109, 584416–584419, 584421–584422, 584424, 584426, 584758, 585003–585004, 585006–585012, 585014–585021, 585023–585028, 585030–585043, 585045–585076, 585078, 585080, 585082–585088, 586327–586328, 586635, 586847–586848 (CNCI); OSUC369619 (MZLU); OSUC584420, 584423, 584425, 585002, 585005, 585013, 585022, 585029, 585077, 585079, 585081 (OSUC). **PANAMA**: 1 female, OSUC271021 (TAMU). *Other material*: **ECUADOR**: 2 females, 1 male, DPI_FSCA 00010213–00010215 (CSCA).

### Chromoteleia curta

Taxon classificationAnimaliaHymenopteraScelionidae

Chen & Johnson sp. n.

http://zoobank.org/E661A7C8-E5DA-4F9A-8507-6D8447DC59AA

http://bioguid.osu.edu/xbiod_concepts/318230

Figures 82–87 , 88–93 , 239

#### Description.

Body length of female: 4.23–5.27 mm (n = 20). Body length of male: 4.10–5.13 mm (n = 20). Color of A1: yellow to orange. A6 in female: as wide as long. A5 in female: distinctly longer than wide. A6 in male: as long as wide. Number of basiconic sensilla on A7: 1. Number of basiconic sensilla on A12: 1. Sculpture of dorsal A1: punctate. Color of head: black. Sculpture of frons directly above interantennal process: punctate rugose. Central keel: present, interrupted medially. Ventral margin of clypeus: pointed. Granulate microsculpture of dorsal frons: absent. Occipital carina: interrupted medially. Granulate microsculpture of vertex: absent. Sculpture of occiput: smooth. Sculpture of gena: narrowly smooth ventrally, punctate rugose dorsally.

Color of mesosoma: variably orange to black. Sculpture of epicoxal lobe posterior of propleural epicoxal sulcus: densely punctate. Sculpture of lateral pronotal area above pronotal cervical sulcus: smooth dorsally, rugose ventrally. Sculpture of netrion: rugose. Microsculpture of mesoscutum: granulate. Macrosculpture of mesoscutal midlobe: punctate rugose anteriorly, sparsely punctate posteriorly. Macrosculpture of lateral lobe of mesoscutum: sparsely punctate. Sculpture of notaulus: foveate. Notaular foveae: interconnected. Median mesoscutal carina: present anteriorly, not extending to posterior margin of mesoscutum. Mesoscutellum in lateral view: flat. Sculpture of mesoscutellum: smooth medially, densely punctate laterally. Shape of metascutellum: trapezoidal with broad posterior margin. Median metascutellar carina: absent or indistinguishable from sculpture. Sculpture of metascutellum: rugose. Sculpture of lateral propodeal area: rugose. Mesopleural carina: present. Sculpture of mesepisternum below femoral depression: punctate throughout. Sculpture of dorsal metapleural area: rugose. Setation of dorsal metapleural area: absent. Setation of area directly dorsal to the metapleural triangle: present. Sculpture of ventral metapleural area: rugose throughout. Color of legs: orange yellow throughout. Length of hind basitarsus: distinctly longer than remaining segments combined. Sculpture of hind coxa: largely smooth, with sparse fine punctures.

Length of postmarginal vein: approximately as long as stigmal vein.

Color of metasoma in female: black. Horn on T1 in female: present. Striae of posterior margin of T1 in female: dense. Striae of T1 in male: sparse. Transverse sulcus on T2: present. Sculpture of T2: densely longitudinally striate, punctate rugulose in interstices. Sculpture of T6 in female: longitudinally punctate rugose. Length of T6 in female: approximately as long as wide. Shape of T6 in female in lateral view: flat. Apical spine on female T6: absent. Sculpture of T6 in male: densely longitudinally striate with fine punctures in interstices. Sculpture of T7 in male: granulate. Posterior margin of T7 in male: straight. Sculpture of medial S2: densely longitudinally striate with fine punctures in interstices.

**Figures 82–87.**
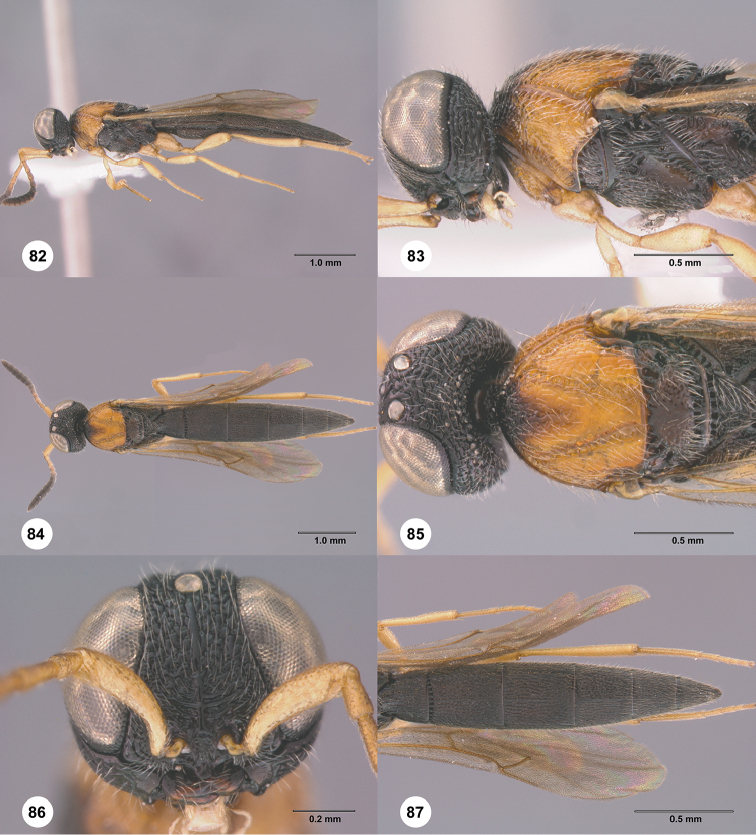
*Chromoteleiacurta* sp. n., female, holotype (OSUC185626). **82** Lateral habitus **83** Head and mesosoma, lateral view **84** Dorsal habitus **85** Head and mesosoma, dorsal view **86** Head, anterior view **87** Metasoma, dorsal view. (NOTE: light form)

**Figures 88–93.**
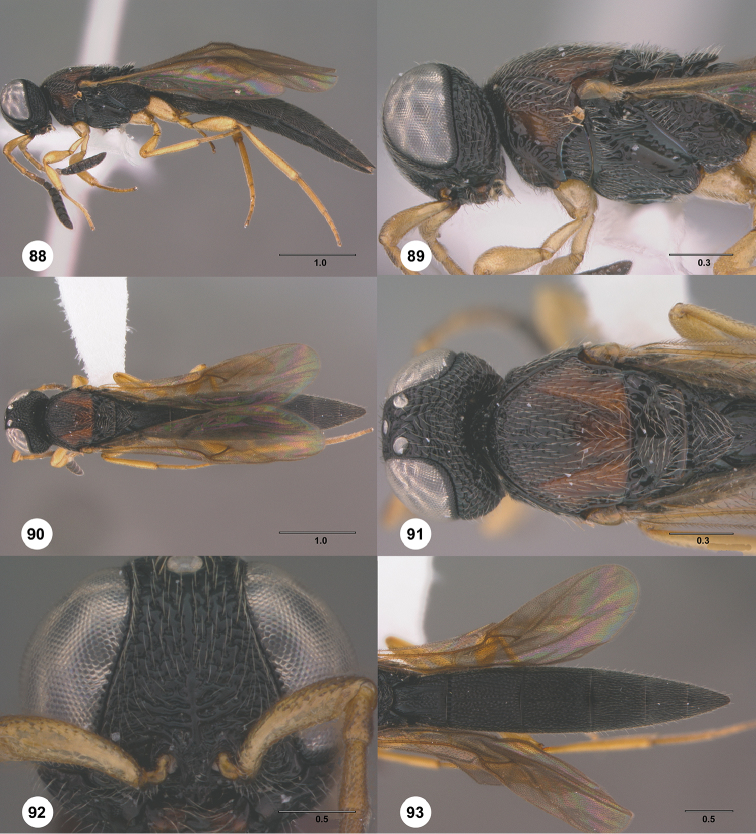
*Chromoteleiacurta* sp. n., female, paratype (OSUC149925). **88** Lateral habitus **89** Head and mesosoma, lateral view **90** Dorsal habitus **91** Head and mesosoma, dorsal view **92** Head, anterior view **93** Metasoma, dorsal view. (NOTE: dark form)

#### Diagnosis.

This species is similar to *C.parvitas*, but it can be distinguished by the combination of the following characters: A5 in female distinctly longer than wide, postmarginal vein approximately as long as stigmal vein, female T1 horn present.

#### Etymology.

The epithet is inspired by the Latin word for shortened, in reference to the short median mesoscutal carina, and is intended to be treated as an adjective.

#### Link to distribution map.

[http://hol.osu.edu/map-large.html?id=318230]

#### Material examined.

Holotype, female: **BRAZIL**: SP, Trilha da Anta, Base Barra Grande, YPT B0, Intervales State Park, 11.XII–14.XII.2000, yellow pan trap, M. T. Tavares, OSUC185626 (deposited in MZSP). *Paratypes*: (22 females, 25 males) **BRAZIL**: 13 females, 19 males, OSUC202509 (AEIC); OSUC578000, 583491, 586148, 586396 (CNCI); OSUC126962, 129282, 147965, 148090, 148098, 148100, 149925, 149996, 150330, 150442, 185627, 263040, 66311, 79986 (MZSP); OSUC127095, 128181, 128644, 147683–147684, 147730, 148057, 148059, 148089, 149844, 149997, 185628, 79987 (OSUC). **MEXICO**: 1 male, OSUC586432 (CNCI). **VENEZUELA**: 9 females, 5 males, OSUC578014, 578016–578017, 584142 (CNCI); OSUC251654, 251689, 376068, 376070–376071 (MIZA); OSUC376069, 376072–376073, 376075, 381988 (OSUC).

#### Comments.

This species is extremely variable in mesosoma color and can be roughly divided into dark and light forms.

### Chromoteleia depilis

Taxon classificationAnimaliaHymenopteraScelionidae

Chen & Johnson sp. n.

http://zoobank.org/30F7A6B0-982F-4A20-8ACC-EDA6322C9E22

http://bioguid.osu.edu/xbiod_concepts/452216

Figures 18–19 , 28 , 94–99

#### Description.

Body length of female: 5.48–7.65 mm (n = 20). Body length of male: 4.34–5.85 mm (n = 20). Color of A1: yellow to orange. A6 in female: distinctly longer than wide. A5 in female: distinctly longer than wide. A6 in male: approximately 2.0× longer than wide. Number of basiconic sensilla on A7: 0. Number of basiconic sensilla on A12: 2. Sculpture of dorsal A1: striate. Color of head: black. Sculpture of frons directly above interantennal process: transversely striate to rugose. Central keel: complete, extending from interantennal process to median ocellus. Ventral margin of clypeus: pointed. Granulate microsculpture of dorsal frons: present. Occipital carina: interrupted medially. Granulate microsculpture of vertex: absent. Sculpture of occiput: rugose. Sculpture of gena: dorsoventrally strigose.

Color of mesosoma: variably orange to black; orange. Sculpture of epicoxal lobe posterior of propleural epicoxal sulcus: smooth. Sculpture of lateral pronotal area above pronotal cervical sulcus: smooth throughout. Sculpture of netrion: transversely striate. Microsculpture of mesoscutum: granulate. Macrosculpture of mesoscutal midlobe: punctate rugose throughout. Macrosculpture of lateral lobe of mesoscutum: punctate rugose. Sculpture of notaulus: foveate. Notaular foveae: discrete. Median mesoscutal carina: present along full length of mesoscutum. Mesoscutellum in lateral view: convex. Sculpture of mesoscutellum: smooth medially, densely punctate laterally. Shape of metascutellum: trapezoidal with broad posterior margin. Median metascutellar carina: absent or indistinguishable from sculpture. Sculpture of metascutellum: rugose. Sculpture of lateral propodeal area: rugose. Mesopleural carina: absent; present. Sculpture of mesepisternum below femoral depression: smooth directly below femoral depression, otherwise densely punctate. Sculpture of dorsal metapleural area: rugose. Setation of dorsal metapleural area: absent. Setation of area directly dorsal to the metapleural triangle: present. Sculpture of ventral metapleural area: rugose throughout. Color of legs: orange yellow to brown, with tarsi darker. Length of hind basitarsus: about as long as remaining segments combined. Sculpture of hind coxa: densely punctate.

Length of postmarginal vein: distinctly longer than stigmal vein.

Color of metasoma in female: black. Color of metasoma in male: black. Horn on T1 in female: present. Striae of posterior margin of T1 in female: dense. Striae of T1 in male: dense. Transverse sulcus on T2: present. Sculpture of T2: densely longitudinally striate, punctate rugulose in interstices. Sculpture of T6 in female: densely longitudinally striate, with fine punctures in interstices. Length of T6 in female: at least 1.5× longer than wide. Shape of T6 in female in lateral view: flat. Apical spine on female T6: absent. Sculpture of T6 in male: densely longitudinally striate with fine punctures in interstices. Sculpture of T7 in male: smooth to coriaceous. Posterior margin of T7 in male: straight. Sculpture of medial S2: densely longitudinally striate with fine punctures in interstices.

**Figures 94–99.**
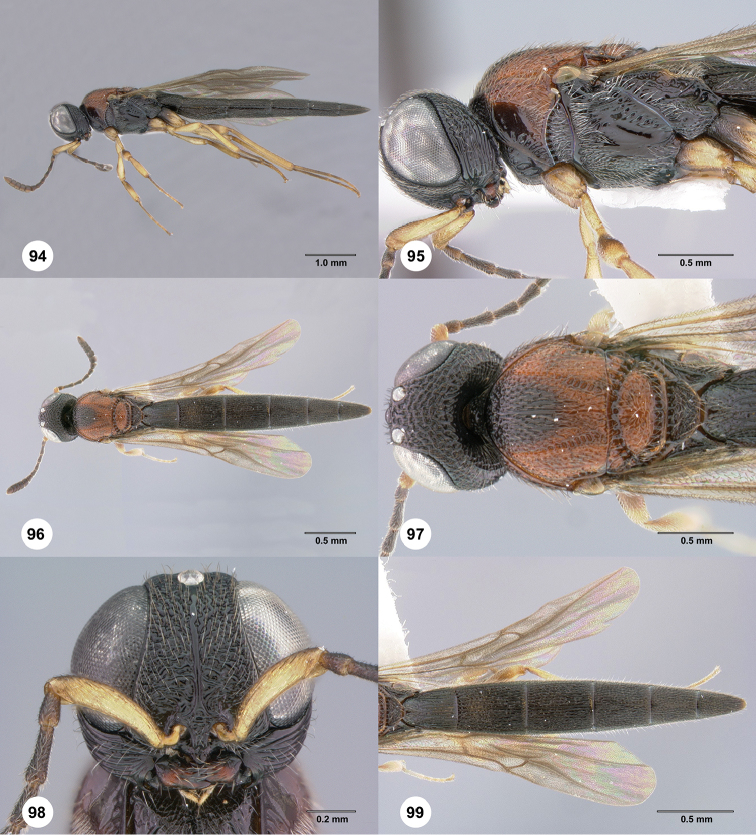
*Chromoteleiadepilis* sp. n., female, holotype (OSUC577436). **94** Lateral habitus **95** Head and mesosoma, lateral view **96** Dorsal habitus **97** Head and mesosoma, dorsal view **98** Head, anterior view **99** Metasoma, dorsal view.

#### Diagnosis.

This species is similar to *C.pilus*, but it can be distinguished by the combination of the following characters: dorsal metapleural area without setation, A6 in female distinctly longer than wide.

#### Etymology.

The epithet is inspired by the Latin word for hairless, in reference to the hairless dorsal metapleural area, and is intended to be treated as an adjective.

#### Link to distribution map.

[http://hol.osu.edu/map-large.html?id=452216]

#### Material examined.

Holotype, female: **GUATEMALA**: Zacapa Dept., 3.5 km SE La Unión, 1500 m, 20.VI–27.VI.1993, flight intercept trap, J. Ashe & R. Brooks, OSUC577436 (deposited in CNCI). *Paratypes*: (75 females, 38 males) **BELIZE**: 11 females, 1 male, OSUC556955, 583463–583464, 584746–584747, 586420, 586426, 586748–586750, 586759–586760 (CNCI). **COSTA RICA**: 23 females, 12 males, OSUC232059, 232187, 253969, 556990–556991, 557062, 577484–577487, 583436, 583889, 583952–583953, 583981, 584042, 584045, 584767–584777, 586487, 586489, 586494, 586500, 586534, 586538 (CNCI); OSUC369615 (MZLU). **EL SALVADOR**: 2 females, 1 male, OSUC583419, 586840, 586842 (CNCI). **GUATEMALA**: 3 females, 3 males, OSUC234526 (BPBM); OSUC577437–577439, 584728–584729 (CNCI). **HONDURAS**: 5 females, OSUC199605 (FSCA); OSUC369623, 369625, 413765, 413772 (MZLU). **MEXICO**: 31 females, 16 males, OSUC149648–149649, 202503 (AEIC); OSUC577496–577498, 584726–584727, 584730–584745, 586428–586431 (CNCI); OSUC271006–271010, 321342, 322559, 372145, 377930, 415086–415089, 49325, 576180–576181, 576183 (OSUC); OSUC221930, 221934 (TAMU). **PANAMA**: 3 males, OSUC202560 (AEIC); OSUC232813, 319210 (TAMU). **VENEZUELA**: 2 males, OSUC376076–376077 (MIZA).

### Chromoteleia dispar

Taxon classificationAnimaliaHymenopteraScelionidae

Chen & Masner sp. n.

http://zoobank.org/C811B539-CFDC-4A2E-8EB7-D9664F889DC7

http://bioguid.osu.edu/xbiod_concepts/318239

Figures 100–105

#### Description.

Body length of female: 4.88–5.50 mm (n = 20). Body length of male: 4.60–5.29 mm (n = 20). Color of A1: yellow to orange. A6 in female: distinctly wider than long. A5 in female: as long as wide. A6 in male: as long as wide. Number of basiconic sensilla on A7: 1. Number of basiconic sensilla on A12: 1. Sculpture of dorsal A1: punctate. Color of head: black. Sculpture of frons directly above interantennal process: areolate. Central keel: absent. Ventral margin of clypeus: pointed. Granulate microsculpture of dorsal frons: absent. Occipital carina: complete. Granulate microsculpture of vertex: absent. Sculpture of occiput: smooth medially, striate laterally. Sculpture of gena: coarsely punctate rugose.

Color of mesosoma: black. Sculpture of epicoxal lobe posterior of propleural epicoxal sulcus: densely punctate. Sculpture of lateral pronotal area above pronotal cervical sulcus: smooth throughout. Sculpture of netrion: transversely striate. Microsculpture of mesoscutum: granulate. Macrosculpture of mesoscutal midlobe: two rows of foveate grooves along median mesoscutal carina anteriorly, smooth at posterior margin. Macrosculpture of lateral lobe of mesoscutum: sparsely punctate. Sculpture of notaulus: foveate. Notaular foveae: interconnected. Median mesoscutal carina: present anteriorly, not extending to posterior margin of mesoscutum. Mesoscutellum in lateral view: convex. Sculpture of mesoscutellum: densely punctate rugose. Shape of metascutellum: trapezoidal with broad posterior margin. Median metascutellar carina: present. Sculpture of metascutellum: areolate. Sculpture of lateral propodeal area: rugose. Mesopleural carina: present. Sculpture of mesepisternum below femoral depression: punctate rugose. Sculpture of dorsal metapleural area: rugose. Setation of dorsal metapleural area: absent. Setation of area directly dorsal to the metapleural triangle: present. Sculpture of ventral metapleural area: rugose throughout. Color of legs: orange yellow throughout. Length of hind basitarsus: about as long as remaining segments combined. Sculpture of hind coxa: densely punctate.

Length of postmarginal vein: distinctly longer than stigmal vein.

Color of metasoma in female: orange. Color of metasoma in male: black. Horn on T1 in female: present. Striae of posterior margin of T1 in female: sparse. Striae of T1 in male: sparse. Transverse sulcus on T2: present. Sculpture of T2: longitudinally punctate rugose. Sculpture of T6 in female: longitudinally punctate rugose. Length of T6 in female: approximately as long as wide. Shape of T6 in female in lateral view: flat. Apical spine on female T6: absent. Sculpture of T6 in male: densely punctate. Sculpture of T7 in male: smooth to coriaceous. Sculpture of medial S2: densely longitudinally striate with fine punctures in interstices.

**Figures 100–105.**
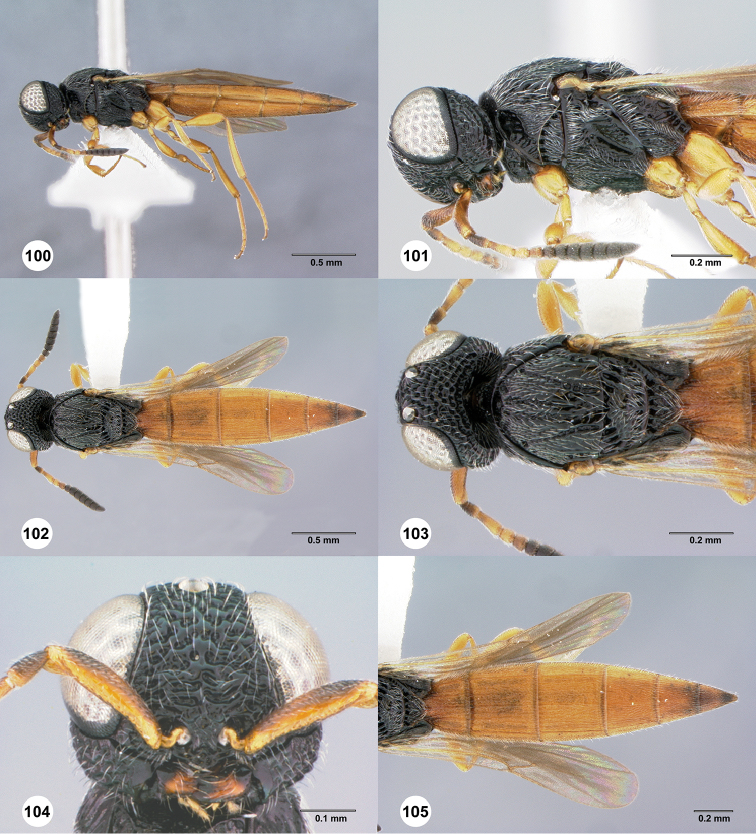
*Chromoteleiadispar* sp. n., female, holotype (OSUC190774). **100** Lateral habitus **101** Head and mesosoma, lateral view **102** Dorsal habitus **103** Head and mesosoma, dorsal view **104** Head, anterior view **105** Metasoma, dorsal view.

#### Diagnosis.

This species can be easily distinguished by the orange female metasoma, the lack of central keel, and the two rows of foveate grooves along median mesoscutal carina anteriorly.

#### Etymology.

The epithet is inspired by the Latin word for different, in reference to the different colors of metasoma between female and male, and is intended to be treated as an adjective.

#### Link to distribution map.

[http://hol.osu.edu/map-large.html?id = 318239]

#### Material examined.

Holotype, female: **COLOMBIA**: Magdalena Dept., Zaino, M.567, 50 m, 11°20'N, 74°02'W, Tayrona Natural National Park, 28.VII–14.VIII.2000, Malaise trap, R. Henriquez, OSUC190774 (deposited in IAVH). *Paratypes*: (22 females, 28 males) **COLOMBIA**: 11 females, 17 males, OSUC586769–586770 (CNCI); OSUC151957–151959, 151961, 152023, 170413, 190080, 190082, 190773 (IAVH); OSUC151960, 152020, 152022, 170414, 190081, 223860, 223866 (OSUC); OSUC223857–223859, 223861–223865, 223867–223868 (USNM). **VENEZUELA**: 11 females, 11 males, OSUC577442–577443, 577446–577448 (CNCI); OSUC237132, 377646 (MIZA); OSUC237133–237135, 271022–271028, 577441, 577445, 586469 (OSUC); OSUC221940 (TAMU); OSUC577444 (USNM).

### Chromoteleia feng

Taxon classificationAnimaliaHymenopteraScelionidae

Chen & Johnson sp. n.

http://zoobank.org/A252B7DB-459E-44BB-97A8-72AC6EC76161

http://bioguid.osu.edu/xbiod_concepts/452223

Figures 106–111

#### Description.

Body length of female: 4.48–5.15 mm (n = 20). Body length of male: 4.30–5.12 mm (n = 20). Color of A1: yellow to orange. A6 in female: distinctly wider than long. A5 in female: distinctly longer than wide. A6 in male: as long as wide. Number of basiconic sensilla on A7: 0. Number of basiconic sensilla on A12: 1. Sculpture of dorsal A1: striate. Color of head: black. Sculpture of frons directly above interantennal process: transversely striate to rugose. Central keel: present only in ventral portion of frons. Ventral margin of clypeus: pointed. Granulate microsculpture of dorsal frons: present. Occipital carina: complete. Granulate microsculpture of vertex: absent. Sculpture of occiput: smooth. Sculpture of gena: narrowly smooth ventrally, punctate rugose dorsally.

Color of mesosoma: orange. Sculpture of epicoxal lobe posterior of propleural epicoxal sulcus: smooth. Sculpture of lateral pronotal area above pronotal cervical sulcus: smooth throughout. Sculpture of netrion: transversely striate. Microsculpture of mesoscutum: granulate. Macrosculpture of mesoscutal midlobe: punctate rugose anteriorly, sparsely punctate posteriorly. Macrosculpture of lateral lobe of mesoscutum: sparsely punctate. Sculpture of notaulus: smooth. Median mesoscutal carina: present anteriorly, not extending to posterior margin of mesoscutum. Mesoscutellum in lateral view: convex. Sculpture of mesoscutellum: densely punctate rugose. Shape of metascutellum: trapezoidal with broad posterior margin. Median metascutellar carina: present. Sculpture of metascutellum: rugose. Sculpture of lateral propodeal area: smooth. Mesopleural carina: present. Sculpture of mesepisternum below femoral depression: striate to densely punctate below mesopleural carina, otherwise smooth. Setation of dorsal metapleural area: absent. Setation of area directly dorsal to the metapleural triangle: absent. Sculpture of ventral metapleural area: rugose anteriorly, smooth posteriorly. Color of legs: orange yellow throughout. Length of hind basitarsus: about as long as remaining segments combined. Sculpture of hind coxa: largely smooth, with sparse fine punctures.

Length of postmarginal vein: approximately as long as stigmal vein.

Color of metasoma in female: black. Horn on T1 in female: absent. Striae of posterior margin of T1 in female: dense. Striae of T1 in male: dense. Transverse sulcus on T2: absent; present. Sculpture of T2: densely longitudinally striate, punctate rugulose in interstices. Sculpture of T6 in female: longitudinally punctate rugose. Length of T6 in female: approximately as long as wide. Shape of T6 in female in lateral view: sinuate. Apical spine on female T6: present. Sculpture of T6 in male: densely punctate. Sculpture of T7 in male: densely punctate. Posterior margin of T7 in male: straight. Sculpture of medial S2: densely longitudinally striate with fine punctures in interstices.

**Figures 106–111.**
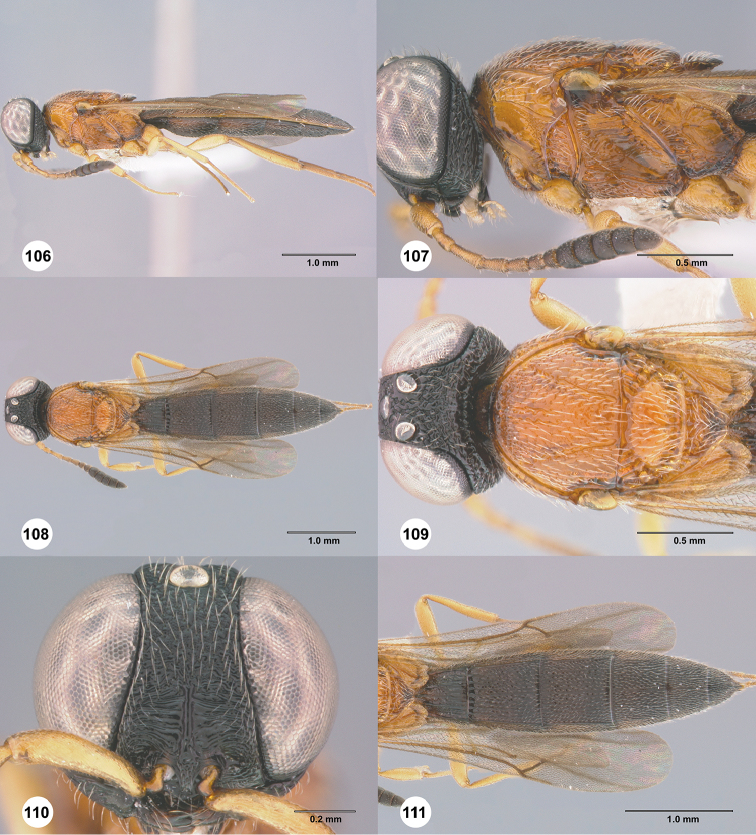
*Chromoteleiafeng* sp. n., female, holotype (OSUC233344). **106** Lateral habitus **107** Head and mesosoma, lateral view **108** Dorsal habitus **109** Head and mesosoma, dorsal view **110** Head, anterior view **111** Metasoma, dorsal view.

#### Diagnosis.

This species is similar to *C.robusta* in the acute T6 apex, but it can be distinguished by the combination of the following characters: female A12 has only one basiconic sensillum, occipital carina complete, notaulus smooth.

#### Etymology.

The epithet is inspired by the Chinese word 锋 (fēng) for the name of the sharp point of a spear, in reference to the pointed female T6, and is intended to be treated as a noun.

#### Link to distribution map.

[http://hol.osu.edu/map-large.html?id=318239]

#### Material examined.

Holotype, female: **BRAZIL**: PA, Melgaço Mpio., Ferreira Penna Scientific Station, trail, P05057, 01°44'28"S, 51°27'21.3"W, Caxiuanã, 16.XI–19.XI.2003, yellow pan trap, A. P. Aguiar & J. Dias, OSUC233344 (deposited in MPEG). *Paratypes*: (24 females, 92 males) **BRAZIL**: 16 females, 90 males, OSUC202483, 202487, 202518, 202520–202521, 202526–202527 (AEIC); OSUC584166–584170, 584176, 586360, 586363–586364, 586366–586367, 586370, 586372 (CNCI); OSUC185794, 185832, 185834–185835, 185837, 185839, 185841, 225228–225229, 225233, 231866, 231868, 231870, 231872–231873, 231886, 241252, 241254, 241256, 241279, 322168, 322170, 322172, 322176, 322594, 322596, 322600, 326258, 326260, 359052, 359058, 359066, 359078, 380236–380237, 380240, 449170, 449174, 449177 (MPEG); OSUC166091, 185792, 185795, 185833, 185836, 185838, 185840, 225222, 225226, 225231, 225238, 231867, 231869, 231871, 231874, 233120, 233293, 241251, 241255, 241257, 254999, 322167, 322169, 322174, 322202, 322595, 322597, 322640, 326259, 359054–359055, 359060, 359070, 359074, 366730, 371944, 380235, 380238, 380241, 381993, 449166, 449173, 449175–449176, 449182 (OSUC); OSUC225301, 225310 (USNM). **FRENCH GUIANA**: 7 females, 2 males, OSUC555821, 586223–586224, 586226–586227, 586231, 586829, 586852, 586858 (CNCI). **VENEZUELA**: 1 female, OSUC557019 (CNCI).

### Chromoteleia fossa

Taxon classificationAnimaliaHymenopteraScelionidae

Chen & Johnson sp. n.

http://zoobank.org/06CBBAC3-7C54-4A69-9395-ED4B55E67D7D

http://bioguid.osu.edu/xbiod_concepts/452226

Figures 112–117

#### Description.

Body length of female: 6.90 mm (n = 1). Color of A1: yellow to orange. A6 in female: as wide as long. A5 in female: distinctly longer than wide. Number of basiconic sensilla on A7: 1. Number of basiconic sensilla on A12: 1. Sculpture of dorsal A1: punctate; smooth. Color of head: black. Sculpture of frons directly above interantennal process: punctate rugose. Central keel: present only in ventral portion of frons. Ventral margin of clypeus: straight. Granulate microsculpture of dorsal frons: absent. Occipital carina: interrupted medially. Granulate microsculpture of vertex: absent. Sculpture of occiput: smooth. Sculpture of gena: dorsoventrally strigose; narrowly smooth ventrally, punctate rugose dorsally.

Color of mesosoma: orange. Sculpture of epicoxal lobe posterior of propleural epicoxal sulcus: densely punctate. Sculpture of lateral pronotal area above pronotal cervical sulcus: smooth dorsally, rugose ventrally. Sculpture of netrion: transversely striate. Microsculpture of mesoscutum: granulate. Macrosculpture of mesoscutal midlobe: punctate rugose anteriorly, sparsely punctate posteriorly. Macrosculpture of lateral lobe of mesoscutum: punctate rugose. Sculpture of notaulus: smooth. Median mesoscutal carina: present anteriorly, not extending to posterior margin of mesoscutum. Mesoscutellum in lateral view: convex. Sculpture of mesoscutellum: smooth medially, densely punctate laterally. Shape of metascutellum: trapezoidal with broad posterior margin. Median metascutellar carina: present. Sculpture of metascutellum: rugose. Sculpture of lateral propodeal area: rugose. Mesopleural carina: present. Sculpture of mesepisternum below femoral depression: striate to densely punctate below mesopleural carina, otherwise smooth. Sculpture of dorsal metapleural area: rugose. Setation of dorsal metapleural area: absent. Setation of area directly dorsal to the metapleural triangle: present. Sculpture of ventral metapleural area: rugose throughout. Color of legs: orange yellow throughout. Length of hind basitarsus: distinctly longer than remaining segments combined. Sculpture of hind coxa: largely smooth, with sparse fine punctures.

Length of postmarginal vein: approximately as long as stigmal vein.

Color of metasoma in female: black. Horn on T1 in female: present. Striae of posterior margin of T1 in female: dense. Transverse sulcus on T2: present. Sculpture of T2: densely longitudinally striate, punctate rugulose in interstices. Sculpture of T6 in female: densely longitudinally striate, with fine punctures in interstices. Length of T6 in female: at least 1.5× longer than wide. Shape of T6 in female in lateral view: flat. Apical spine on female T6: absent. Sculpture of medial S2: densely longitudinally striate with fine punctures in interstices.

**Figures 112–117.**
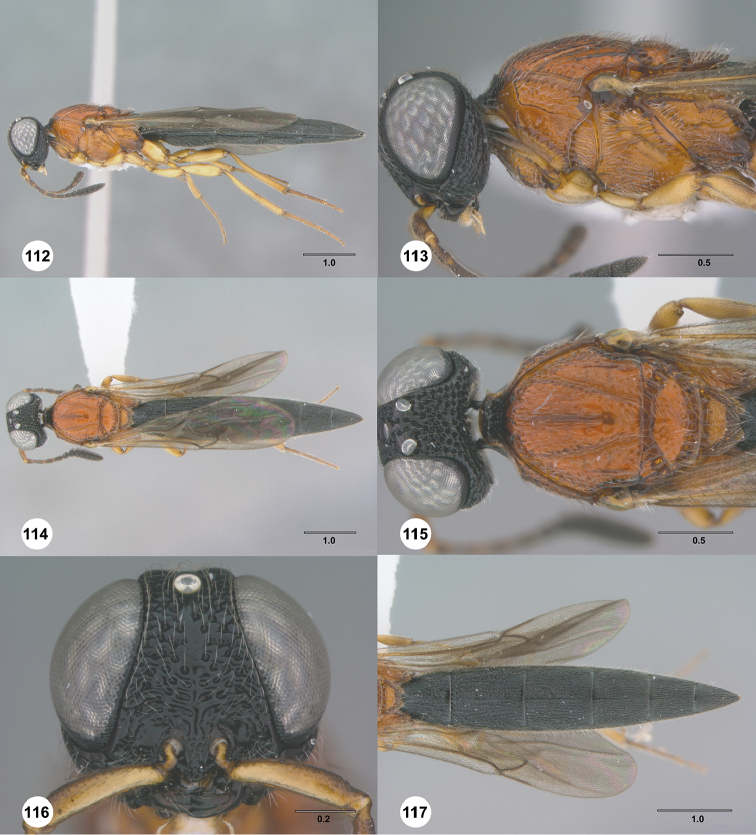
*Chromoteleiafossa* sp. n., female, holotype (OSUC320582). **112** Lateral habitus **113** Head and mesosoma, lateral view **114** Dorsal habitus **115** Head and mesosoma, dorsal view **116** Head, anterior view **117** Metasoma, dorsal view.

#### Diagnosis.

This species is similar to *C.feng* with which it shares the characters of smooth notaulus and female A12 with one basiconic sensillum, but it can be distinguished by the combination of the following characters: female T6 without apical spine, occipital carina interrupted medially.

#### Etymology.

The epithet is inspired by the Latin word for ditch, in reference to the smooth notaulus, and is intended to be treated as a noun.

#### Link to distribution map.

[http://hol.osu.edu/map-large.html?id=452226]

#### Material examined.

Holotype, female: **PANAMA**: Kuna Yala Comarca, 2001/038, 390 m, 09°20'31"N, 78°59'38"W, Nusagandi Station, 12.I–15.I.2001, yellow pan trap, M. Yoder, OSUC320582 (deposited in TAMU).

### Chromoteleia fuscicornis

Taxon classificationAnimaliaHymenopteraScelionidae

Kieffer

http://zoobank.org/FFE02947-921E-4224-971E-7C0259A61634

http://bioguid.osu.edu/xbiod_concepts/4213

Figures 22 , 23 , 25 , 118–121 , 122–127

Chromoteleia fuscicornis Kieffer, 1910a: 313, 316 (original description, keyed); Masner, 1976: 25 (description, type information); Johnson, 1992: 364 (cataloged, type information).
Petalosema fuscicornis (Kieffer): Kieffer, 1926: 358, 360 (generic transfer, description, keyed).

#### Description.

Body length of female: 4.28–6.90 mm (n = 20). Body length of male: 4.10–6.53 mm (n = 20). Color of A1: yellow to orange. A6 in female: distinctly wider than long. A5 in female: distinctly longer than wide; as long as wide. A6 in male: as long as wide. Number of basiconic sensilla on A7: 1. Number of basiconic sensilla on A12: 1. Sculpture of dorsal A1: punctate; smooth. Color of head: black. Sculpture of frons directly above interantennal process: areolate. Central keel: complete, extending from interantennal process to median ocellus. Ventral margin of clypeus: pointed. Granulate microsculpture of dorsal frons: absent. Occipital carina: interrupted medially. Granulate microsculpture of vertex: absent. Sculpture of occiput: smooth medially, striate laterally. Sculpture of gena: narrowly smooth ventrally, punctate rugose dorsally.

Color of mesosoma: variably orange to black. Sculpture of epicoxal lobe posterior of propleural epicoxal sulcus: sparsely punctate. Sculpture of lateral pronotal area above pronotal cervical sulcus: smooth dorsally, rugose ventrally. Sculpture of netrion: transversely striate. Microsculpture of mesoscutum: granulate. Macrosculpture of mesoscutal midlobe: sparsely punctate. Macrosculpture of lateral lobe of mesoscutum: sparsely punctate. Sculpture of notaulus: foveate. Notaular foveae: interconnected. Median mesoscutal carina: present anteriorly, not extending to posterior margin of mesoscutum. Mesoscutellum in lateral view: convex. Sculpture of mesoscutellum: smooth medially, densely punctate laterally. Shape of metascutellum: trapezoidal with broad posterior margin. Median metascutellar carina: present. Sculpture of metascutellum: rugose. Sculpture of lateral propodeal area: smooth. Mesopleural carina: present. Sculpture of mesepisternum below femoral depression: punctate rugose. Sculpture of dorsal metapleural area: rugose. Setation of dorsal metapleural area: present. Setation of area directly dorsal to the metapleural triangle: present. Sculpture of ventral metapleural area: rugose throughout. Color of legs: orange to pale brown. Length of hind basitarsus: distinctly longer than remaining segments combined. Sculpture of hind coxa: densely punctate.

Length of postmarginal vein: distinctly shorter than stigmal vein.

Color of metasoma in female: black. Color of metasoma in male: black. Horn on T1 in female: present. Striae of posterior margin of T1 in female: dense. Striae of T1 in male: sparse. Transverse sulcus on T2: absent. Sculpture of T2: densely longitudinally striate, punctate rugulose in interstices. Sculpture of T6 in female: densely longitudinally striate, with fine punctures in interstices. Length of T6 in female: at least 1.5× longer than wide. Shape of T6 in female in lateral view: sinuate. Apical spine on female T6: absent. Sculpture of T6 in male: densely longitudinally striate with fine punctures in interstices. Sculpture of T7 in male: smooth anteriorly, rugulose posteriorly. Posterior margin of T7 in male: emarginate medially between rounded projections. Sculpture of medial S2: densely longitudinally striate with fine punctures in interstices.

**Figures 118–121.**
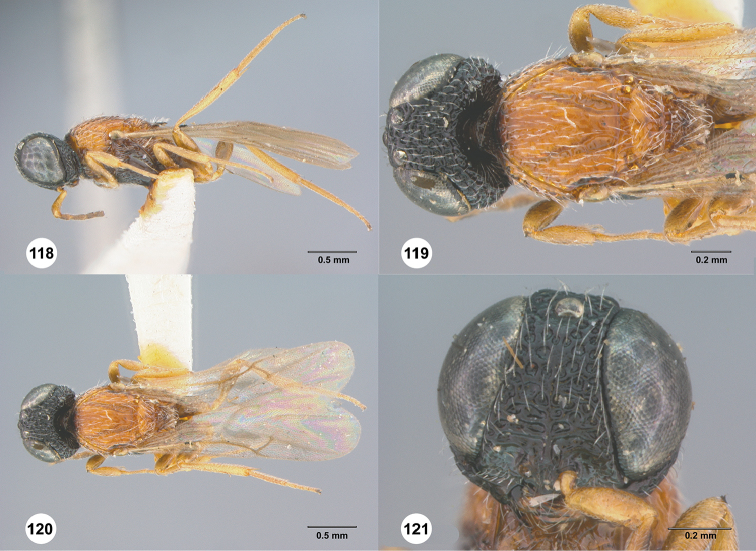
*Chromoteleiafuscicornis* Kieffer, female, holotype (CAS TYPE9649). **118** Lateral habitus **119** Head and mesosoma, lateral view **120** Dorsal habitus **121** Head, anterior view.

**Figures 122–127.**
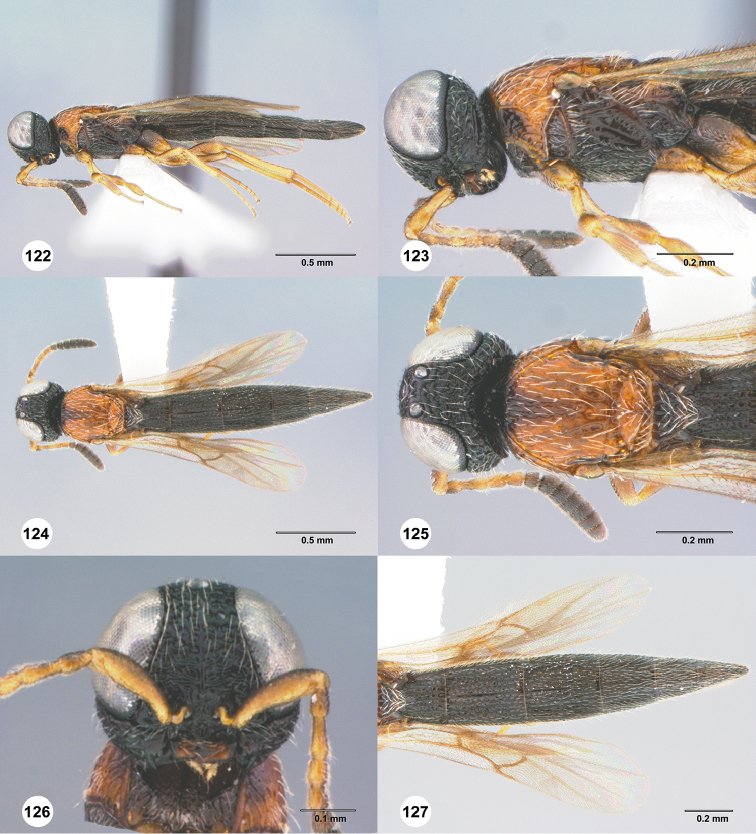
*Chromoteleiafuscicornis* Kieffer, female (OSUC584322). **122** Lateral habitus **123** Head and mesosoma, lateral view **124** Dorsal habitus **125** Head and mesosoma, dorsal view **126** Head, anterior view **127** Metasoma, dorsal view.

#### Diagnosis.

This species can be distinguished from other *Chromoteleia* by the following combination of characters: female A7 with one basiconic sensillum, dorsal metapleural area with setae, basitarsus distinctly longer than remaining segments combined, postmarginal vein distinctly shorter than stigmal vein, male T7 with posterior margin deeply emarginate medially between rounded projections.

#### Link to distribution map.

[http://hol.osu.edu/map-large.html?id=4213]

#### Material examined.

Holotype, female: **BRAZIL**: PA, no date, Baker, CAS TYPE9649 (deposited in CAS).

**Other material**: (164 females, 155 males) **BELIZE**: 1 female, OSUC577461 (CNCI). **BOLIVIA**: 2 females, OSUC586776–586777 (CNCI). **BRAZIL**: 45 females, 78 males, OSUC149647, 202479–202481, 202484, 202486, 202489, 202491, 202494 (AEIC); OSUC577932, 583469, 583472, 583494, 583497, 583503, 584147–584148, 584150–584151, 584154–584156, 584160, 584324, 584327–584329, 584335, 586129, 586133, 586147, 586303, 586314–586315, 586329, 586369, 586371, 586393, 586400–586403, 586416–586417, 586573, 586788–586795 (CNCI); OSUC199636 (FSCA); OSUC185793, 185831, 225230, 225587, 251722, 322601, 326463, 326475, 344379, 354649, 354653, 359053, 359071, 359075, 359077, 379217, 380234, 449167, 449171, 449185 (MPEG); OSUC185796, 185842, 225224, 225234, 225585, 241249, 322171, 326261, 326464, 326476–326477, 344338, 344377, 354577, 354671, 354679, 359061–359062, 359069, 359072–359073, 366731, 374856, 380239, 380244, 449169, 449180, 449183, 56224, 56226, 56244–56247, 56249, 56251–56256, 56259 (OSUC); OSUC204890, 204906–204907, 204988, 204992 (UCDC); OSUC225316–225317 (USNM). **COLOMBIA**: 23 females, 11 males, OSUC586306, 586346, 586762 (CNCI); OSUC143900, 151956, 166454–166455, 166524, 189042, 189623, 190775, 191044, 230435, 232722, 273445, 320929 (IAVH); OSUC143893, 151955, 166452, 166519, 166523, 166541, 170415, 182584, 190079, 190816, 191293, 193459, 232725, 249927, 267217, 269374, 269376, 321675 (OSUC). COSTA RICA: 3 females, OSUC556987, 557081, 586411 (CNCI). **ECUADOR**: 61 females, 38 males, OSUC149650 (AEIC); OSUC556943, 557031, 557033, 557035, 557038–557039, 557090, 557095, 583706, 583708, 583753, 584060, 584098, 584102–584103, 584112, 584114–584117, 584119, 584131, 584133, 584398, 584400, 584404, 584407, 584409, 584414, 584428–584429, 584431, 584759–584762, 585099–585105, 585107–585108, 585110–585118, 585120, 586159–586160, 586177, 586316, 586330, 586334, 586336, 586342, 586412, 586502–586503, 586505, 586554, 586667, 586694–586695, 586708, 586714, 586724, 586731 (CNCI); DPI_FSCA 00010220 (CSCA); OSUC199630 (FSCA); OSUC557032, 557041, 557089, 557097, 584083, 584097, 584104, 584113, 584118, 584402, 584410, 585109, 585119, 586331, 586333, 586531, 586727 (OSUC); OSUC221926–221927 (TAMU); OSUC204994, 204997 (UCDC). **FRENCH GUIANA**: 6 females, 4 males, OSUC586233, 586243, 586246, 586256, 586433, 586828, 586849, 586857, 586862, 586864 (CNCI). **PERU**: 18 females, 24 males, OSUC556970, 578055, 578061, 584309, 584311–584313, 584318–584319, 584321–584322, 586138, 586189, 586440, 586572, 586797–586801, 586803–586804, 586866 (CNCI); DPI_FSCA 00010216–00010219, 00010221–00010224 (CSCA); OSUC199609 (FSCA); DPI_FSCA 00010225–00010226, SM0267120 (KUNH); OSUC181564, 323995, 584320, 586802, 586807 (OSUC); OSUC204984–204985 (UCDC). **SURINAME**: 2 females, OSUC557085, 584297 (CNCI). **VENEZUELA**: 3 females, OSUC586157, 586470, 586472 (CNCI).

### Chromoteleia ingens

Taxon classificationAnimaliaHymenopteraScelionidae

Chen & Masner sp. n.

http://zoobank.org/0CF0DEE6-E009-4EAE-9723-FC456357CBFC

http://bioguid.osu.edu/xbiod_concepts/452212

Figures 9 , 128–133

#### Description.

Body length of female: 8.78–9.20 mm (n = 6). Body length of male: 7.60–7.83 mm (n = 4). Color of A1: yellow to orange. A6 in female: distinctly longer than wide. A5 in female: distinctly longer than wide. A6 in male: approximately 2.0× longer than wide. Number of basiconic sensilla on A7: 0. Number of basiconic sensilla on A12: 2. Sculpture of dorsal A1: smooth.

Color of head: black. Sculpture of frons directly above interantennal process: punctate rugose. Central keel: present, interrupted medially. Ventral margin of clypeus: pointed. Granulate microsculpture of dorsal frons: absent. Occipital carina: complete. Granulate microsculpture of vertex: absent. Sculpture of occiput: smooth. Sculpture of gena: dorsoventrally strigose.

Color of mesosoma: variably orange to black. Sculpture of epicoxal lobe posterior of propleural epicoxal sulcus: densely punctate. Sculpture of lateral pronotal area above pronotal cervical sulcus: smooth throughout. Sculpture of netrion: transversely striate. Microsculpture of mesoscutum: absent. Macrosculpture of mesoscutal midlobe: sparsely punctate. Macrosculpture of lateral lobe of mesoscutum: punctate rugose. Sculpture of notaulus: foveate. Notaular foveae: interconnected. Median mesoscutal carina: present anteriorly, not extending to posterior margin of mesoscutum. Mesoscutellum in lateral view: convex. Sculpture of mesoscutellum: smooth medially, densely punctate laterally. Shape of metascutellum: trapezoidal with broad posterior margin. Median metascutellar carina: present. Sculpture of metascutellum: rugose. Sculpture of lateral propodeal area: rugose. Mesopleural carina: present. Sculpture of mesepisternum below femoral depression: punctate rugose. Sculpture of dorsal metapleural area: rugose. Setation of dorsal metapleural area: present. Setation of area directly dorsal to the metapleural triangle: absent. Sculpture of ventral metapleural area: rugose anteriorly, smooth posteriorly. Color of legs: orange yellow to brown, with tarsi darker. Length of hind basitarsus: distinctly longer than remaining segments combined. Sculpture of hind coxa: largely smooth, with sparse fine punctures.

Length of postmarginal vein: distinctly longer than stigmal vein.

Color of metasoma in female: black. Color of metasoma in male: black. Horn on T1 in female: present. Striae of posterior margin of T1 in female: dense. Striae of T1 in male: sparse. Transverse sulcus on T2: present. Sculpture of T2: longitudinally punctate rugose. Sculpture of T6 in female: densely longitudinally striate, with fine punctures in interstices. Length of T6 in female: at least 1.5× longer than wide. Shape of T6 in female in lateral view: flat. Apical spine on female T6: absent. Sculpture of T6 in male: densely longitudinally striate with fine punctures in interstices. Sculpture of T7 in male: smooth to coriaceous. Posterior margin of T7 in male: emarginate medially between rounded projections. Sculpture of medial S2: densely longitudinally striate with fine punctures in interstices.

**Figures 128–133.**
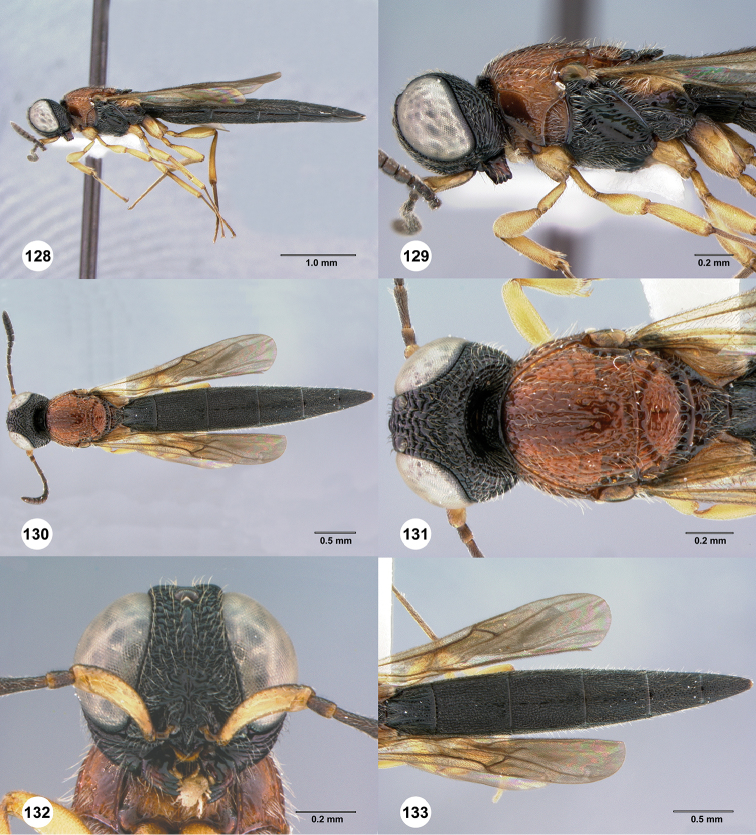
*Chromoteleiaingens* sp. n., female, holotype (OSUC577500). **128** Lateral habitus **129** Head and mesosoma, lateral view **130** Dorsal habitus **131** Head and mesosoma, dorsal view **132** Head, anterior view **133** Metasoma, dorsal view.

#### Diagnosis.

This species is similar to *C.longitarsis*, but it can be distinguished by the combination of the following characters: female A12 has two basiconic sensilla, and occipital carina complete.

#### Etymology.

The epithet is inspired by the Latin word for huge, in reference to the large body size of this species, and is intended to be treated as an adjective.

#### Link to distribution map.

[http://hol.osu.edu/map-large.html?id=452212]

#### Material examined.

Holotype, female: **ECUADOR**: Orellana Prov., Yasuní Scientific Research Station (YSRS), 220m, 00.67°S 76.39°W, Yasuní National Park, 18.V–27.V.1990, P. Hibbs, OSUC577500 (deposited in CNCI). *Paratypes*: (5 females, 4 males) **BRAZIL**: 4 females, 4 males, OSUC149636, 202473–202478 (AEIC); OSUC583458 (CNCI). **ECUADOR**: 1 female, OSUC577499 (CNCI).

### Chromoteleia levitas

Taxon classificationAnimaliaHymenopteraScelionidae

Chen & Johnson sp. n.

http://zoobank.org/F5087C96-A2E3-420E-B28F-7089D61A20A4

http://bioguid.osu.edu/xbiod_concepts/452159

Figures 134–139

#### Description.

Body length of female: 5.68–6.60 mm (n = 20). Body length of male: 4.60–5.10 mm (n = 8). Color of A1: yellow to orange. A6 in female: as wide as long. A5 in female: distinctly longer than wide. A6 in male: approximately 2.0× longer than wide. Number of basiconic sensilla on A7: 0. Number of basiconic sensilla on A12: 1. Sculpture of dorsal A1: striate. Color of head: black. Sculpture of frons directly above interantennal process: transversely striate to rugose. Central keel: present only in ventral portion of frons. Ventral margin of clypeus: pointed. Granulate microsculpture of dorsal frons: absent. Occipital carina: complete. Granulate microsculpture of vertex: absent. Sculpture of occiput: smooth. Sculpture of gena: punctate rugose dorsally and ventrally, strigose medially.

Color of mesosoma: variably orange to black. Sculpture of epicoxal lobe posterior of propleural epicoxal sulcus: smooth. Sculpture of lateral pronotal area above pronotal cervical sulcus: smooth dorsally, rugose ventrally. Sculpture of netrion: punctate rugose anteriorly, smooth posteriorly. Microsculpture of mesoscutum: granulate. Macrosculpture of mesoscutal midlobe: punctate rugose throughout. Macrosculpture of lateral lobe of mesoscutum: punctate rugose. Sculpture of notaulus: smooth. Median mesoscutal carina: present anteriorly, not extending to posterior margin of mesoscutum. Mesoscutellum in lateral view: convex. Sculpture of mesoscutellum: densely punctate rugose. Shape of metascutellum: trapezoidal with broad posterior margin. Median metascutellar carina: present. Sculpture of metascutellum: rugose. Sculpture of lateral propodeal area: rugose. Mesopleural carina: present. Sculpture of mesepisternum below femoral depression: largely smooth, punctate rugose anteriorly and directly below femoral depression. Sculpture of dorsal metapleural area: rugose; smooth. Setation of dorsal metapleural area: absent. Setation of area directly dorsal to the metapleural triangle: absent. Sculpture of ventral metapleural area: rugose throughout; rugose anteriorly, smooth posteriorly. Color of legs: orange yellow throughout. Length of hind basitarsus: about as long as remaining segments combined. Sculpture of hind coxa: densely punctate.

Length of postmarginal vein: approximately as long as stigmal vein.

Color of metasoma in female: black. Color of metasoma in male: black. Horn on T1 in female: present. Striae of posterior margin of T1 in female: dense. Striae of T1 in male: sparse. Transverse sulcus on T2: present. Sculpture of T2: densely longitudinally striate, punctate rugulose in interstices. Sculpture of T6 in female: densely longitudinally striate, with fine punctures in interstices. Length of T6 in female: at least 1.5× longer than wide. Shape of T6 in female in lateral view: flat. Apical spine on female T6: absent. Sculpture of T6 in male: densely punctate. Sculpture of T7 in male: densely punctate. Posterior margin of T7 in male: emarginate medially between rounded projections. Sculpture of medial S2: densely longitudinally striate with fine punctures in interstices.

**Figures 134–139.**
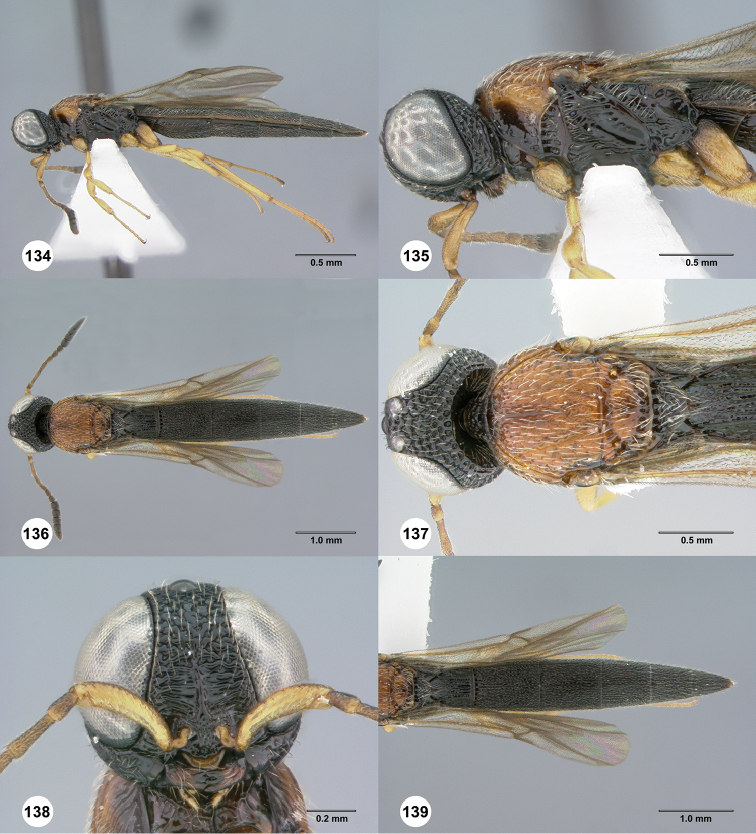
*Chromoteleialevitas* sp. n., female, holotype (OSUC577476). **134** Lateral habitus **135** Head and mesosoma, lateral view **136** Dorsal habitus **137** Head and mesosoma, dorsal view **138** Head, anterior view **139** Metasoma, dorsal view.

#### Diagnosis.

This species is similar to *C.rara*, but it can be distinguished by the combination of the smooth notaulus smooth and striate A1.

#### Etymology.

The epithet is inspired by the Latin word for smoothness, in reference to the mostly smooth metapleuron, and is intended to be treated as a noun.

#### Link to distribution map.

[http://hol.osu.edu/map-large.html?id=452159]

#### Material examined.

Holotype, female: **BRAZIL**: RO, 62 km S Ariquemes, Rancho Grande Farm, 12.XI–22.XI.1991, E. M. Fisher, OSUC577476 (deposited in CNCI). *Paratypes*: (43 females, 8 males) **BRAZIL**: 21 females, 5 males, OSUC149642, 202510 (AEIC); OSUC577471–577474, 577477, 577479–577482, 584157–584158, 584331–584333 (CNCI); OSUC354579, 380243 (MPEG); OSUC577475, 577478, 577483, 584153, 584326 (OSUC); OSUC225307–225308, 225311 (USNM). **COLOMBIA**: 5 females, OSUC143982, 193662 (IAVH); OSUC228672, 269686, 274926 (OSUC). COSTA RICA: 1 female, OSUC584243 (CNCI). **ECUADOR**: 7 females, 2 males, OSUC585091–585096, 585098, 586529 (CNCI); OSUC586530 (OSUC). **FRENCH GUIANA**: 6 females, OSUC555802, 555817, 555820, 555822, 586853 (CNCI); OSUC555823 (OSUC). **PERU**: 2 females, 1 male, OSUC584306, 584317 (CNCI); OSUC320746 (USNM). **SURINAME**: 1 female, OSUC584296 (CNCI). *Other material*: **PERU**: 1 female, 1 male, SEMC0983802, SEMC0985301T (KUNH).

### Chromoteleia longitarsis

Taxon classificationAnimaliaHymenopteraScelionidae

Kieffer

http://zoobank.org/17954F24-A488-4D04-99FE-12F22370E2C1

http://bioguid.osu.edu/xbiod_concepts/4214

Figures 16–17 , 27 , 140–145 , 146–151 , 236

Chromoteleia longitarsis Kieffer, 1910a: 313, 314 (original description, keyed); Masner, 1976: 25 (type information); Johnson, 1992: 364 (cataloged, type information).
Petalosema longitarsis (Kieffer): Kieffer, 1926: 358, 359 (generic transfer, description, keyed).

#### Description.

Body length of female: 6.88–7.20 mm (n = 20). Body length of male: 6.30–6.73 mm (n = 20). Color of A1: yellow to orange. A6 in female: distinctly longer than wide. A5 in female: distinctly longer than wide. A6 in male: approximately 2.0× longer than wide. Number of basiconic sensilla on A7: 0. Number of basiconic sensilla on A12: 1. Sculpture of dorsal A1: striate. Color of head: black. Sculpture of frons directly above interantennal process: transversely striate to rugose. Central keel: complete, extending from interantennal process to median ocellus. Ventral margin of clypeus: straight. Granulate microsculpture of dorsal frons: absent. Occipital carina: interrupted medially. Granulate microsculpture of vertex: absent. Sculpture of occiput: smooth. Sculpture of gena: dorsoventrally strigose.

Color of mesosoma: variably orange to black. Sculpture of epicoxal lobe posterior of propleural epicoxal sulcus: sparsely punctate. Sculpture of lateral pronotal area above pronotal cervical sulcus: smooth throughout. Sculpture of netrion: rugose. Microsculpture of mesoscutum: absent. Macrosculpture of mesoscutal midlobe: punctate rugose throughout. Macrosculpture of lateral lobe of mesoscutum: densely punctate. Sculpture of notaulus: foveate. Notaular foveae: interconnected. Median mesoscutal carina: present anteriorly, not extending to posterior margin of mesoscutum. Mesoscutellum in lateral view: convex. Sculpture of mesoscutellum: densely punctate rugose. Shape of metascutellum: trapezoidal with broad posterior margin. Median metascutellar carina: present. Sculpture of metascutellum: rugose. Sculpture of lateral propodeal area: smooth. Mesopleural carina: present. Sculpture of mesepisternum below femoral depression: punctate rugose. Sculpture of dorsal metapleural area: rugose. Setation of dorsal metapleural area: present. Setation of area directly dorsal to the metapleural triangle: present. Sculpture of ventral metapleural area: rugose throughout. Color of legs: pale yellow with tarsi dark brown to black. Length of hind basitarsus: distinctly longer than remaining segments combined. Sculpture of hind coxa: largely smooth, with sparse fine punctures.

Length of postmarginal vein: distinctly longer than stigmal vein.

Color of metasoma in female: black. Color of metasoma in male: black. Horn on T1 in female: present. Striae of posterior margin of T1 in female: dense. Striae of T1 in male: sparse. Transverse sulcus on T2: absent. Sculpture of T2: densely longitudinally striate, punctate rugulose in interstices. Sculpture of T6 in female: densely longitudinally striate, with fine punctures in interstices. Length of T6 in female: at least 1.5× longer than wide. Shape of T6 in female in lateral view: sinuate. Apical spine on female T6: absent. Sculpture of T6 in male: densely longitudinally striate with fine punctures in interstices. Sculpture of T7 in male: densely punctate. Posterior margin of T7 in male: straight. Sculpture of medial S2: densely longitudinally striate with fine punctures in interstices.

**Figures 140–145.**
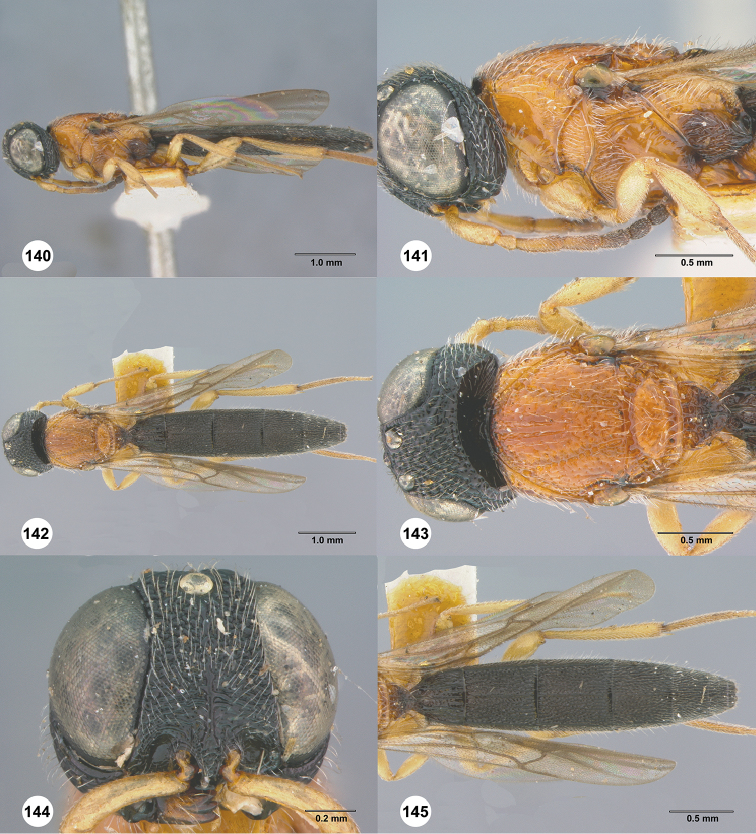
*Chromoteleialongitarsis* Kieffer, male, holotype (CAS TYPE9682). **140** Lateral habitus **141** Head and mesosoma, lateral view **142** Dorsal habitus **143** Head and mesosoma, dorsal view **144** Head, anterior view **145** Metasoma, dorsal view.

**Figures 146–151.**
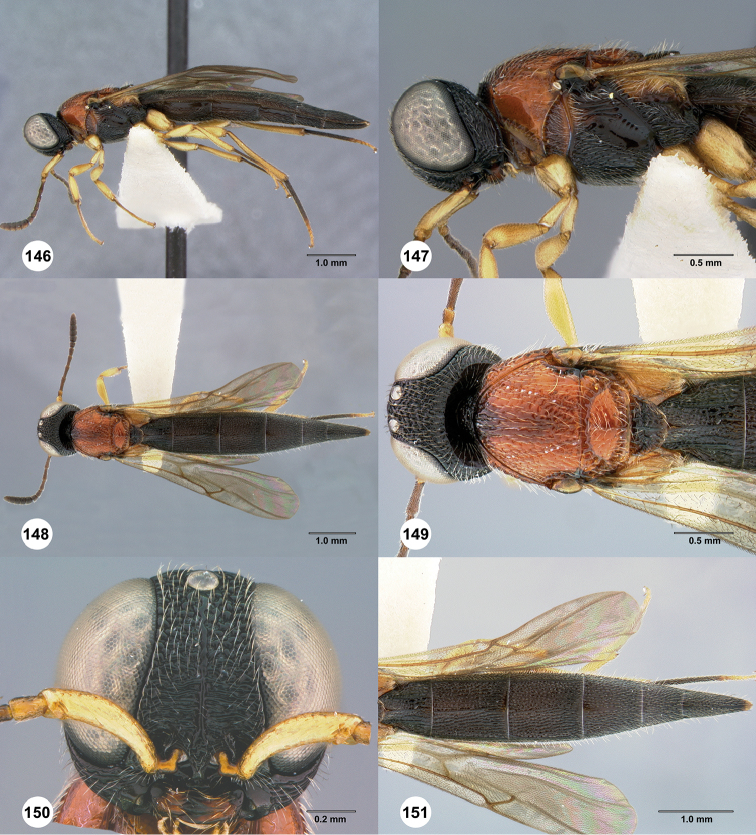
*Chromoteleialongitarsis* Kieffer, female (OSUC584896). **146** Lateral habitus **147** Head and mesosoma, lateral view **148** Dorsal habitus **149** Head and mesosoma, dorsal view **150** Head, anterior view **151** Metasoma, dorsal view.

#### Diagnosis.

This species can be distinguished from other *Chromoteleia* species by the following combination of characters: female A7 without basiconic sensilla, dorsal metapleural area with setae, basitarsus distinctly longer than remaining segments combined, postmarginal vein distinctly longer than stigmal vein, male T7 with posterior margin straight.

#### Link to distribution map.

[http://hol.osu.edu/map-large.html?id=4214]

#### Material examined.

Holotype, male: **BRAZIL**: PA, no date, Baker, CAS TYPE9682 (deposited in CAS).

**Other material**: (84 females, 58 males) **BRAZIL**: 7 females, 6 males, OSUC202525, 202528, 202531 (AEIC); OSUC584895–584896, 584898–584901 (CNCI); OSUC379215 (MPEG); OSUC185791, 56242, 584897 (OSUC). **COLOMBIA**: 2 females, OSUC193428 (IAVH); OSUC253477 (OSUC). **COSTA RICA**: 57 females, 26 males, OSUC583420, 583776, 584260, 584263, 584857–584864, 584866–584868, 584870–584874, 584876–584878, 584880–584887, 584889–584891, 584964–584969, 584971–584976, 584978–584980, 584982–584983, 584985–584986, 584988–584992, 584994–584999, 586751–586752, 586754, 586756, 586838, 586843 (CNCI); OSUC584865, 584869, 584875, 584879, 584888, 584970, 584977, 584981, 584984, 584987, 584993, 585000, 586753 (OSUC). **ECUADOR**: 8 females, 1 male, OSUC584892–584894, 584902, 584911–584912, 584919, 586528 (CNCI); DPI_FSCA 00010210 (CSCA). **FRENCH GUIANA**: 7 females, 11 males, OSUC555803, 555806, 555808, 555810, 555812, 555816, 555818, 555824, 584913, 586239, 586424–586425, 586437, 586861 (CNCI); OSUC555805, 555809, 555815, 586860 (OSUC). **PANAMA**: 2 males, OSUC584380, 586755 (CNCI). **PERU**: 1 female, OSUC578056 (CNCI). **SURINAME**: 1 female, OSUC584903 (CNCI). **TRINIDAD AND TOBAGO**: 1 female, 12 males, OSUC584144, 584904–584910, 584914–584918 (CNCI).

### Chromoteleia longa

Taxon classificationAnimaliaHymenopteraScelionidae

Chen & Johnson sp. n.

http://zoobank.org/1DB03949-CD59-4BC2-AD82-1DCFFA974FEB

http://bioguid.osu.edu/xbiod_concepts/318200

Figures 5–6 , 152–157

#### Description.

Body length of female: 5.28–5.90 mm (n = 20). Body length of male: 5.10–5.83 mm (n = 20). Color of A1: yellow to orange. A6 in female: distinctly longer than wide. A5 in female: distinctly longer than wide. A6 in male: approximately 2.0× longer than wide. Number of basiconic sensilla on A7: 0. Number of basiconic sensilla on A12: 1. Sculpture of dorsal A1: striate. Color of head: black. Sculpture of frons directly above interantennal process: punctate rugose. Central keel: present, interrupted medially. Ventral margin of clypeus: pointed. Granulate microsculpture of dorsal frons: absent. Occipital carina: interrupted medially. Granulate microsculpture of vertex: absent. Sculpture of occiput: smooth. Sculpture of gena: narrowly smooth ventrally, punctate rugose dorsally.

Color of mesosoma: orange. Sculpture of epicoxal lobe posterior of propleural epicoxal sulcus: sparsely punctate. Sculpture of lateral pronotal area above pronotal cervical sulcus: smooth throughout. Sculpture of netrion: transversely striate. Microsculpture of mesoscutum: absent. Macrosculpture of mesoscutal midlobe: punctate rugose throughout. Macrosculpture of lateral lobe of mesoscutum: punctate rugose. Sculpture of notaulus: foveate. Notaular foveae: interconnected. Median mesoscutal carina: present anteriorly, not extending to posterior margin of mesoscutum. Mesoscutellum in lateral view: convex. Sculpture of mesoscutellum: densely punctate rugose. Shape of metascutellum: trapezoidal with broad posterior margin. Median metascutellar carina: present. Sculpture of metascutellum: rugose. Sculpture of lateral propodeal area: rugose. Mesopleural carina: present. Sculpture of mesepisternum below femoral depression: striate to densely punctate below mesopleural carina, otherwise smooth. Sculpture of dorsal metapleural area: rugose; smooth. Setation of dorsal metapleural area: absent. Setation of area directly dorsal to the metapleural triangle: absent. Sculpture of ventral metapleural area: rugose anteriorly, smooth posteriorly. Color of legs: orange yellow throughout. Length of hind basitarsus: about as long as remaining segments combined. Sculpture of hind coxa: densely punctate.

Length of postmarginal vein: distinctly longer than stigmal vein.

Color of metasoma in female: black. Horn on T1 in female: absent. Striae of posterior margin of T1 in female: dense. Striae of T1 in male: dense. Transverse sulcus on T2: present. Sculpture of T2: longitudinally punctate rugose. Sculpture of T6 in female: densely punctate and granulate. Length of T6 in female: approximately as long as wide. Shape of T6 in female in lateral view: flat. Apical spine on female T6: absent. Sculpture of T6 in male: densely punctate. Sculpture of T7 in male: coriaceous anteriorly, densely punctate posteriorly. Posterior margin of T7 in male: emarginate medially between rounded projections. Sculpture of medial S2: densely longitudinally striate with fine punctures in interstices.

**Figures 152–157.**
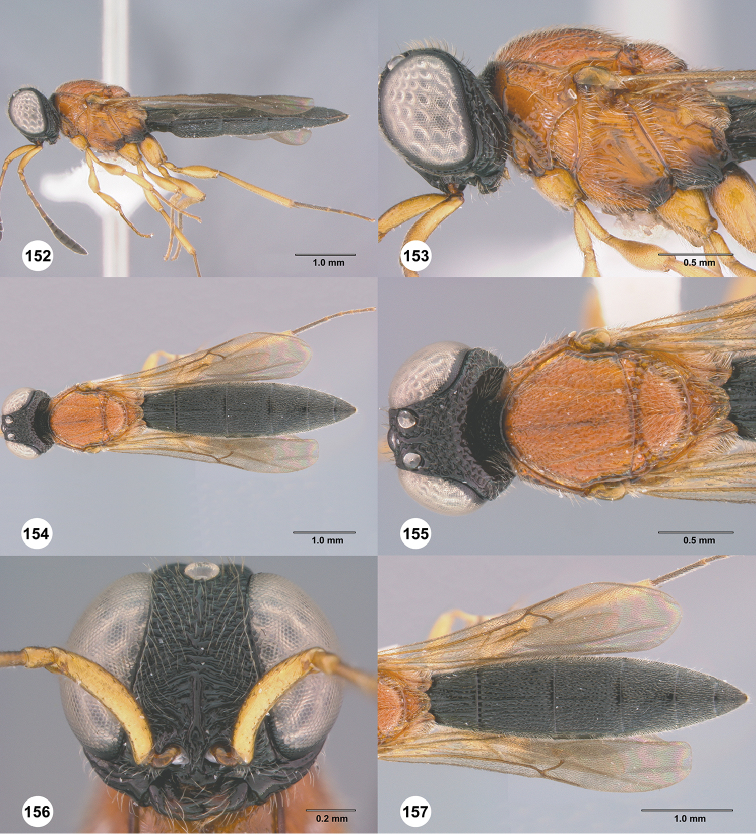
*Chromoteleialonga* sp. n., female, holotype (OSUC188702). **152** Lateral habitus **153** Head and mesosoma, lateral view **154** Dorsal habitus **155** Head and mesosoma, dorsal view **156** Head, anterior view **157** Metasoma, dorsal view.

#### Diagnosis.

This species is similar to *C.tricarinata*, but it can be distinguished by the combination of the following characters: female A6 distinctly longer than wide, female A12 with one basiconic sensillum, median mesoscutal carina present anteriorly.

#### Etymology.

The epithet is inspired by the Latin word for long, in reference to the elongate antenna in female, and is intended to be treated as an adjective.

#### Link to distribution map.

[http://hol.osu.edu/map-large.html?id=318200]

#### Material examined.

*Holotype*, female: **COLOMBIA**: Chocó Dept., visitor’s center, M.815, 2 m, 06°01'N, 77°20'W, Utría Natural National Park, 28.IX–15.X.2000, Malaise trap, J. Pérez, OSUC188702 (deposited in OSUC). *Paratypes*: (49 females, 59 males) **BELIZE**: 14 females, OSUC556959, 556961, 556963–556964, 556967, 556969, 586771, 586869 (CNCI); OSUC556958, 556960, 556962, 556965, 556968, 586867 (OSUC). **COLOMBIA**: 5 females, 11 males, OSUC199631–199632 (FSCA); OSUC144297, 190807–190808, 190819–190821 (IAVH); OSUC144296, 144298, 188703, 190809, 190817–190818, 190822, 269594 (OSUC). **COSTA RICA**: 19 females, 39 males, OSUC577985, 578006, 578008, 578071, 583424–583428, 583430–583433, 583435, 583438, 583442, 583445–583447, 583450–583452, 583454–583457, 583716, 583765, 583784–583785, 583799–583813, 583816, 583976, 584229, 584252, 586392, 586450 (CNCI); OSUC583439, 583441, 583443–583444, 583453, 583814–583815 (OSUC). **FRENCH GUIANA**: 1 male, OSUC586438 (CNCI). **PANAMA**: 11 females, 8 males, OSUC556948–556949, 556956, 584178, 584374, 584377, 586176, 586376, 586622, 586677–586678, 586818 (CNCI); OSUC320583, 556952, 586621 (OSUC); OSUC221924, 320584, 320641, 320643 (TAMU).

### Chromoteleia maura

Taxon classificationAnimaliaHymenopteraScelionidae

Chen & Masner sp. n.

http://zoobank.org/0C29F53A-F881-4B25-9AA7-D2ABB704F172

http://bioguid.osu.edu/xbiod_concepts/452214

Figures 158–163

#### Description.

Body length of female: 5.20 mm (n = 1). Color of A1: black. A6 in female: distinctly longer than wide. A5 in female: distinctly longer than wide. Number of basiconic sensilla on A7: 1. Number of basiconic sensilla on A12: 1. Sculpture of dorsal A1: striate. Color of head: black. Sculpture of frons directly above interantennal process: punctate rugose. Central keel: present, interrupted medially. Ventral margin of clypeus: pointed. Granulate microsculpture of dorsal frons: absent. Occipital carina: complete. Granulate microsculpture of vertex: absent. Sculpture of occiput: smooth. Sculpture of gena: dorsoventrally strigose.

Color of mesosoma: black. Sculpture of epicoxal lobe posterior of propleural epicoxal sulcus: smooth. Sculpture of lateral pronotal area above pronotal cervical sulcus: smooth throughout. Sculpture of netrion: transversely striate. Microsculpture of mesoscutum: absent. Macrosculpture of mesoscutal midlobe: densely punctate. Macrosculpture of lateral lobe of mesoscutum: densely punctate. Sculpture of notaulus: smooth. Median mesoscutal carina: present along full length of mesoscutum. Mesoscutellum in lateral view: convex. Sculpture of mesoscutellum: densely punctate rugose. Shape of metascutellum: trapezoidal with broad posterior margin. Median metascutellar carina: absent or indistinguishable from sculpture. Sculpture of metascutellum: rugose. Sculpture of lateral propodeal area: rugose. Mesopleural carina: present. Sculpture of mesepisternum below femoral depression: rugose anteriorly, sparsely punctate posteriorly. Sculpture of dorsal metapleural area: rugose. Setation of dorsal metapleural area: present. Setation of area directly dorsal to the metapleural triangle: absent. Sculpture of ventral metapleural area: rugose throughout. Color of legs: coxae and trochanters orange yellow, remainder of the legs dark brown. Length of hind basitarsus: about as long as remaining segments combined. Sculpture of hind coxa: largely smooth, with sparse fine punctures.

Length of postmarginal vein: distinctly shorter than stigmal vein.

Color of metasoma in female: black. Horn on T1 in female: absent. Striae of posterior margin of T1 in female: dense. Transverse sulcus on T2: absent. Sculpture of T2: densely longitudinally striate, punctate rugulose in interstices. Sculpture of T6 in female: longitudinally punctate rugose. Length of T6 in female: approximately as long as wide. Shape of T6 in female in lateral view: sinuate. Apical spine on female T6: present. Sculpture of medial S2: densely longitudinally striate with fine punctures in interstices.

**Figures 158–163.**
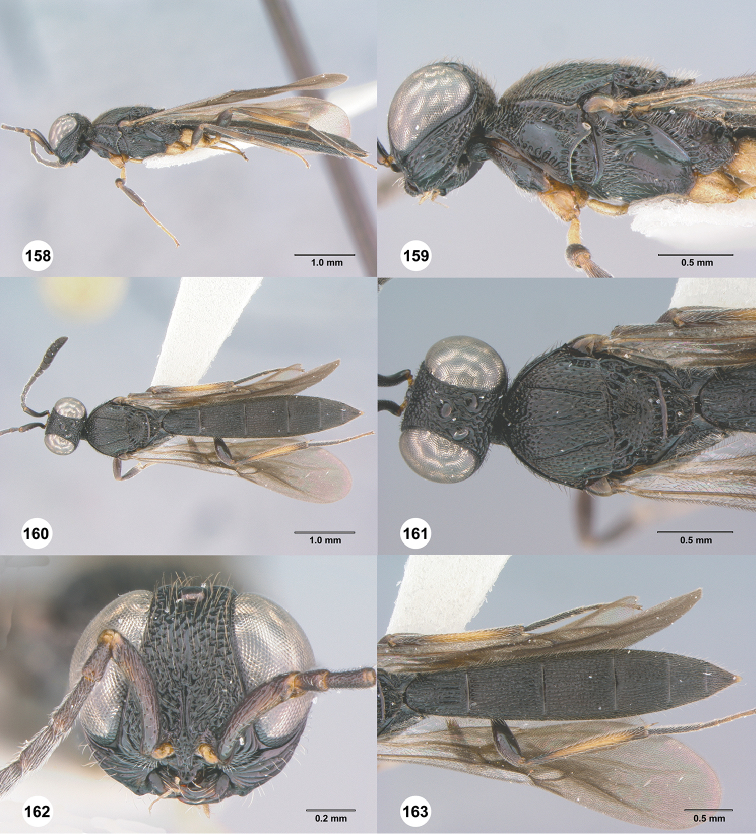
*Chromoteleiamaura* sp. n., female, holotype (OSUC584757). **158** Lateral habitus **159** Head and mesosoma, lateral view **160** Dorsal habitus **161** Head and mesosoma, dorsal view **162** Head, anterior view **163** Metasoma, dorsal view.

#### Diagnosis.

This species can be easily distinguished by its black body, mostly dark brown legs, and smooth notaulus.

#### Etymology.

The epithet is inspired by the Latin word for dark, in reference to the black body and legs, and is intended to be treated as an adjective.

#### Link to distribution map.

[http://hol.osu.edu/map-large.html?id=452214]

#### Material examined.

Holotype, female: **ECUADOR**: Napo Prov., 5 km S Baeza, 1700 m, 13.II.1983, Masner and Sharkey OSUC584757 (deposited in CNCI).

### Chromoteleia parvitas

Taxon classificationAnimaliaHymenopteraScelionidae

Chen & Johnson sp. n.

http://zoobank.org/0EC98684-75B3-4609-BB53-9C64BC234AC9

http://bioguid.osu.edu/xbiod_concepts/452227

Figures 3–4 , 164–169

#### Description.

Body length of female: 3.38–3.65 mm (n = 5). Color of A1: yellow to orange. A6 in female: distinctly wider than long. A5 in female: as long as wide. Number of basiconic sensilla on A7: 1. Number of basiconic sensilla on A12: 1. Sculpture of dorsal A1: smooth. Color of head: black. Sculpture of frons directly above interantennal process: areolate. Central keel: complete, extending from interantennal process to median ocellus. Ventral margin of clypeus: pointed. Granulate microsculpture of dorsal frons: absent. Occipital carina: complete. Granulate microsculpture of vertex: absent. Sculpture of occiput: smooth medially, striate laterally. Sculpture of gena: coarsely punctate rugose.

Color of mesosoma: variably orange to black. Sculpture of epicoxal lobe posterior of propleural epicoxal sulcus: smooth. Sculpture of lateral pronotal area above pronotal cervical sulcus: smooth dorsally, rugose ventrally. Sculpture of netrion: transversely striate. Microsculpture of mesoscutum: granulate. Macrosculpture of mesoscutal midlobe: punctate rugose anteriorly, sparsely punctate posteriorly. Macrosculpture of lateral lobe of mesoscutum: sparsely punctate. Sculpture of notaulus: foveate. Notaular foveae: interconnected. Median mesoscutal carina: present anteriorly, not extending to posterior margin of mesoscutum. Mesoscutellum in lateral view: flat. Sculpture of mesoscutellum: smooth medially, densely punctate laterally. Shape of metascutellum: trapezoidal with broad posterior margin. Median metascutellar carina: present. Sculpture of metascutellum: rugose. Sculpture of lateral propodeal area: rugose. Sculpture of mesepisternum below femoral depression: punctate rugose. Setation of dorsal metapleural area: absent. Setation of area directly dorsal to the metapleural triangle: present. Sculpture of ventral metapleural area: rugose throughout. Color of legs: orange yellow throughout. Length of hind basitarsus: about as long as remaining segments combined. Sculpture of hind coxa: densely punctate.

Length of postmarginal vein: distinctly shorter than stigmal vein.

Color of metasoma in female: black. Horn on T1 in female: absent. Striae of posterior margin of T1 in female: dense. Transverse sulcus on T2: present. Sculpture of T2: densely longitudinally striate, punctate rugulose in interstices. Sculpture of T6 in female: longitudinally punctate rugose. Length of T6 in female: approximately as long as wide. Shape of T6 in female in lateral view: flat. Apical spine on female T6: absent. Sculpture of medial S2: densely longitudinally striate with fine punctures in interstices.

**Figures 164–169.**
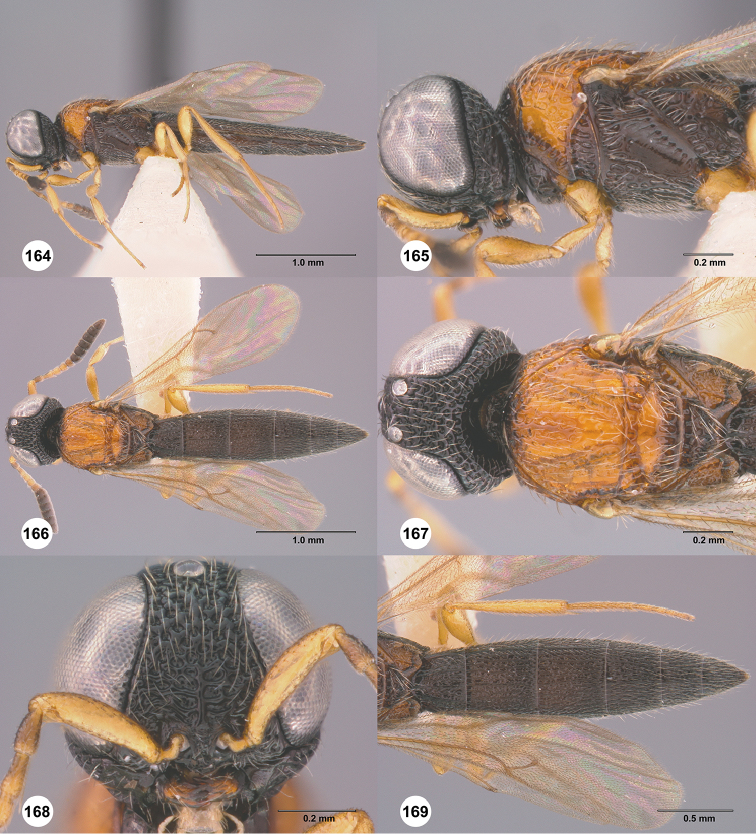
*Chromoteleiaparvitas* sp. n., female, holotype (OSUC586341). **164** Lateral habitus **165** Head and mesosoma, lateral view **166** Dorsal habitus **167** Head and mesosoma, dorsal view **168** Head, anterior view **169** Metasoma, dorsal view.

#### Diagnosis.

This species is similar to *C.curta*, but it can be distinguished by the combination of the following characters: A5 in female as long as wide, postmarginal vein distinctly shorter than stigmal vein, female T1 horn absent.

#### Etymology.

The epithet is inspired by the Latin word for small, in reference to the small body size of this species, and is intended to be treated as a noun.

#### Link to distribution map.

[http://hol.osu.edu/map-large.html?id=452227]

#### Material examined.

Holotype, female: **ECUADOR**: Sucumbíos Prov., Limoncocha, 250 m, 15.VI–28.VI.1976, S. Peck & J. Peck, OSUC586341 (deposited in CNCI). *Paratypes*: (4 females) **BRAZIL**: 1 female, OSUC584163 (CNCI). **COLOMBIA**: 2 females, OSUC584303 (IAVH); OSUC276182 (OSUC). **ECUADOR**: 1 female, OSUC583752 (CNCI).

### Chromoteleia pilus

Taxon classificationAnimaliaHymenopteraScelionidae

Chen & Johnson sp. n.

http://zoobank.org/6A3CC03A-FB31-4C4B-8BD3-87C2F935D18C

http://bioguid.osu.edu/xbiod_concepts/452225

**Figures 170–175.**
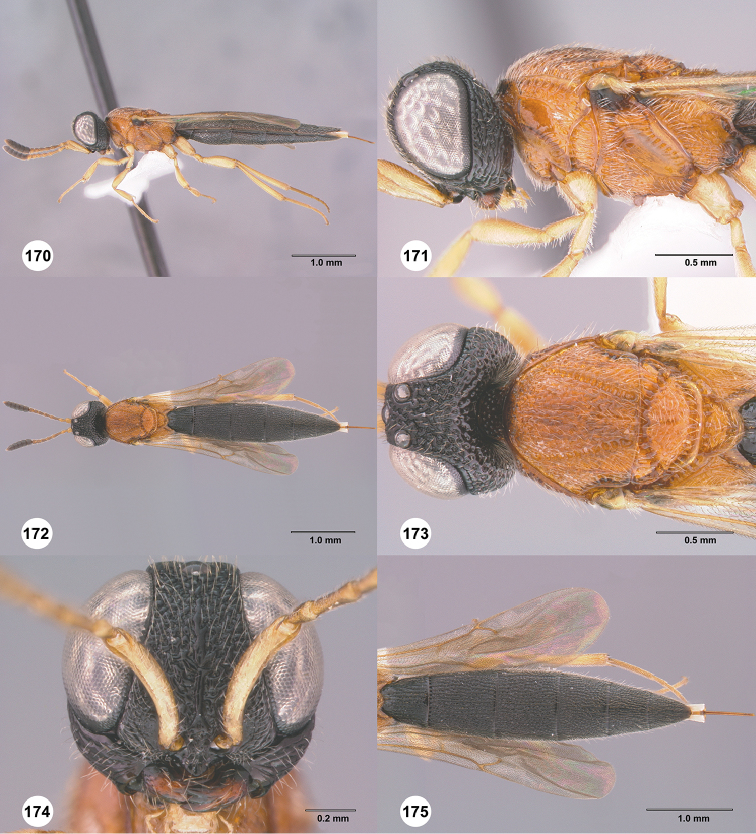
*Chromoteleiapilus* sp. n., female, holotype (OSUC584261). **170** Lateral habitus **171** Head and mesosoma, lateral view **172** Dorsal habitus **173** Head and mesosoma, dorsal view **174** Head, anterior view **175** Metasoma, dorsal view.

Figures 24 , 170–175

#### Description.

Body length of female: 4.98–5.90 mm (n = 20). Body length of male: 4.73–5.33 mm (n = 20). Color of A1: yellow to orange. A6 in female: distinctly wider than long. A5 in female: distinctly longer than wide. A6 in male: as long as wide. Number of basiconic sensilla on A7: 0. Number of basiconic sensilla on A12: 2. Sculpture of dorsal A1: striate. Color of head: black. Sculpture of frons directly above interantennal process: punctate rugose. Central keel: present, interrupted medially. Ventral margin of clypeus: pointed. Granulate microsculpture of dorsal frons: present. Occipital carina: interrupted medially. Granulate microsculpture of vertex: present. Sculpture of occiput: rugose. Sculpture of gena: narrowly smooth ventrally, punctate rugose dorsally.

Color of mesosoma: orange. Sculpture of epicoxal lobe posterior of propleural epicoxal sulcus: densely punctate. Sculpture of lateral pronotal area above pronotal cervical sulcus: smooth throughout. Sculpture of netrion: transversely striate. Microsculpture of mesoscutum: granulate. Macrosculpture of mesoscutal midlobe: punctate rugose throughout. Macrosculpture of lateral lobe of mesoscutum: punctate rugose. Sculpture of notaulus: foveate. Notaular foveae: discrete. Median mesoscutal carina: present along full length of mesoscutum; present anteriorly, not extending to posterior margin of mesoscutum. Mesoscutellum in lateral view: convex. Sculpture of mesoscutellum: smooth medially, densely punctate laterally. Shape of metascutellum: trapezoidal with broad posterior margin. Median metascutellar carina: absent or indistinguishable from sculpture. Sculpture of metascutellum: rugose. Sculpture of lateral propodeal area: rugose. Mesopleural carina: present. Sculpture of mesepisternum below femoral depression: punctate throughout. Sculpture of dorsal metapleural area: rugose. Setation of dorsal metapleural area: present. Setation of area directly dorsal to the metapleural triangle: present. Sculpture of ventral metapleural area: rugose throughout. Color of legs: orange yellow throughout. Length of hind basitarsus: about as long as remaining segments combined. Sculpture of hind coxa: densely punctate.

Length of postmarginal vein: distinctly longer than stigmal vein.

Color of metasoma in female: black. Horn on T1 in female: present. Striae of posterior margin of T1 in female: dense. Striae of T1 in male: dense. Transverse sulcus on T2: present. Sculpture of T2: densely longitudinally striate, punctate rugulose in interstices. Sculpture of T6 in female: densely longitudinally striate, with fine punctures in interstices. Length of T6 in female: approximately as long as wide. Shape of T6 in female in lateral view: flat. Apical spine on female T6: absent. Sculpture of T6 in male: densely punctate. Sculpture of T7 in male: coriaceous anteriorly, densely punctate posteriorly. Posterior margin of T7 in male: straight. Sculpture of medial S2: densely longitudinally striate with fine punctures in interstices.

#### Diagnosis.

This species is similar to *C.depilis* in color and size, but it can be distinguished by the combination of the following characters: dorsal metapleural area with setation, A6 in female as wide as long.

#### Etymology.

The epithet is inspired by the Latin word for hair, in reference to the setose dorsal metapleuron, and is intended to be treated as a noun.

#### Link to distribution map.

[http://hol.osu.edu/map-large.html?id=452225]

#### Material examined.

Holotype, female: **COSTA RICA**: Puntarenas Prov., road to Rincón, 24 km W Pan-American Highway, 200 m, II-1989 – III-1989, Hanson & Gauld, OSUC584261 (deposited in CNCI). *Paratypes*: (53 females, 31 males) **COSTA RICA**: 49 females, 31 males, OSUC149651, 202550, 202553, 202555–202556, 203109 (AEIC); OSUC556972, 556986, 556988, 556992, 557002, 557011–557012, 557049, 557061, 557083, 577903, 577907, 577933, 577935–577937, 577941, 577953, 577988, 578002–578004, 578058, 578082–578083, 578103, 583440, 583448, 583717, 583723, 583817, 583880, 583887, 583943–583944, 584032, 584035, 584037–584039, 584236, 584238, 584262, 586170, 586173, 586210, 586290, 586310, 586512, 586533, 586544, 586644, 586680, 586747, 586772, 586836 (CNCI); OSUC202551, 202554, 556989, 557040, 56234, 578005, 578081, 578091, 583882, 583886, 583940, 584041, 584247, 586509, 586545, 586646, 586780 (OSUC); OSUC221941 (TAMU). **MEXICO**: 1 female, OSUC584145 (CNCI). **PANAMA**: 3 females, OSUC584378, 584381, 586504 (CNCI).

### Chromoteleia plana

Taxon classificationAnimaliaHymenopteraScelionidae

Chen & Johnson sp. n.

http://zoobank.org/DA401CB2-DCD9-405A-83AE-8159593A1510

http://bioguid.osu.edu/xbiod_concepts/452218

Figures 176–181

#### Description.

Body length of female: 7.31 mm (n = 1). Color of A1: yellow to orange. A6 in female: distinctly wider than long. A5 in female: as long as wide. Number of basiconic sensilla on A7: 1. Number of basiconic sensilla on A12: 1. Sculpture of dorsal A1: punctate; smooth. Color of head: black. Sculpture of frons directly above interantennal process: areolate. Central keel: present only in ventral portion of frons. Ventral margin of clypeus: straight. Granulate microsculpture of dorsal frons: absent. Occipital carina: complete. Granulate microsculpture of vertex: absent. Sculpture of occiput: smooth. Sculpture of gena: narrowly smooth ventrally, punctate rugose dorsally.

Color of mesosoma: variably orange to black. Sculpture of epicoxal lobe posterior of propleural epicoxal sulcus: sparsely punctate. Sculpture of lateral pronotal area above pronotal cervical sulcus: smooth dorsally, rugose ventrally. Sculpture of netrion: transversely striate. Microsculpture of mesoscutum: absent. Macrosculpture of mesoscutal midlobe: sparsely punctate at anterior margin, smooth posteriorly. Macrosculpture of lateral lobe of mesoscutum: smooth. Sculpture of notaulus: foveate. Notaular foveae: interconnected. Median mesoscutal carina: present anteriorly, not extending to posterior margin of mesoscutum. Shape of mesoscutellum: strongly transverse. Mesoscutellum in lateral view: flat. Sculpture of mesoscutellum: smooth throughout. Shape of metascutellum: trapezoidal with broad posterior margin. Median metascutellar carina: absent or indistinguishable from sculpture. Sculpture of metascutellum: areolate. Sculpture of lateral propodeal area: rugose. Mesopleural carina: present. Sculpture of mesepisternum below femoral depression: largely smooth, punctate rugose anteriorly and directly below femoral depression. Sculpture of dorsal metapleural area: rugose. Setation of dorsal metapleural area: absent. Setation of area directly dorsal to the metapleural triangle: absent. Sculpture of ventral metapleural area: rugose throughout. Color of legs: orange yellow with coxae dark brown. Length of hind basitarsus: distinctly longer than remaining segments combined. Sculpture of hind coxa: densely punctate.

Length of postmarginal vein: approximately as long as stigmal vein.

Color of metasoma in female: black. Horn on T1 in female: present. Striae of posterior margin of T1 in female: dense. Transverse sulcus on T2: present. Sculpture of T2: densely longitudinally striate, punctate rugulose in interstices, with a narrow smooth strip medially. Sculpture of T6 in female: densely longitudinally striate, with fine punctures in interstices. Length of T6 in female: at least 1.5× longer than wide. Shape of T6 in female in lateral view: flat. Apical spine on female T6: absent. Sculpture of medial S2: punctate rugose, with a narrow smooth strip medially.

**Figures 176–181.**
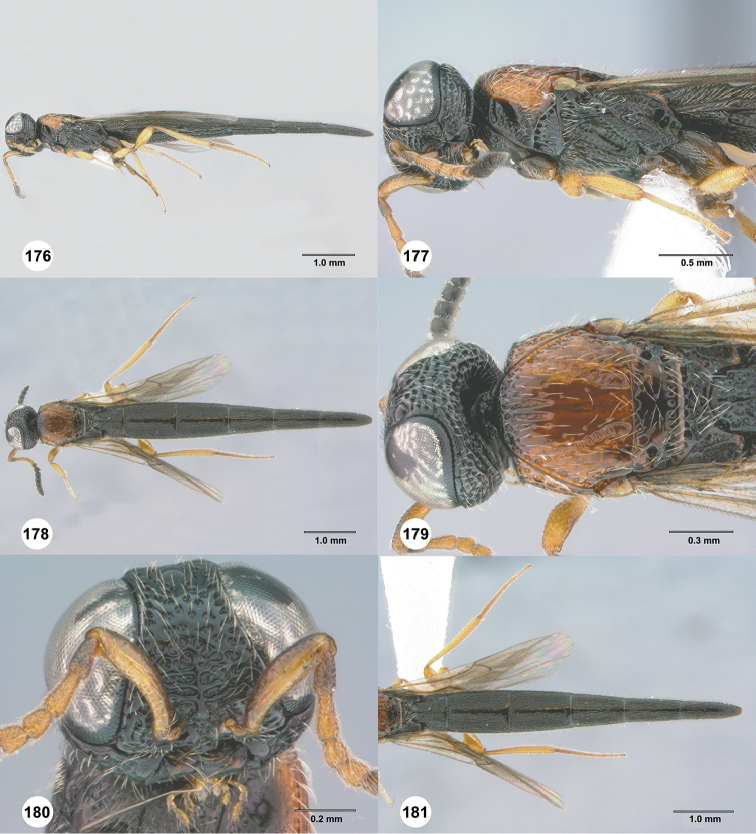
*Chromoteleiaplana* sp. n., female, holotype (OSUC577460). **176** Lateral habitus **177** Head and mesosoma, lateral view **178** Dorsal habitus **179** Head and mesosoma, dorsal view **180** Head, anterior view **181** Metasoma, dorsal view.

#### Diagnosis.

This species can be easily distinguished by the following characters: body extremely elongate, central keel present only in ventral portion of frons, mesoscutellum strongly transverse, and T2–T6 with with a narrow smooth strip medially.

#### Etymology.

The epithet is inspired by the Latin word for flat or plain, in reference to the flat and smooth mesoscutellum, and is intended to be treated as an adjective.

#### Link to distribution map.

[http://hol.osu.edu/map-large.html?id=452218]

#### Material examined.

Holotype, female: **FRENCH GUIANA**: Cayenne Arrond., 323 m, 04°33.998'N, 52°12.442'W, Kaw Mountains, 15.XII–18.XII.2011, Malaise trap, A. Desjardins, OSUC577460 (deposited in CNCI).

### Chromoteleia rara

Taxon classificationAnimaliaHymenopteraScelionidae

Chen & Johnson sp. n.

http://zoobank.org/0E7CB667-807C-4C4B-9858-BFE942BBAD19

http://bioguid.osu.edu/xbiod_concepts/452222

Figures 7–8 , 182–187

#### Description.

Body length of female: 4.68–5.30 mm (n = 20). Body length of male: 4.50–5.13 mm (n = 20). Color of A1: yellow to orange. A6 in female: as wide as long. A5 in female: distinctly longer than wide. A6 in male: as long as wide. Number of basiconic sensilla on A7: 1; 2. Number of basiconic sensilla on A12: 1. Sculpture of dorsal A1: smooth. Color of head: black. Sculpture of frons directly above interantennal process: areolate. Central keel: present only in ventral portion of frons. Ventral margin of clypeus: pointed. Granulate microsculpture of dorsal frons: absent. Occipital carina: complete. Granulate microsculpture of vertex: absent. Sculpture of occiput: smooth. Sculpture of gena: dorsoventrally strigose.

Color of mesosoma: variably orange to black. Sculpture of epicoxal lobe posterior of propleural epicoxal sulcus: smooth. Sculpture of lateral pronotal area above pronotal cervical sulcus: smooth dorsally, rugose ventrally. Sculpture of netrion: transversely striate. Microsculpture of mesoscutum: granulate. Macrosculpture of mesoscutal midlobe: punctate rugose anteriorly, sparsely punctate posteriorly. Macrosculpture of lateral lobe of mesoscutum: densely punctate. Sculpture of notaulus: foveate. Notaular foveae: interconnected. Median mesoscutal carina: present anteriorly, not extending to posterior margin of mesoscutum. Mesoscutellum in lateral view: convex. Sculpture of mesoscutellum: smooth medially, densely punctate laterally. Shape of metascutellum: trapezoidal with broad posterior margin. Median metascutellar carina: present. Sculpture of metascutellum: rugose. Sculpture of lateral propodeal area: rugose. Sculpture of mesepisternum below femoral depression: punctate rugose. Sculpture of dorsal metapleural area: rugose. Setation of dorsal metapleural area: absent. Setation of area directly dorsal to the metapleural triangle: present. Sculpture of ventral metapleural area: rugose throughout. Color of legs: orange yellow throughout. Length of hind basitarsus: distinctly longer than remaining segments combined. Sculpture of hind coxa: densely punctate.

Length of postmarginal vein: distinctly shorter than stigmal vein.

Color of metasoma in female: black. Color of metasoma in male: black. Horn on T1 in female: present. Striae of posterior margin of T1 in female: dense. Striae of T1 in male: dense. Transverse sulcus on T2: present. Sculpture of T2: densely longitudinally striate, punctate rugulose in interstices. Sculpture of T6 in female: densely longitudinally striate, with fine punctures in interstices. Length of T6 in female: at least 1.5× longer than wide. Shape of T6 in female in lateral view: flat. Apical spine on female T6: absent. Sculpture of T7 in male: coriaceous anteriorly, densely punctate posteriorly. Posterior margin of T7 in male: straight. Sculpture of medial S2: densely longitudinally striate with fine punctures in interstices.

**Figures 182–187.**
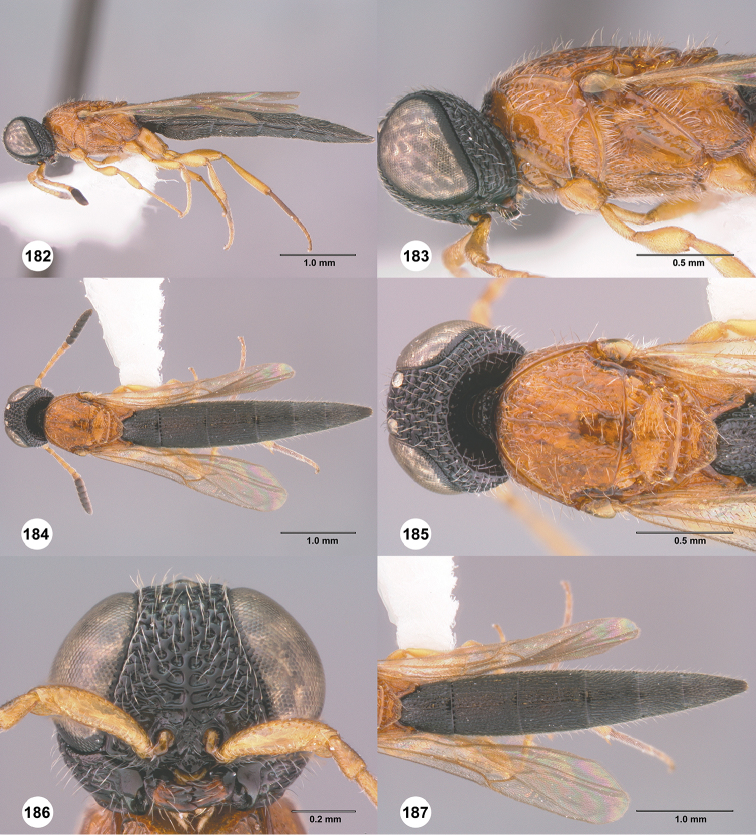
*Chromoteleiarara* sp. n., female, holotype (OSUC577495). **182** Lateral habitus **183** Head and mesosoma, lateral view **184** Dorsal habitus **185** Head and mesosoma, dorsal view **186** Head, anterior view **187** Metasoma, dorsal view. (NOTE: small form)

**Figures 188–193.**
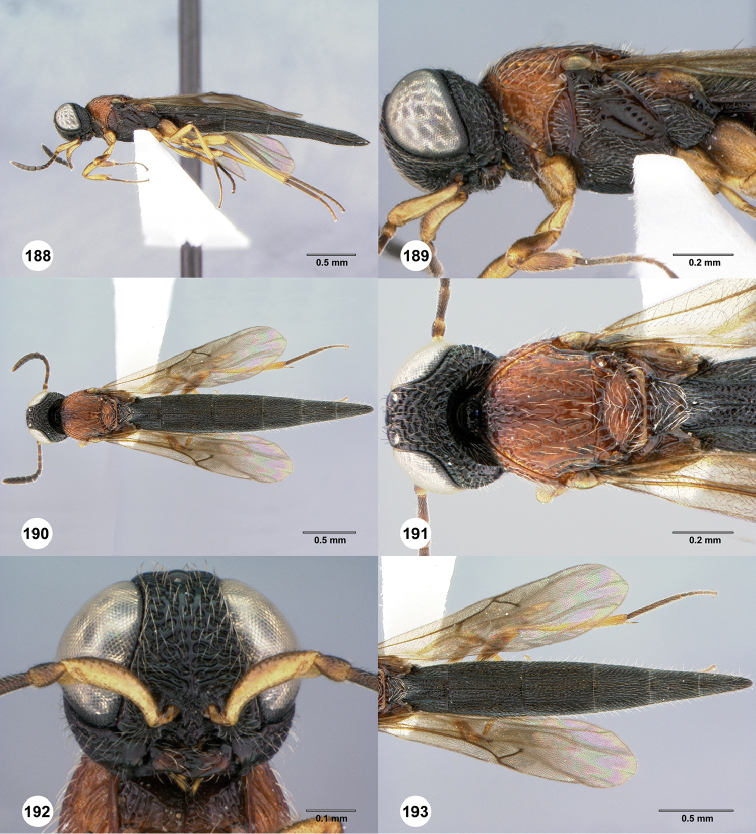
*Chromoteleiarara* sp. n., female, paratype (OSUC557090). **188** Lateral habitus **189** Head and mesosoma, lateral view **190** Dorsal habitus **191** Head and mesosoma, dorsal view **192** Head, anterior view **193** Metasoma, dorsal view. (NOTE: large form)

#### Diagnosis.

This species is similar to *C.cuneus*, with which it shares a short postmarginal vein, and it can be distinguished by the rounded apex of T6 in dorsal view.

#### Etymology.

The epithet is inspired by the Latin word for uncommon. We apply it to this species because this species is the only species in *Chromoteleia* that can have either one or two basiconic sensilla on A7. The name is treated as an adjective in apposition.

#### Link to distribution map.

[http://hol.osu.edu/map-large.html?id=452222]

#### Material examined.

Holotype, female: **COSTA RICA**: Heredia Prov., Puerto Viejo, La Selva Biological Station, 100 m, II.1993 – III.1993, Malaise trap, P. Hanson, OSUC577495 (deposited in CNCI). *Paratypes*: (45 females, 28 males) **BELIZE**: 2 females, 1 male, OSUC577509–577510 (CNCI); OSUC 93700 (OSUC). BRAZIL: 1 male, OSUC586574 (CNCI). **COLOMBIA**: 3 females, OSUC199606, 199612 (FSCA); OSUC268912 (OSUC). **COSTA RICA**: 27 females, 17 males, OSUC202552 (AEIC); OSUC253964, 556978–556979, 556981, 557005–557006, 577489, 577491, 577493, 577904, 577906, 577945–577946, 577973, 577979, 577991, 578079, 578086, 583758, 583879, 583898, 583925, 583949, 584025, 584235, 586267, 586378, 586480, 586618, 586662, 586664 (CNCI); OSUC556997, 577490, 577492, 577494, 577912, 577934, 577939, 577948, 584010, 586782, 586784 (OSUC); OSUC204983 (UCDC). **ECUADOR**: 3 females, 8 males, OSUC202545–202548 (AEIC); OSUC577432, 577434–577435, 584106, 584756 (CNCI); OSUC199608, 199610 (FSCA). **FRENCH GUIANA**: 1 female, OSUC 47022 (OSUC). **HONDURAS**: 2 females, OSUC369621, 413783 (MZLU). **PANAMA**: 6 females, 1 male, OSUC586819 (CNCI); OSUC320639 (OSUC); OSUC221918–221920, 320644 (TAMU); OSUC205000 (UCDC). **TRINIDAD AND TOBAGO**: 1 female, OSUC586288 (CNCI).

#### Comments.

This species is extremely variable in body length, which may be an indication that it attacks multiple species with different size eggs, or that the eggs of its host(s) vary widely in size.

### Chromoteleia robusta

Taxon classificationAnimaliaHymenopteraScelionidae

Chen & Johnson sp. n.

http://zoobank.org/A38A572D-E20C-418F-8988-235126D46E8E

http://bioguid.osu.edu/xbiod_concepts/452215

**Figures 194–199.**
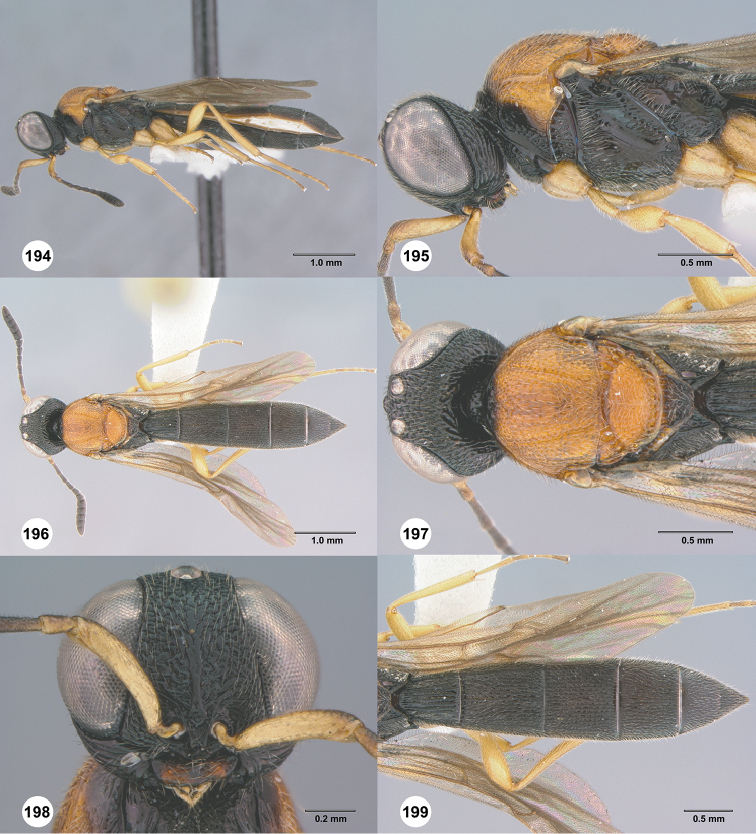
*Chromoteleiarobusta* sp. n., female, holotype (OSUC577466). **194** Lateral habitus **195** Head and mesosoma, lateral view **196** Dorsal habitus **197** Head and mesosoma, dorsal view **198** Head, anterior view **199** Metasoma, dorsal view.

Figures 12 , 194–199

#### Description.

Body length of female: 5.18–5.53 mm (n = 7). Body length of male: 4.30–4.93 mm (n = 2). Color of A1: yellow to orange. A6 in female: distinctly longer than wide. A5 in female: distinctly longer than wide. A6 in male: as long as wide. Number of basiconic sensilla on A7: 0. Number of basiconic sensilla on A12: 2. Sculpture of dorsal A1: striate. Color of head: black. Sculpture of frons directly above interantennal process: punctate rugose. Central keel: complete, extending from interantennal process to median ocellus. Ventral margin of clypeus: straight. Granulate microsculpture of dorsal frons: absent. Occipital carina: interrupted medially. Granulate microsculpture of vertex: absent. Sculpture of occiput: smooth medially, striate laterally. Sculpture of gena: dorsoventrally strigose.

Color of mesosoma: variably orange to black. Sculpture of epicoxal lobe posterior of propleural epicoxal sulcus: smooth. Sculpture of lateral pronotal area above pronotal cervical sulcus: smooth throughout. Sculpture of netrion: transversely striate. Microsculpture of mesoscutum: granulate. Macrosculpture of mesoscutal midlobe: punctate rugose throughout. Macrosculpture of lateral lobe of mesoscutum: punctate rugose. Sculpture of notaulus: foveate. Notaular foveae: interconnected. Median mesoscutal carina: absent. Mesoscutellum in lateral view: convex. Sculpture of mesoscutellum: densely punctate rugose. Shape of metascutellum: trapezoidal with broad posterior margin. Median metascutellar carina: absent or indistinguishable from sculpture. Sculpture of metascutellum: rugose. Mesopleural carina: present. Sculpture of mesepisternum below femoral depression: punctate rugose. Sculpture of dorsal metapleural area: rugose; smooth. Setation of dorsal metapleural area: absent. Setation of area directly dorsal to the metapleural triangle: absent. Sculpture of ventral metapleural area: rugose anteriorly, smooth posteriorly. Color of legs: orange yellow throughout. Length of hind basitarsus: about as long as remaining segments combined. Sculpture of hind coxa: densely punctate.

Length of postmarginal vein: approximately as long as stigmal vein.

Color of metasoma in female: black. Color of metasoma in male: black. Horn on T1 in female: absent. Striae of posterior margin of T1 in female: dense. Striae of T1 in male: dense. Transverse sulcus on T2: present. Sculpture of T2: densely longitudinally striate, punctate rugulose in interstices. Sculpture of T6 in female: longitudinally punctate rugose. Length of T6 in female: approximately as long as wide; wider than long. Shape of T6 in female in lateral view: sinuate. Apical spine on female T6: present. Sculpture of T6 in male: densely punctate. Sculpture of T7 in male: granulate. Posterior margin of T7 in male: straight. Sculpture of medial S2: densely longitudinally striate with fine punctures in interstices.

Length of postmarginal vein: approximately as long as stigmal vein.

#### Diagnosis.

This species is similar to *C.feng* in the acute apex of T6, and it can be distinguished by the combination of the following characters: female A12 with two basiconic sensilla, occipital carina interrupted medially, notaulus foveate.

#### Etymology.

The epithet is inspired by the Latin word for strong or robust, in reference to the robust appearance of this species, and is intended to be treated as an adjective.

#### Link to distribution map.

[http://hol.osu.edu/map-large.html?id=452215]

#### Material examined.

Holotype, female: **VENEZUELA**: Aragua St., La Cumbre trail, Rancho Grande, IV.1987 – V.1987, flight intercept trap, OSUC577466 (deposited in CNCI). *Paratypes*: **VENEZUELA**: 6 females, 2 males, OSUC210349 (AMNH); OSUC577462–577465, 577467–577469 (CNCI).

### Chromoteleia rufithorax

Taxon classificationAnimaliaHymenopteraScelionidae

Kieffer

http://zoobank.org/ECD55F87-65C5-4C1E-93DC-BB6974CA164A

http://bioguid.osu.edu/xbiod_concepts/4215

Chromoteleia rufithorax Kieffer, 1907: 266 (original description, keyed); Kieffer, 1908a: 25 (described as new); Kieffer, 1910a: 313 (keyed); Johnson, 1992: 364 (cataloged).
Petalosema rufithorax (Kieffer): Kieffer, 1926: 358 (generic transfer, description, keyed).

#### Comments.

We were not able to locate the type specimens of this species, and its status and identity are unclear.

### Chromoteleia semicyanea

Taxon classificationAnimaliaHymenopteraScelionidae

Ashmead

http://zoobank.org/5D560202-AF14-47AB-AB0A-70E96B8665C7

http://bioguid.osu.edu/xbiod_concepts/4216

Figures 200–205

Chromoteleia semicyanea Ashmead, 1893: 220 (original description); Ashmead, 1894: 224 (redescribed as new); Ashmead, 1900: 327 (distribution); Kieffer, 1907: 266 (keyed); Kieffer, 1910a: 312 (keyed); Kieffer, 1926: 406 (description); Masner, 1976: 25 (type information, lectotype designation); Johnson, 1992: 364 (cataloged, type information).

#### Description.

Body length of female: 5.48–5.60 mm (n = 2). Body length of male: 4.80–4.93 mm (n = 3). Color of A1: yellow to orange. A6 in female: as wide as long. A5 in female: distinctly longer than wide. A6 in male: approximately 2.0× longer than wide. Number of basiconic sensilla on A7: 1. Number of basiconic sensilla on A12: 1. Sculpture of dorsal A1: punctate; smooth. Color of head: blue. Sculpture of frons directly above interantennal process: transversely striate to rugose. Central keel: absent. Ventral margin of clypeus: pointed. Granulate microsculpture of dorsal frons: absent. Occipital carina: interrupted medially. Granulate microsculpture of vertex: absent. Sculpture of occiput: smooth. Sculpture of gena: dorsoventrally strigose.

Color of mesosoma: blue dorsally, black laterally. Sculpture of epicoxal lobe posterior of propleural epicoxal sulcus: densely punctate. Sculpture of lateral pronotal area above pronotal cervical sulcus: smooth throughout. Sculpture of netrion: rugose. Microsculpture of mesoscutum: coriaceous. Macrosculpture of mesoscutal midlobe: sparsely punctate. Macrosculpture of lateral lobe of mesoscutum: sparsely punctate. Sculpture of notaulus: foveate. Notaular foveae: interconnected. Median mesoscutal carina: present anteriorly, not extending to posterior margin of mesoscutum. Mesoscutellum in lateral view: convex. Sculpture of mesoscutellum: smooth medially, densely punctate laterally. Shape of metascutellum: trapezoidal with broad posterior margin. Median metascutellar carina: absent or indistinguishable from sculpture. Sculpture of metascutellum: rugose. Sculpture of lateral propodeal area: rugose. Mesopleural carina: absent. Sculpture of mesepisternum below femoral depression: punctate throughout. Sculpture of dorsal metapleural area: rugose. Setation of dorsal metapleural area: absent. Setation of area directly dorsal to the metapleural triangle: present. Sculpture of ventral metapleural area: rugose throughout. Color of legs: orange yellow throughout. Length of hind basitarsus: distinctly longer than remaining segments combined. Sculpture of hind coxa: largely smooth, with sparse fine punctures.

Length of postmarginal vein: distinctly longer than stigmal vein.

Color of metasoma in female: orange. Color of metasoma in male: orange. Horn on T1 in female: present. Striae of posterior margin of T1 in female: dense. Striae of T1 in male: dense. Transverse sulcus on T2: present. Sculpture of T2: densely longitudinally striate, punctate rugulose in interstices. Sculpture of T6 in female: densely longitudinally striate, with fine punctures in interstices. Length of T6 in female: at least 1.5× longer than wide. Shape of T6 in female in lateral view: flat. Apical spine on female T6: absent. Sculpture of T6 in male: densely punctate. Sculpture of T7 in male: smooth anteriorly, rugulose posteriorly. Posterior margin of T7 in male: straight. Sculpture of medial S2: densely punctate to punctate rugose.

**Figures 200–205.**
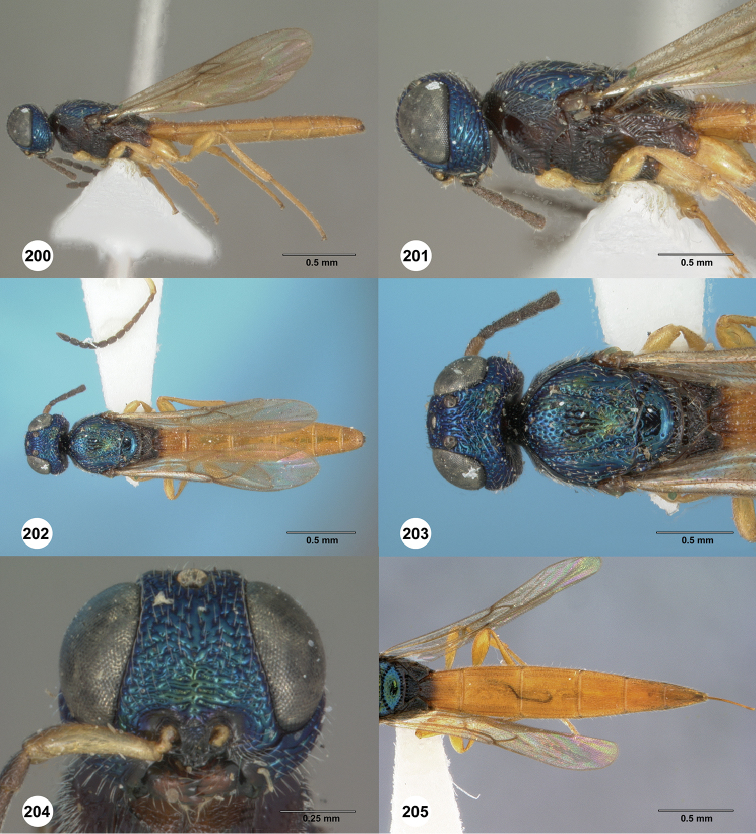
*Chromoteleiasemicyanea* Ashmead, male, holotype (USNM Type No. 2251). **200** Lateral habitus **201** Head and mesosoma, lateral view **202** Dorsal habitus **203** Head and mesosoma, dorsal view **204** Head, anterior view **205** female (OSUC584765) Metasoma, dorsal view.

#### Diagnosis.

This beautiful species can be distinguished from other *Chromoteleia* species by the following combination of characters: head and dorsal mesosoma blue, metasoma orange, frons without central keel, mesopleural carina absent.

#### Link to distribution map.

[http://hol.osu.edu/map-large.html?id=4216]

#### Material examined.

Lectotype, male: **SAINT VINCENT AND THE GRENADINES**: Saint Vincent Island, 2000ft, no date, H. H. Smith, USNM Type No. 2251 (deposited in USNM). Other material: **SAINT VINCENT AND THE GRENADINES**: 2 females, 2 males, OSUC584763–584766 (CNCI).

### Chromoteleia semilutea

Taxon classificationAnimaliaHymenopteraScelionidae

Chen & Johnson sp. n.

http://zoobank.org/7F8871F3-3D8E-4985-9CC5-850F70A1558F

http://bioguid.osu.edu/xbiod_concepts/452219

Figures 206–211

#### Description.

Body length of female: 5.28–5.50 mm (n = 10). Body length of male: 4.30–4.83 mm (n = 3). Color of A1: yellow to orange. A6 in female: as wide as long. A5 in female: distinctly longer than wide. A6 in male: as long as wide. Number of basiconic sensilla on A7: 0. Number of basiconic sensilla on A12: 2. Sculpture of dorsal A1: striate. Color of head: black. Sculpture of frons directly above interantennal process: transversely striate to rugose. Central keel: complete, extending from interantennal process to median ocellus. Ventral margin of clypeus: straight. Granulate microsculpture of dorsal frons: absent. Occipital carina: interrupted medially. Granulate microsculpture of vertex: absent. Sculpture of occiput: smooth. Sculpture of gena: dorsoventrally strigose.

Color of mesosoma: orange. Sculpture of epicoxal lobe posterior of propleural epicoxal sulcus: smooth. Sculpture of lateral pronotal area above pronotal cervical sulcus: smooth throughout. Sculpture of netrion: transversely striate. Microsculpture of mesoscutum: granulate. Macrosculpture of mesoscutal midlobe: punctate rugose throughout. Macrosculpture of lateral lobe of mesoscutum: punctate rugose. Sculpture of notaulus: foveate. Notaular foveae: interconnected. Median mesoscutal carina: present along full length of mesoscutum. Mesoscutellum in lateral view: convex. Sculpture of mesoscutellum: densely punctate rugose. Shape of metascutellum: trapezoidal with broad posterior margin. Median metascutellar carina: present. Sculpture of metascutellum: rugose. Sculpture of lateral propodeal area: smooth. Mesopleural carina: absent. Sculpture of mesepisternum below femoral depression: largely smooth, punctate rugose anteriorly and directly below femoral depression. Sculpture of dorsal metapleural area: rugose. Setation of dorsal metapleural area: absent. Setation of area directly dorsal to the metapleural triangle: absent. Sculpture of ventral metapleural area: rugose throughout. Color of legs: orange yellow throughout. Length of hind basitarsus: about as long as remaining segments combined. Sculpture of hind coxa: largely smooth, with sparse fine punctures.

Length of postmarginal vein: distinctly longer than stigmal vein.

Color of metasoma in female: mostly black with T1–T3 orange to dark brown. Color of metasoma in male: mostly black with T1–T3 orange to dark brow. Horn on T1 in female: present. Striae of posterior margin of T1 in female: dense. Striae of T1 in male: dense. Transverse sulcus on T2: present. Sculpture of T2: densely longitudinally striate, punctate rugulose in interstices. Sculpture of T6 in female: longitudinally punctate rugose. Length of T6 in female: approximately as long as wide. Shape of T6 in female in lateral view: flat. Apical spine on female T6: absent. Sculpture of T6 in male: densely punctate. Sculpture of T7 in male: granulate. Posterior margin of T7 in male: straight. Sculpture of medial S2: densely punctate to punctate rugose.

**Figures 206–211.**
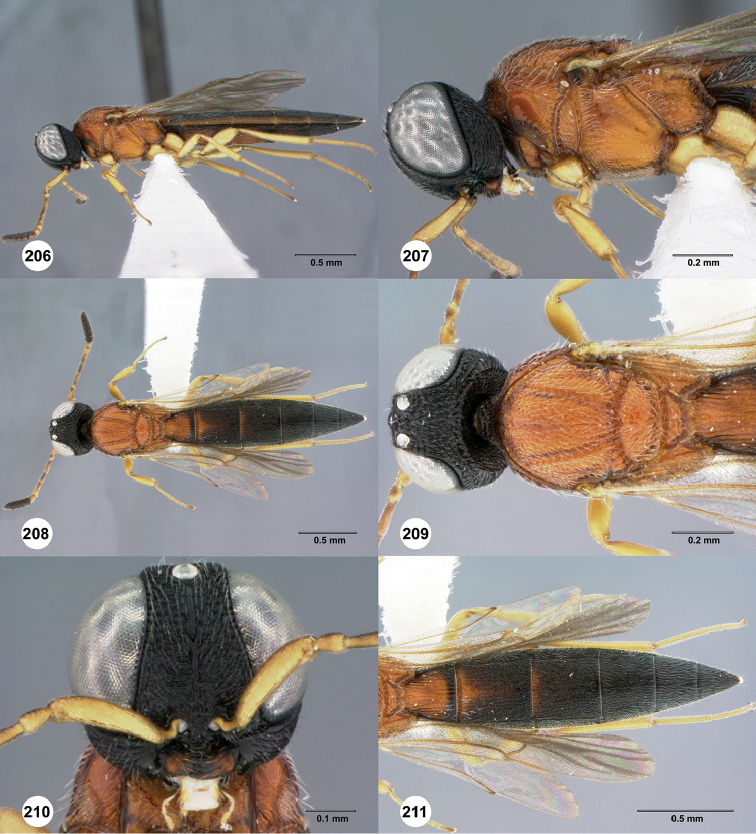
*Chromoteleiasemilutea* sp. n., female, holotype (OSUC577506). **206** Lateral habitus **207** Head and mesosoma, lateral view **208** Dorsal habitus **209** Head and mesosoma, dorsal view **210** Head, anterior view **211** Metasoma, dorsal view.

#### Diagnosis.

This species can be distinguished from other *Chromoteleia* species by the following combination of characters: metasoma with T1–T3 orange to dark brown, frons with central keel complete, mesopleural carina absent.

#### Etymology.

The name semilutea refers to the half orange metasoma of this species and is used as an adjective in apposition.

#### Link to distribution map.

[http://hol.osu.edu/map-large.html?id=452219]

#### Material examined.

Holotype, female: **BELIZE**: Orange Walk Dist., Rio Bravo Conservation and Management Area, 15.VII–21.VII.1996, Malaise trap, P. Kovarik, OSUC577506 (deposited in CNCI). *Paratypes*: (9 females, 3 males) **BELIZE**: 3 females, 1 male, OSUC577505 (CNCI); OSUC225288, 232143, 47912 (OSUC). **MEXICO**: 6 females, 2 males, OSUC556945–556946, 577501–577504, 586774 (CNCI); OSUC586773 (OSUC).

### Chromoteleia sparsa

Taxon classificationAnimaliaHymenopteraScelionidae

Chen & Johnson sp. n.

http://zoobank.org/C074AD5B-E19B-4F8F-B403-BE9B5A81AEA5

http://bioguid.osu.edu/xbiod_concepts/452213

Figures 212–217

#### Description.

Body length of female: 5.38–5.70 mm (n = 20). Body length of male: 4.90–5.15mm (n = 20). Color of A1: yellow to orange. A6 in female: as wide as long. A5 in female: distinctly longer than wide. A6 in male: approximately 2.0× longer than wide. Number of basiconic sensilla on A7: 1. Number of basiconic sensilla on A12: 1. Sculpture of dorsal A1: punctate; smooth. Color of head: black. Sculpture of frons directly above interantennal process: areolate. Central keel: present, interrupted medially. Ventral margin of clypeus: pointed. Granulate microsculpture of dorsal frons: absent. Occipital carina: complete. Granulate microsculpture of vertex: absent. Sculpture of occiput: smooth medially, striate laterally. Sculpture of gena: punctate rugose dorsally and ventrally, strigose medially.

Color of mesosoma: variably orange to nearly black. Sculpture of epicoxal lobe posterior of propleural epicoxal sulcus: densely punctate. Sculpture of lateral pronotal area above pronotal cervical sulcus: smooth dorsally, rugose ventrally. Sculpture of netrion: rugose. Microsculpture of mesoscutum: absent. Macrosculpture of mesoscutal midlobe: punctate rugose anteriorly, sparsely punctate posteriorly. Macrosculpture of lateral lobe of mesoscutum: sparsely punctate. Sculpture of notaulus: foveate. Notaular foveae: interconnected. Median mesoscutal carina: present anteriorly, not extending to posterior margin of mesoscutum. Mesoscutellum in lateral view: flat. Sculpture of mesoscutellum: smooth medially, densely punctate laterally. Shape of metascutellum: trapezoidal with broad posterior margin. Median metascutellar carina: absent or indistinguishable from sculpture. Sculpture of metascutellum: areolate. Sculpture of lateral propodeal area: rugose. Mesopleural carina: present. Sculpture of mesepisternum below femoral depression: punctate throughout. Sculpture of dorsal metapleural area: rugose. Setation of dorsal metapleural area: absent. Setation of area directly dorsal to the metapleural triangle: present. Sculpture of ventral metapleural area: rugose throughout. Color of legs: orange yellow throughout. Length of hind basitarsus: about as long as remaining segments combined. Sculpture of hind coxa: largely smooth, with sparse fine punctures.

Length of postmarginal vein: distinctly shorter than stigmal vein.

Color of metasoma in female: black. Color of metasoma in male: black. Horn on T1 in female: present. Striae of posterior margin of T1 in female: dense. Striae of T1 in male: sparse. Transverse sulcus on T2: present. Sculpture of T2: densely longitudinally striate, punctate rugulose in interstices. Sculpture of T6 in female: densely longitudinally striate, with fine punctures in interstices. Length of T6 in female: at least 1.5× longer than wide. Shape of T6 in female in lateral view: flat. Apical spine on female T6: absent. Sculpture of T6 in male: densely longitudinally striate with fine punctures in interstices. Sculpture of T7 in male: granulate. Posterior margin of T7 in male: emarginate medially between rounded projections. Sculpture of medial S2: densely longitudinally striate with fine punctures in interstices.

**Figures 212–217.**
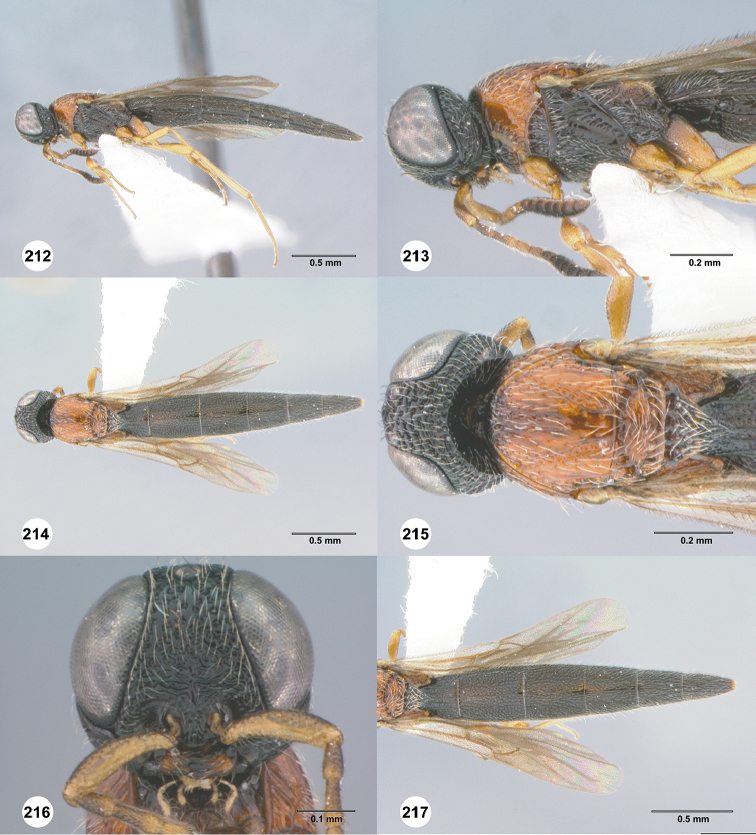
*Chromoteleiasparsa* sp. n., female, holotype (OSUC584751). **212** Lateral habitus **213** Head and mesosoma, lateral view **214** Dorsal habitus **215** Head and mesosoma, dorsal view **216** Head, anterior view **217** Metasoma, dorsal view. (NOTE: light form)

**Figures 218–223.**
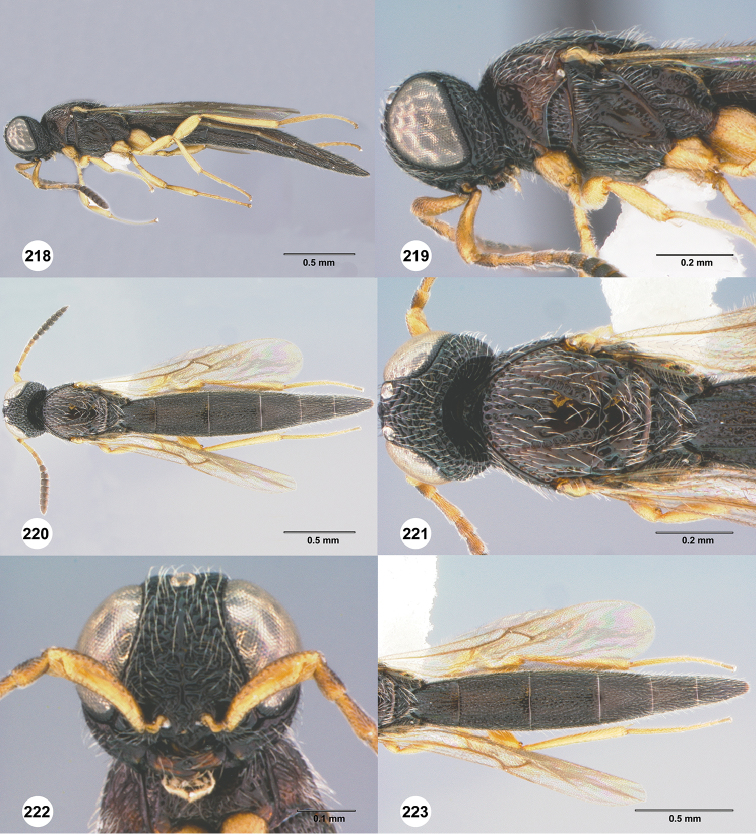
*Chromoteleiasparsa* sp. n., female, paratype (OSUC583488). **218** Lateral habitus **219** Head and mesosoma, lateral view **220** Dorsal habitus **221** Head and mesosoma, dorsal view **222** Head, anterior view **223** Metasoma, dorsal view. (NOTE: dark form)

#### Diagnosis.

This species is similar to *C.copiosa*, but it can be distinguished by the combinations of the following characters: occipital carina complete, netrion rugose, and the hind basitarsus approximately as long as the remaining segments combined.

#### Etymology.

The epithet is inspired by the Latin word for scattered, in reference to the sparsely punctate posterior part of mesoscutum, and is intended to be treated as an adjective.

#### Link to distribution map.

[http://hol.osu.edu/map-large.html?id=452213]

#### Material examined.

Holotype, female: **ECUADOR**: Sucumbíos Prov., 270 m, 00°30'S, 76°30'W, Sacha Lodge, 13.VI–23.VI.1994, Malaise trap, P. Hibbs, OSUC584751 (deposited in CNCI). *Paratypes*: (64 females, 39 males) **ARGENTINA**: 1 female, OSUC584143 (CNCI). **BRAZIL**: 36 females, 30 males, OSUC149640–149641, 202482, 202504–202505, 202512, 202514, 202536 (AEIC); OSUC557091, 583487–583489, 583493, 583499, 583502, 583504, 583509, 584149, 586149, 586259, 586343–586344, 586347–586349, 586353–586355, 586361, 586577–586581, 586586–586587, 586596, 586598, 586600, 586603–586608 (CNCI); OSUC199635 (FSCA); OSUC225586, 241253 (MPEG); OSUC147541, 246558, 254870 (MZSP); OSUC138674, 147540, 225227, 235205, 246557, 254868–254869, 354648, 583486, 583500, 586350–586351, 586585, 586590 (OSUC); OSUC204990 (UCDC). **COLOMBIA**: 2 females, 2 males, OSUC584302 (CNCI); OSUC182714, 230409 (IAVH); OSUC230410 (OSUC). **COSTA RICA**: 4 females, 1 male, OSUC577923–577924, 586498, 586641 (CNCI); OSUC586175 (OSUC). **ECUADOR**: 9 females, 2 males, OSUC556944, 584748–584750, 584753–584755, 586339–586340 (CNCI); OSUC583707, 584752 (OSUC). **FRENCH GUIANA**: 7 females, OSUC555807, 555813, 586435, 586448, 586459 (CNCI); OSUC555811, 586863 (OSUC). **GUYANA**: 1 female, OSUC583434 (CNCI). **MEXICO**: 2 males, OSUC584295 (CNCI); OSUC221932 (TAMU). **TRINIDAD AND TOBAGO**: 1 male, OSUC586308 (CNCI). **VENEZUELA**: 4 females, 1 male, OSUC557088, 584141, 586362 (CNCI); OSUC251644, 251662 (MIZA).

#### Comments.

This species is extremely variable in mesosoma color and can be divided roughly into dark and light forms.

### Chromoteleia tricarinata

Taxon classificationAnimaliaHymenopteraScelionidae

Kieffer

http://zoobank.org/301D1A78-C6C1-4A91-8638-D3E7237C7611

http://bioguid.osu.edu/xbiod_concepts/4218

Figures 1–2 , 224–229 , 230–235 , 236

Chromoteleia rufithorax var. tricarinata Kieffer, 1909: 250 (original description).
Petalosema tricarinata (Kieffer): Kieffer, 1926: 358, 359 (generic transfer, description, change to species status, keyed).
Chromoteleia tricarinata Kieffer: Masner, 1976: 25 (type information, taxonomic status); Johnson, 1992: 364 (cataloged, type information).

#### Description.

Body length of female: 5.28–5.90 mm (n = 20). Body length of male: 5.20–5.83 mm (n = 20). Color of A1: yellow to orange. A6 in female: as wide as long. A5 in female: distinctly longer than wide. A6 in male: approximately 2.0× longer than wide. Number of basiconic sensilla on A7: 0. Number of basiconic sensilla on A12: 2. Sculpture of dorsal A1: striate. Color of head: black. Sculpture of frons directly above interantennal process: punctate rugose. Central keel: complete, extending from interantennal process to median ocellus. Ventral margin of clypeus: pointed. Granulate microsculpture of dorsal frons: present. Occipital carina: interrupted medially. Granulate microsculpture of vertex: present. Sculpture of occiput: smooth medially, striate laterally. Sculpture of gena: coarsely punctate rugose.

Color of mesosoma: orange. Sculpture of epicoxal lobe posterior of propleural epicoxal sulcus: smooth. Sculpture of lateral pronotal area above pronotal cervical sulcus: smooth throughout. Sculpture of netrion: transversely striate. Microsculpture of mesoscutum: granulate. Macrosculpture of mesoscutal midlobe: punctate rugose throughout. Macrosculpture of lateral lobe of mesoscutum: punctate rugose. Sculpture of notaulus: foveate. Notaular foveae: interconnected. Median mesoscutal carina: absent. Mesoscutellum in lateral view: convex. Sculpture of mesoscutellum: densely punctate rugose. Shape of metascutellum: trapezoidal with broad posterior margin. Median metascutellar carina: present. Sculpture of metascutellum: rugose. Sculpture of lateral propodeal area: rugose. Mesopleural carina: present. Sculpture of mesepisternum below femoral depression: punctate rugose. Sculpture of dorsal metapleural area: rugose. Setation of dorsal metapleural area: present. Setation of area directly dorsal to the metapleural triangle: absent. Sculpture of ventral metapleural area: rugose anteriorly, obliquely striate posteriorly. Color of legs: orange yellow throughout. Length of hind basitarsus: about as long as remaining segments combined. Sculpture of hind coxa: largely smooth, with sparse fine punctures.

Length of postmarginal vein: distinctly longer than stigmal vein.

Color of metasoma in female: black. Color of metasoma in male: black. Horn on T1 in female: present. Striae of posterior margin of T1 in female: dense. Striae of T1 in male: dense. Transverse sulcus on T2: present. Sculpture of T2: densely longitudinally striate, punctate rugulose in interstices. Sculpture of T6 in female: longitudinally punctate rugose. Length of T6 in female: approximately as long as wide; wider than long. Shape of T6 in female in lateral view: flat. Apical spine on female T6: absent. Sculpture of T6 in male: densely punctate. Sculpture of T7 in male: granulate. Posterior margin of T7 in male: straight. Sculpture of medial S2: densely longitudinally striate with fine punctures in interstices.

**Figures 224–229.**
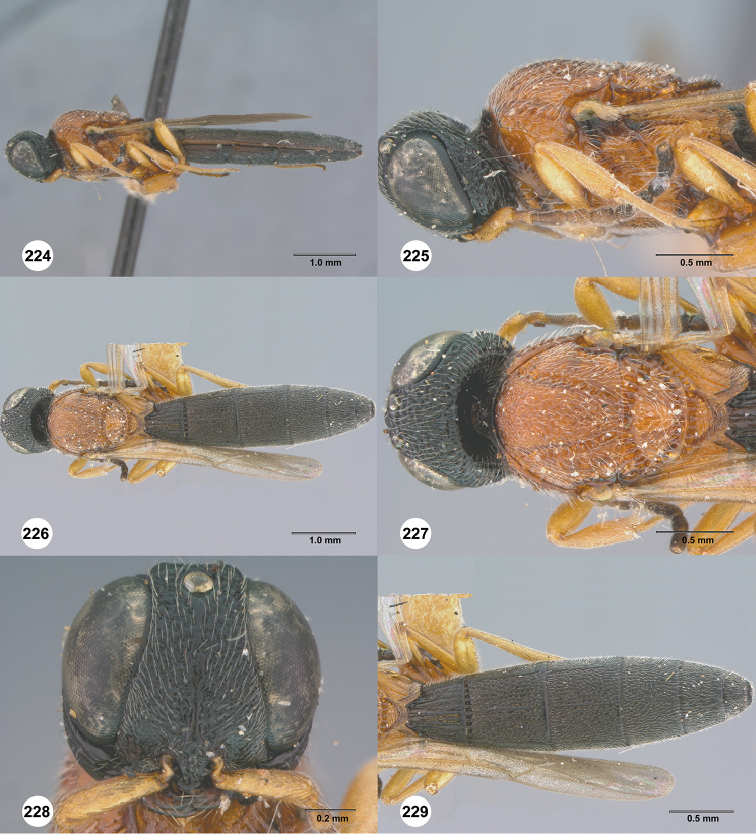
*Chromoteleiatricarinata* Kieffer, male, holotype (CAS TYPE9759). **224** Lateral habitus **225** Head and mesosoma, lateral view **226** Dorsal habitus **227** Head and mesosoma, dorsal view **228** Head, anterior view **229** Metasoma, dorsal view.

**Figures 230–235.**
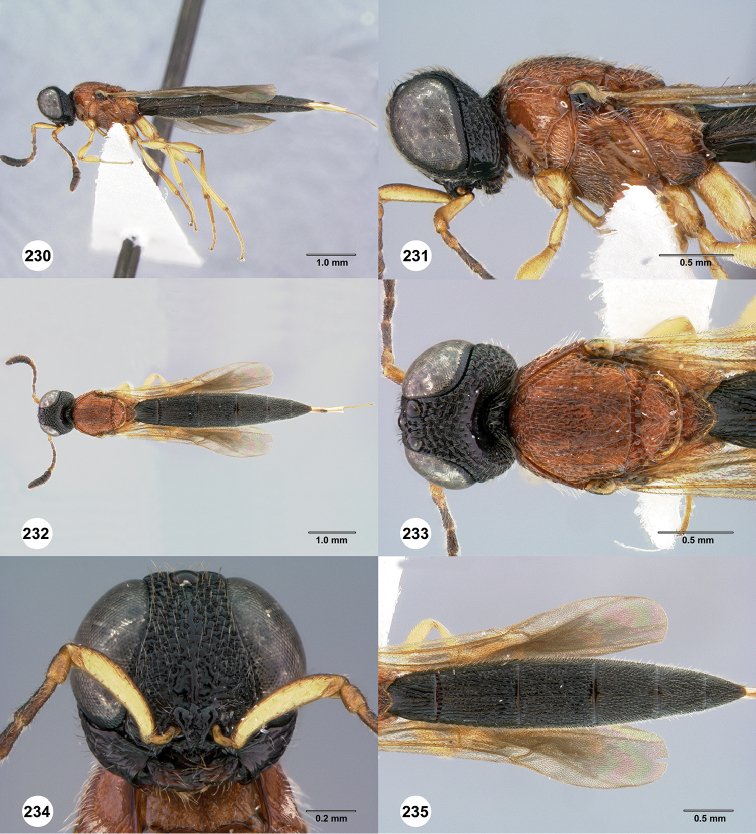
*Chromoteleiatricarinata* Kieffer, female (OSUC584805). **230** Lateral habitus **231** Head and mesosoma, lateral view **232** Dorsal habitus **233** Head and mesosoma, dorsal view **234** Head, anterior view **235** Metasoma, dorsal view.

**Figures 236–239.**
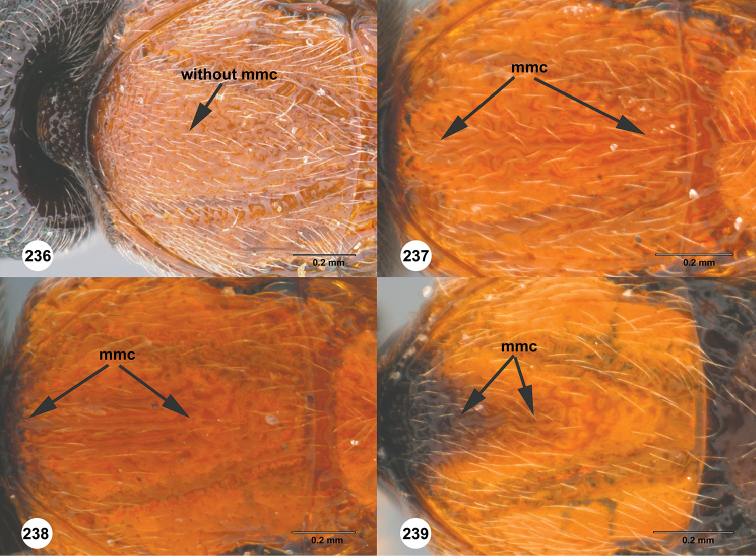
Mesoscutum, mmc: median mesoscutal carina **236***Chromoteleiatricarinata* Kieffer, male, holotype (CAS TYPE9759) **237***Chromoteleiaconnectens* Kieffer, female, holotype (CAS TYPE9618) **238***Chromoteleialongitarsis* Kieffer, male, holotype (CAS TYPE9682) **239***Chromoteleiacurta* sp. n., female, holotype (OSUC185626).

#### Diagnosis.

This species can be distinguished from other *Chromoteleia* by the following combination of characters: female A7 without basiconic sensillum, mesoscutum without median mesoscutal carina, dorsal metapleural area with setae, ventral metapleural area obliquely striate, male T7 with posterior margin straight.

#### Link to distribution map.

[http://hol.osu.edu/map-large.html?id=4218]

#### Material examined.

*Holotype*, male, *C.rufithorax var. tricarinata*: **BELIZE**: no date, CAS TYPE9759 (deposited in CAS).

**Other material**: (330 females, 551 males) **BELIZE**: 32 females, 27 males, OSUC556957, 584778–584798, 584800–584804, 584806, 584921, 584932–584934, 586445, 586758, 586870–586871 (CNCI); OSUC186068, 186070, 225287, 232140–232142, 47918–47919, 47940, 48011, 48037, 64021, 64026–64028, 64046–64047, 93534, 93586–93588, 94076–94077 (OSUC). **BRAZIL**: 1 female, 2 males, OSUC 56227–56229 (OSUC). **COLOMBIA**: 1 female, OSUC586287 (CNCI). **COSTA RICA**: 191 females, 340 males, OSUC149653, 149657, 202538 (AEIC); OSUC232992 (BPBM); OSUC232058, 232120–232138, 232188, 232190–232191, 374050, 556994, 556998, 557003, 557007, 557009, 557015, 557017, 557020–557022, 557024–557029, 557046–557047, 557052, 557054–557057, 557059–557060, 557063–557080, 557082, 577894, 577897, 577900–577901, 577905, 577925, 577928, 577930–577931, 577942, 577952, 577956–577961, 577963, 577965–577970, 577972, 578010–578011, 578019, 578023–578024, 578044, 578046, 578053, 578059, 578063–578065, 578067–578070, 578072, 578074–578075, 578077–578078, 578084, 578087–578090, 578093–578096, 578098, 578100–578102, 578105, 583414, 583417–583418, 583421, 583429, 583437, 583460, 583462, 583465–583466, 583470, 583514–583515, 583518–583520, 583709–583715, 583718, 583720–583722, 583724–583730, 583732–583738, 583740–583750, 583754–583755, 583759, 583761–583762, 583766–583767, 583772, 583774–583775, 583777–583783, 583786, 583788–583793, 583795, 583797–583798, 583818–583828, 583830–583833, 583835–583849, 583851–583877, 583883–583885, 583888, 583893–583895, 583899–583900, 583902–583909, 583911–583912, 583914–583915, 583917–583924, 583929–583930, 583939, 583941–583942, 583945–583946, 583950–583951, 583977, 583979, 583983–583987, 583991, 583995–583996, 583999–584002, 584007, 584013, 584015, 584018, 584020–584022, 584024, 584029–584030, 584034, 584036, 584040, 584043–584044, 584046, 584138, 584225, 584227, 584230, 584233, 584240, 584242, 584244, 584250, 584264–584270, 584274–584276, 584281–584283, 584287, 585121–585124, 585126–585144, 586127–586128, 586139–586141, 586152–586156, 586165, 586172, 586174, 586184, 586195, 586197, 586199, 586202, 586204, 586207–586208, 586211, 586215, 586218–586221, 586262–586266, 586270, 586275–586286, 586291–586292, 586296–586301, 586374–586375, 586379–586389, 586391, 586405, 586407–586410, 586414, 586473, 586481–586484, 586486, 586488, 586490–586492, 586495–586497, 586506, 586508, 586511, 586514–586515, 586535–586537, 586540, 586560–586564, 586566, 586569–586571, 586628, 586630, 586632, 586634, 586636–586637, 586642–586643, 586647, 586650–586655, 586657, 586660–586661, 586663, 586665–586666, 586671, 586763, 586765–586768, 586779, 586835, 586872 (CNCI); DPI_FSCA 00010211 (CSCA); SM0810075, SM0810348T (KUNH); OSUC185844, 374051–374054 (OSUC); OSUC320638, 320648–320650 (TAMU); OSUC204982, 204999 (UCDC); OSUC157812 (UCMC). **GUATEMALA**: 9 females, 16 males, OSUC584920, 584922–584924, 584926–584931, 584935–584936, 584940–584948, 584950, 584952–584953 (CNCI); OSUC317963 (OSUC). **HONDURAS**: 13 females, 16 males, OSUC584961–584963 (CNCI); OSUC369620, 369622, 369624, 369626–369627, 413758–413759, 413761–413763, 413766–413771, 413773–413782 (MZLU). **MEXICO**: 14 females, 20 males, OSUC556947, 584937–584939, 584949, 584951, 584954–584960 (CNCI); OSUC268812–268813, 268815–268818, 271011, 271013–271014, 271016, 56230–56233, 56235, 56237–56241, 56243 (OSUC). **NICARAGUA**: 1 female, 1 male, DPI_FSCA 00010212 (CSCA); OSUC204951 (UCDC). **PANAMA**: 68 females, 128 males, OSUC149652, 149655, 202542–202543, 202557, 202559, 202561–202575 (AEIC); OSUC557099–557112, 578054, 583459, 583483, 583501, 583505–583507, 584179–584181, 584183–584224, 584337–584373, 584375–584376, 584383–584397, 586158, 586345, 586373, 586377, 586465–586468, 586477–586479, 586521–586527, 586565, 586613–586617, 586627, 586631, 586682–586692, 586815–586817, 586820–586823, 586825, 586874 (CNCI); OSUC221922–221923, 319208–319209, 320642, 320645–320647, 321353 (TAMU). **VENEZUELA**: 1 male, OSUC557086 (CNCI).

## Supplementary Material

XML Treatment for Chromoteleia

XML Treatment for Chromoteleia aequalis

XML Treatment for Chromoteleia alternata

XML Treatment for Chromoteleia bidens

XML Treatment for Chromoteleia congoana

XML Treatment for Chromoteleia connectens

XML Treatment for Chromoteleia copiosa

XML Treatment for Chromoteleia cuneus

XML Treatment for Chromoteleia curta

XML Treatment for Chromoteleia depilis

XML Treatment for Chromoteleia dispar

XML Treatment for Chromoteleia feng

XML Treatment for Chromoteleia fossa

XML Treatment for Chromoteleia fuscicornis

XML Treatment for Chromoteleia ingens

XML Treatment for Chromoteleia levitas

XML Treatment for Chromoteleia longitarsis

XML Treatment for Chromoteleia longa

XML Treatment for Chromoteleia maura

XML Treatment for Chromoteleia parvitas

XML Treatment for Chromoteleia pilus

XML Treatment for Chromoteleia plana

XML Treatment for Chromoteleia rara

XML Treatment for Chromoteleia robusta

XML Treatment for Chromoteleia rufithorax

XML Treatment for Chromoteleia semicyanea

XML Treatment for Chromoteleia semilutea

XML Treatment for Chromoteleia sparsa

XML Treatment for Chromoteleia tricarinata

## Appendix 1

URI Table matching terms and concepts used in this revision with the Hymenoptera Anatomy Ontology database.

**Table:**

	A1	http://purl.obolibrary.org/obo/HAO_0000908
	A2	http://purl.obolibrary.org/obo/HAO_0000706
	A3	http://purl.obolibrary.org/obo/HAO_0001148
	A7	http://purl.obolibrary.org/obo/HAO_0001885
	A12	http://purl.obolibrary.org/obo/HAO_0001884
	antenna	http://purl.obolibrary.org/obo/HAO_0000101
	antennomere	http://purl.obolibrary.org/obo/HAO_0000107
	area	http://purl.obolibrary.org/obo/HAO_0000146
	body	http://purl.obolibrary.org/obo/HAO_0000182
	carina	http://purl.obolibrary.org/obo/HAO_0000188
	central keel	http://purl.obolibrary.org/obo/HAO_0000109
cpa	cervical pronotal area	http://purl.obolibrary.org/obo/HAO_0000194
	clava	http://purl.obolibrary.org/obo/HAO_0000203
	clypeus	http://purl.obolibrary.org/obo/HAO_0000212
	compound eye	http://purl.obolibrary.org/obo/HAO_0000217
	coxa	http://purl.obolibrary.org/obo/HAO_0000228
	depression	http://purl.obolibrary.org/obo/HAO_0000241
dpa	dorsal pronotal area	http://purl.obolibrary.org/obo/HAO_0000267
	egg	http://purl.obolibrary.org/obo/HAO_0000286
	epomial carina	http://purl.obolibrary.org/obo/HAO_0000307
	eye	http://purl.obolibrary.org/obo/HAO_0000217
	femur	http://purl.obolibrary.org/obo/HAO_0000327
	fore wing	http://purl.obolibrary.org/obo/HAO_0000351
	frons	http://purl.obolibrary.org/obo/HAO_0001523
	gena	http://purl.obolibrary.org/obo/HAO_0000371
	head	http://purl.obolibrary.org/obo/HAO_0000397
	hind coxa	http://purl.obolibrary.org/obo/HAO_0000587
	hind tibia	http://purl.obolibrary.org/obo/HAO_0000631
	hind wing	http://purl.obolibrary.org/obo/HAO_0000400
	inner orbit	http://purl.obolibrary.org/obo/HAO_0000419
	interantennal process	http://purl.obolibrary.org/obo/HAO_0000422
	lateral lobe of mesoscutum	http://purl.obolibrary.org/obo/HAO_0000466
	lateral ocellus	http://purl.obolibrary.org/obo/HAO_0000481
LOL	lateral ocellar line	http://purl.obolibrary.org/obo/HAO_0000480
lpa	lateral pronotal area	http://purl.obolibrary.org/obo/HAO_0000483
	malar sulcus	http://purl.obolibrary.org/obo/HAO_0000504
	mandible	http://purl.obolibrary.org/obo/HAO_0000506
	margin	http://purl.obolibrary.org/obo/HAO_0000510
	mesepisternum	http://purl.obolibrary.org/obo/HAO_0001872
med	mesopleural depression	http://purl.obolibrary.org/obo/HAO_0000326
	mesopleuron	http://purl.obolibrary.org/obo/HAO_0000566
	mesoscutellum	http://purl.obolibrary.org/obo/HAO_0000574
	mesoscutum	http://purl.obolibrary.org/obo/HAO_0001490
	mesosoma	http://purl.obolibrary.org/obo/HAO_0000576
	metapleuron	http://purl.obolibrary.org/obo/HAO_0000621
	metascutellum	http://purl.obolibrary.org/obo/HAO_0000625
	metasoma	http://purl.obolibrary.org/obo/HAO_0000626
	midlobe of mesoscutum	http://purl.obolibrary.org/obo/HAO_0000520
	netrion	http://purl.obolibrary.org/obo/HAO_0000644
	notauli (notaulus)	http://purl.obolibrary.org/obo/HAO_0000647
	occipital carina	http://purl.obolibrary.org/obo/HAO_0000653
	ocellus	http://purl.obolibrary.org/obo/HAO_0000661
ot	ocellar triangle	http://purl.obolibrary.org/obo/HAO_0000430
OOL	ocular ocellar line	http://purl.obolibrary.org/obo/HAO_0000662
	orbit	http://purl.obolibrary.org/obo/HAO_0000672
POL	posterior ocellar line	http://purl.obolibrary.org/obo/HAO_0000759
	process	http://purl.obolibrary.org/obo/HAO_0000822
	propodeum	http://purl.obolibrary.org/obo/HAO_0001248
	S1	http://purl.obolibrary.org/obo/HAO_0001997
	S2	http://purl.obolibrary.org/obo/HAO_0001829
	S3	http://purl.obolibrary.org/obo/HAO_0001831
	S4	http://purl.obolibrary.org/obo/HAO_0001832
	S5	http://purl.obolibrary.org/obo/HAO_0001833
	S6	http://purl.obolibrary.org/obo/HAO_0001834
	S7	http://purl.obolibrary.org/obo/HAO_0002185
	sculpture	http://purl.obolibrary.org/obo/HAO_0000913
	sternite	http://purl.obolibrary.org/obo/HAO_0001654
	sulcus	http://purl.obolibrary.org/obo/HAO_0000978
	T1	http://purl.obolibrary.org/obo/HAO_0000053
	T2	http://purl.obolibrary.org/obo/HAO_0000056
	T3	http://purl.obolibrary.org/obo/HAO_0000057
	T4	http://purl.obolibrary.org/obo/HAO_0000058
	T5	http://purl.obolibrary.org/obo/HAO_0000059
	T6	http://purl.obolibrary.org/obo/HAO_0000060
	T7	http://purl.obolibrary.org/obo/HAO_0000061
	tergite	http://purl.obolibrary.org/obo/HAO_0001783
	tibia	http://purl.obolibrary.org/obo/HAO_0001017
	tyloid	http://purl.obolibrary.org/obo/HAO_0001199
	vein	http://purl.obolibrary.org/obo/HAO_0001095
	vertex	http://purl.obolibrary.org/obo/HAO_0001077
	vertical epomial carina	http://purl.obolibrary.org/obo/HAO_0000307
	wing	http://purl.obolibrary.org/obo/HAO_0001089
